# Supplementary material for: Telomere length regulation by Rif1 protein from Hansenula polymorpha
Source: eLife. 2022 Feb 7;11:e75010. doi: 10.7554/eLife.75010 (PMC8820739; doi:10.7554/eLife.75010)
Supplement: Figure 1—source data 2. [file elife-75010-fig1-data2.zip › Figure 1 - source data 2/Fig. 1 labels.pdf]

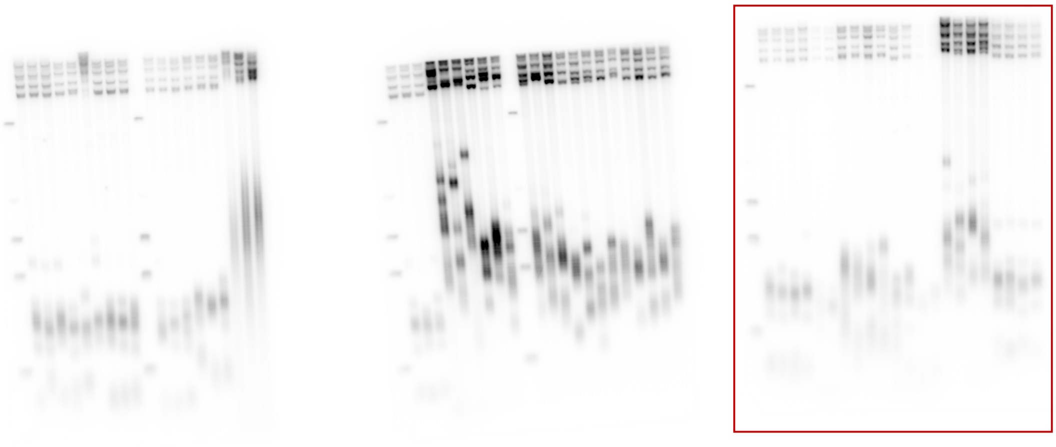

Red square marks the area shown in Figure 1B

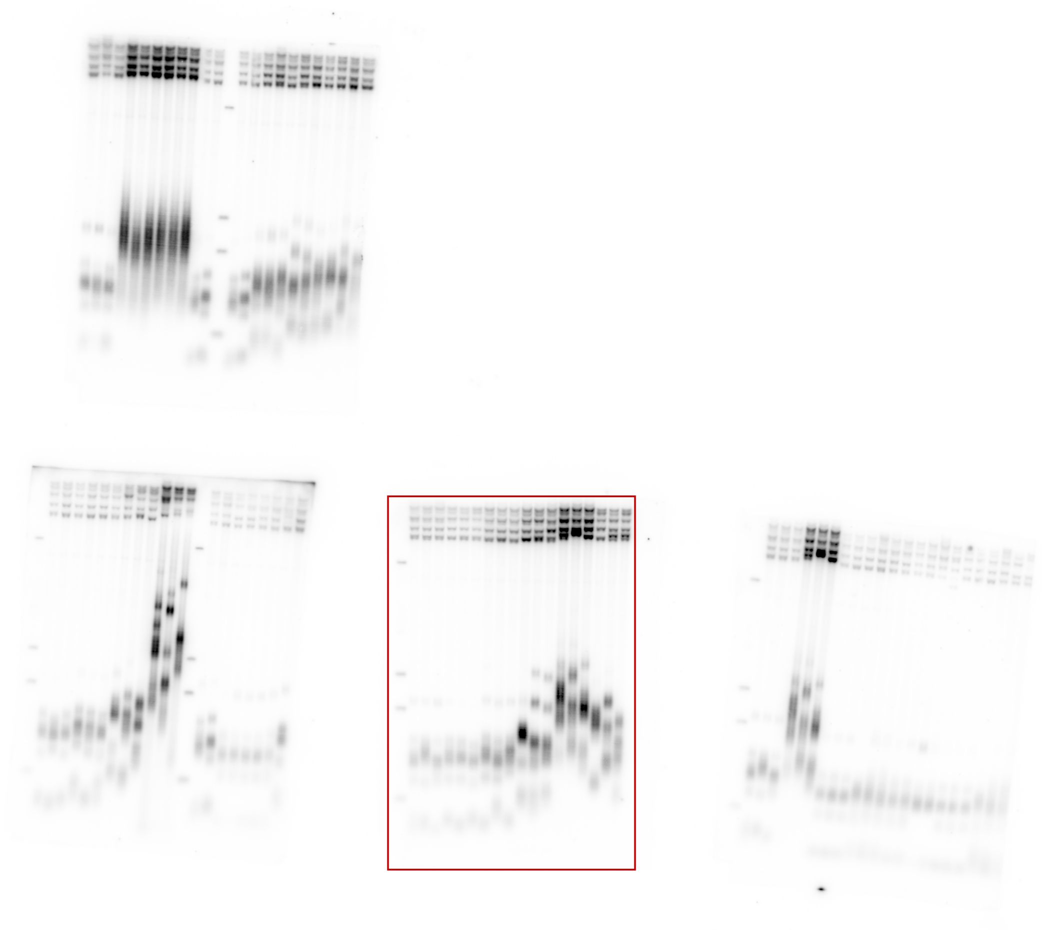

Red square marks the area shown in Figure 1E (the raw unedited blot is in the Figure 1 - source data 3 folder).

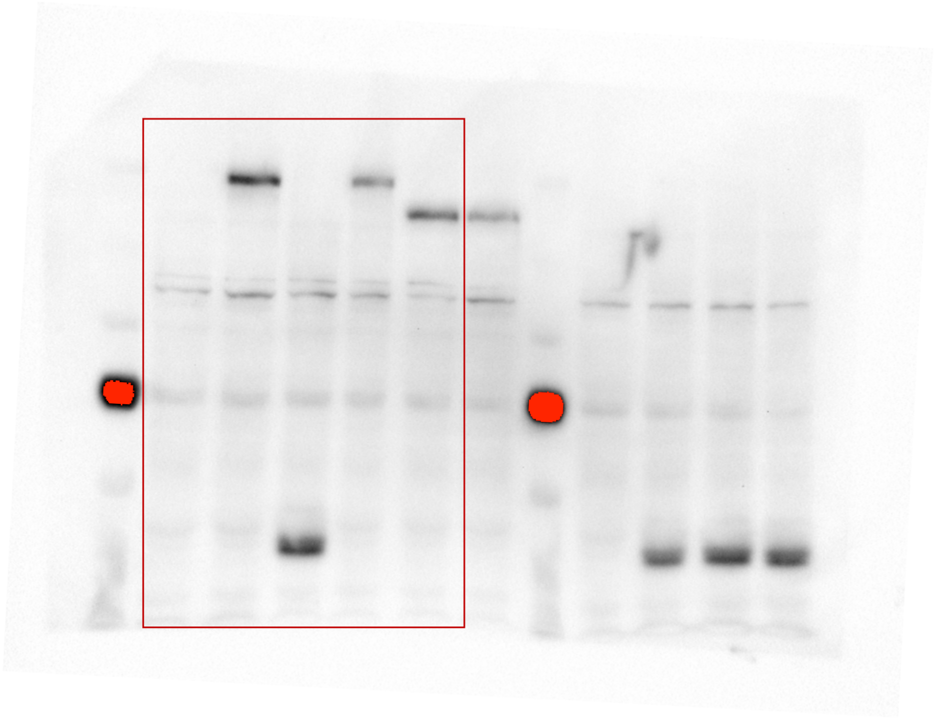

Red square marks the area shown in Figure 1F (**α**-HA blot)

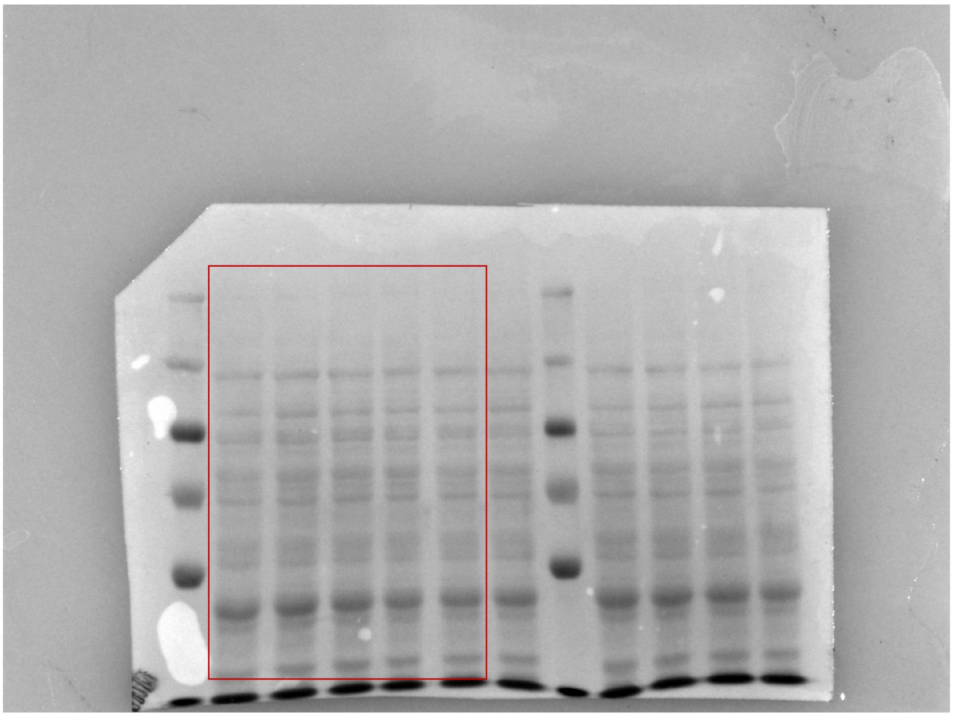

Red square marks the area shown in Figure 1F (ponceau)
