# Supplementary figures and images for: Telomere length regulation by Rif1 protein from Hansenula polymorpha
Source: eLife. 2022 Feb 7;11:e75010. doi: 10.7554/eLife.75010 (PMC8820739; doi:10.7554/eLife.75010)

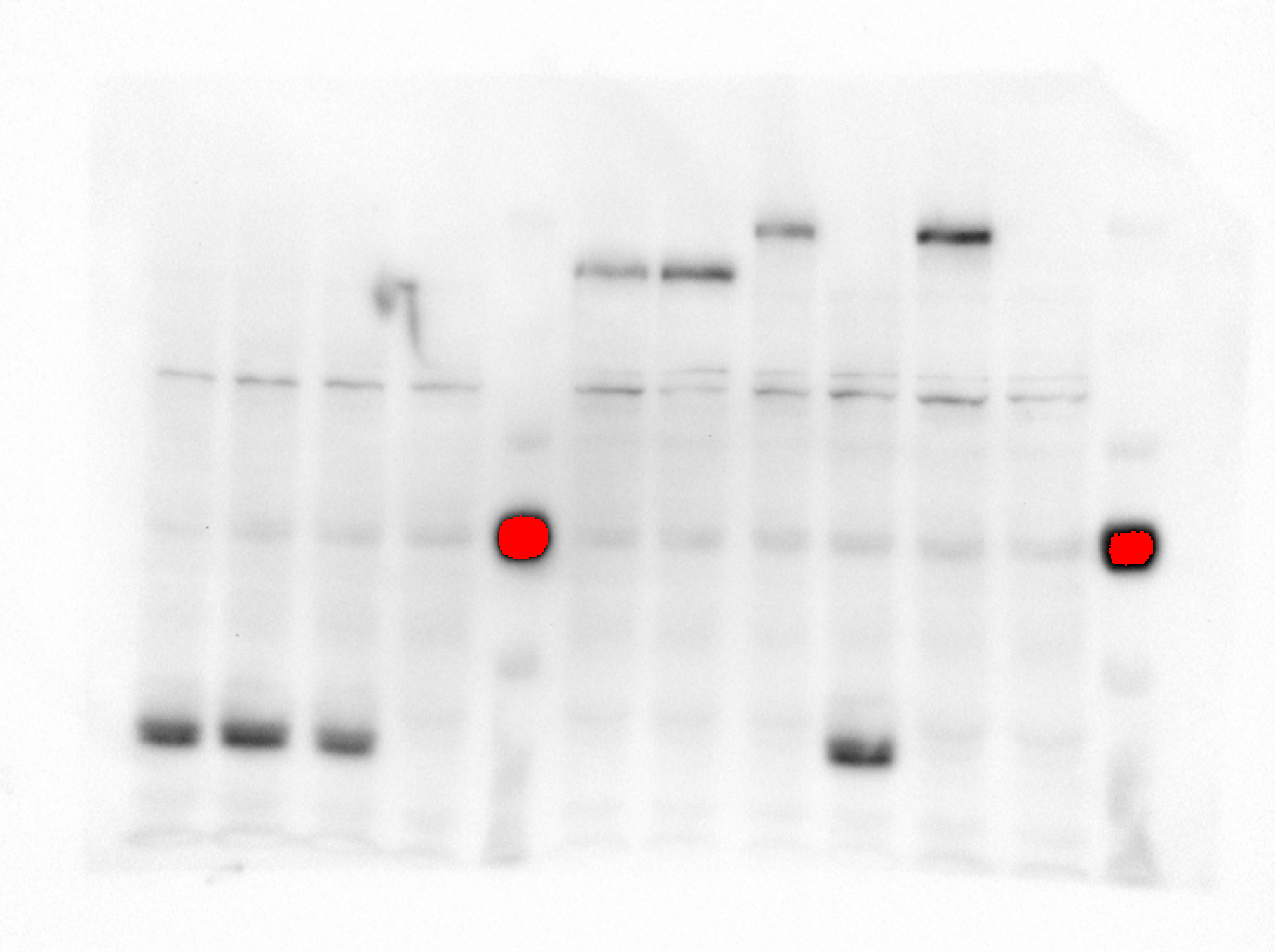

Supplement: Figure 1—source data 2. [file elife-75010-fig1-data2.zip › Figure 1 - source data 2/Fig. 1F (HA blot).tif]

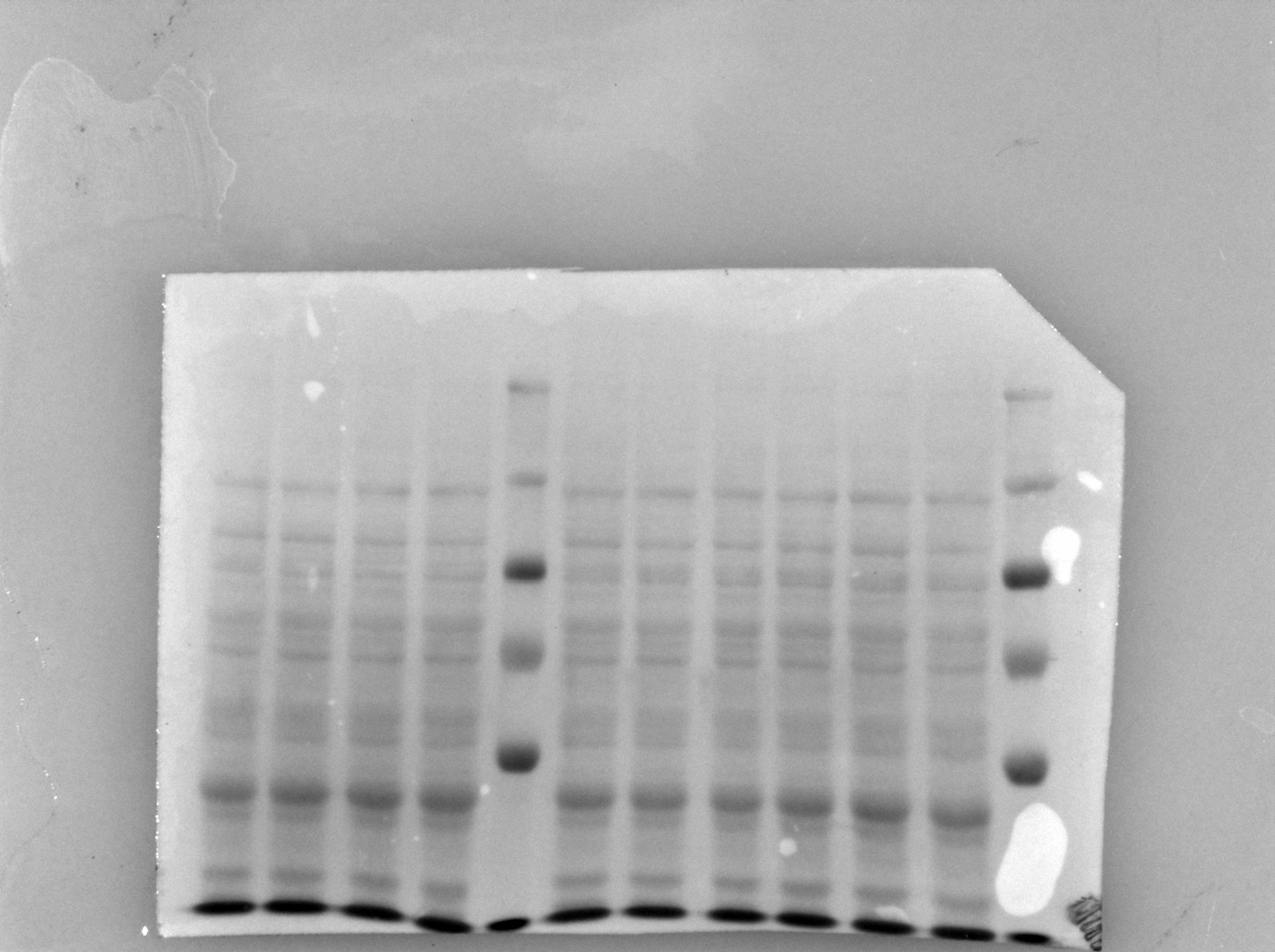

Supplement: Figure 1—source data 2. [file elife-75010-fig1-data2.zip › Figure 1 - source data 2/Fig. 1F (ponceau).tif]

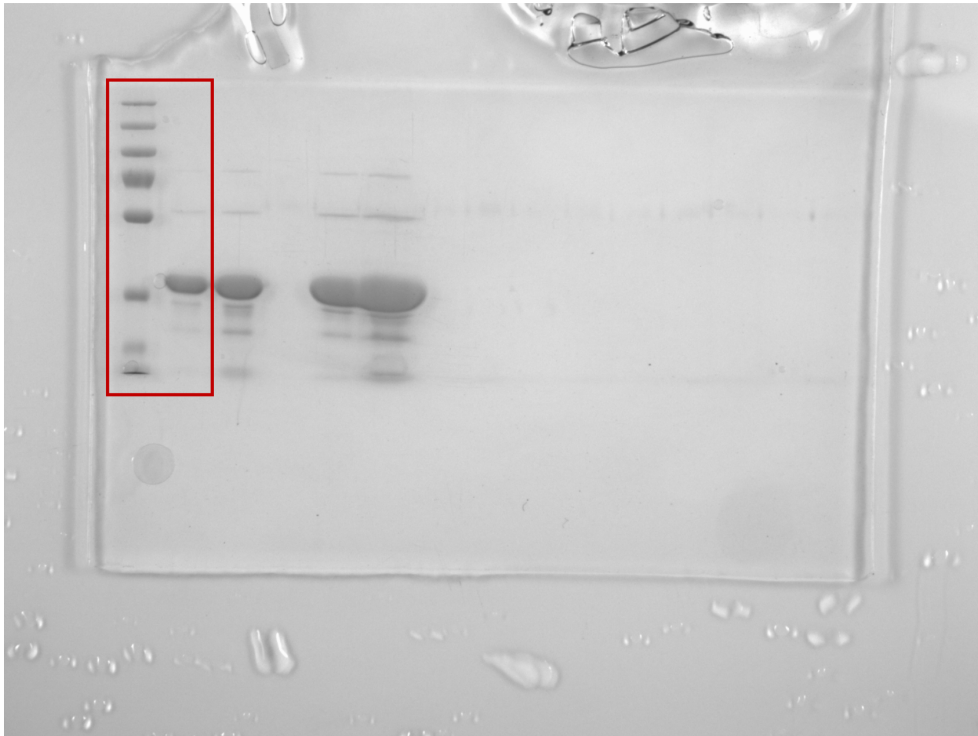

Red square marks the area shown in Figure 2B.

Supplement: Figure 2—source data 1. [file elife-75010-fig2-data1.zip › Figure 2 - source data 1/Fig. 2B labels.pdf]

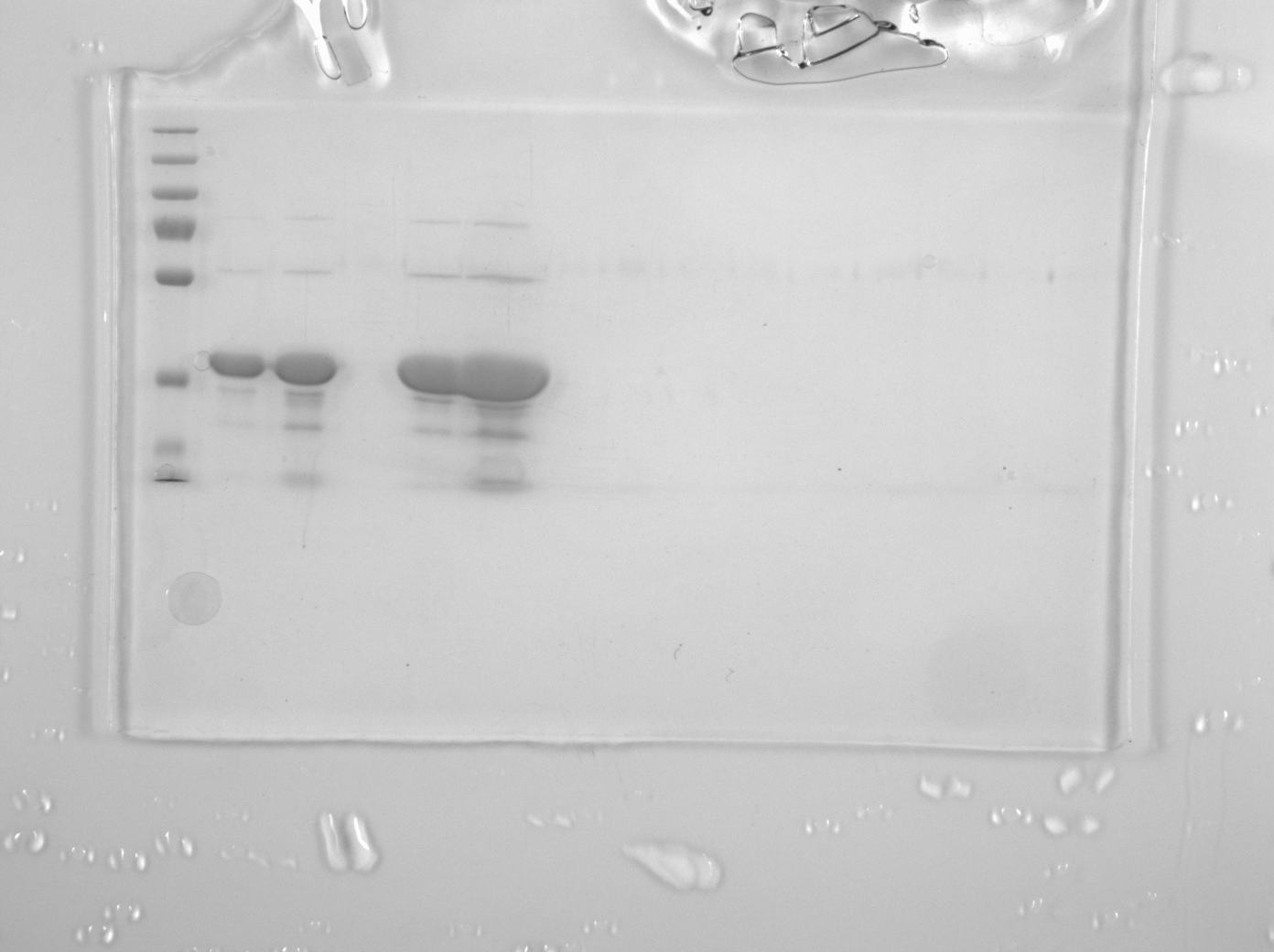

Supplement: Figure 2—source data 1. [file elife-75010-fig2-data1.zip › Figure 2 - source data 1/Fig. 2B.tif]

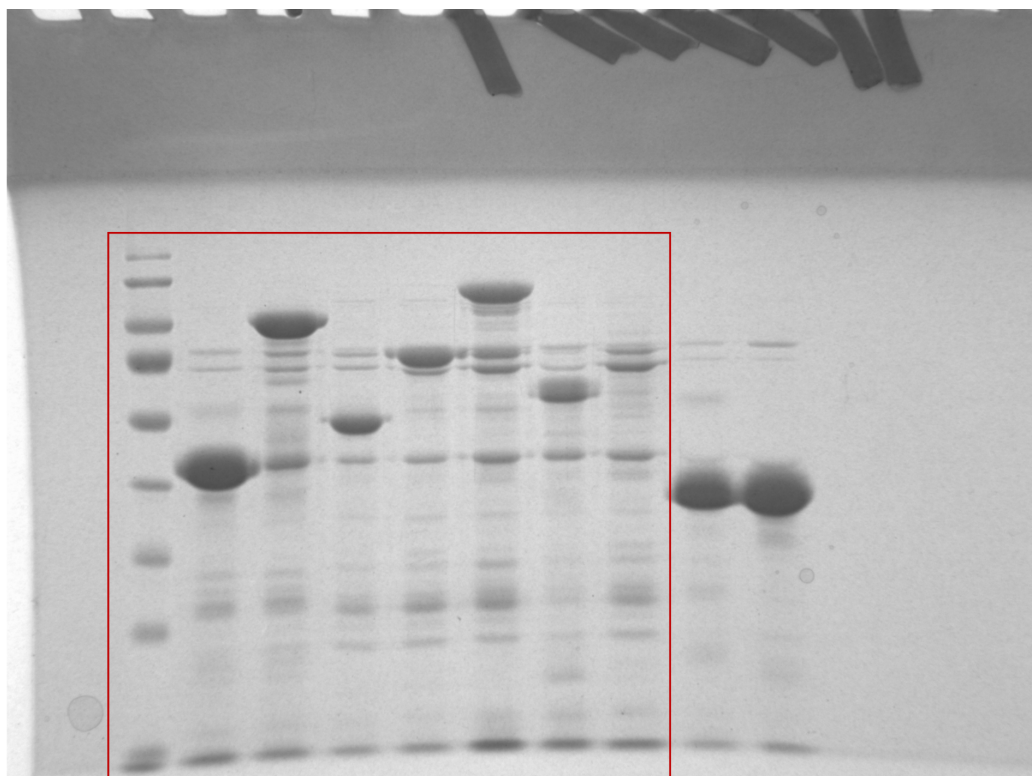

Red square marks the area shown in Figure 3B.

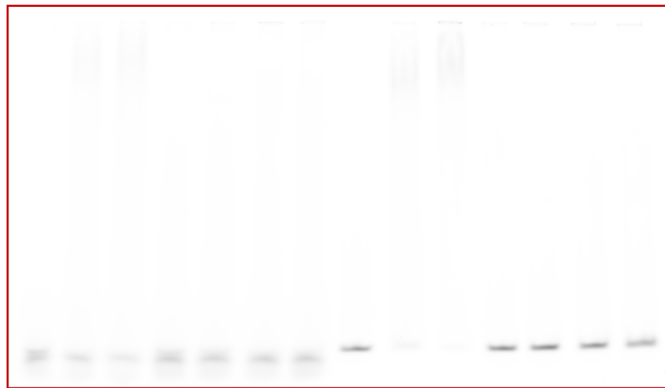

Red square marks the area shown in Figure 3C.

Supplement: Figure 3—source data 2. [file elife-75010-fig3-data2.zip › Figure 3 - source data 2/Fig. 3 labels.pdf]

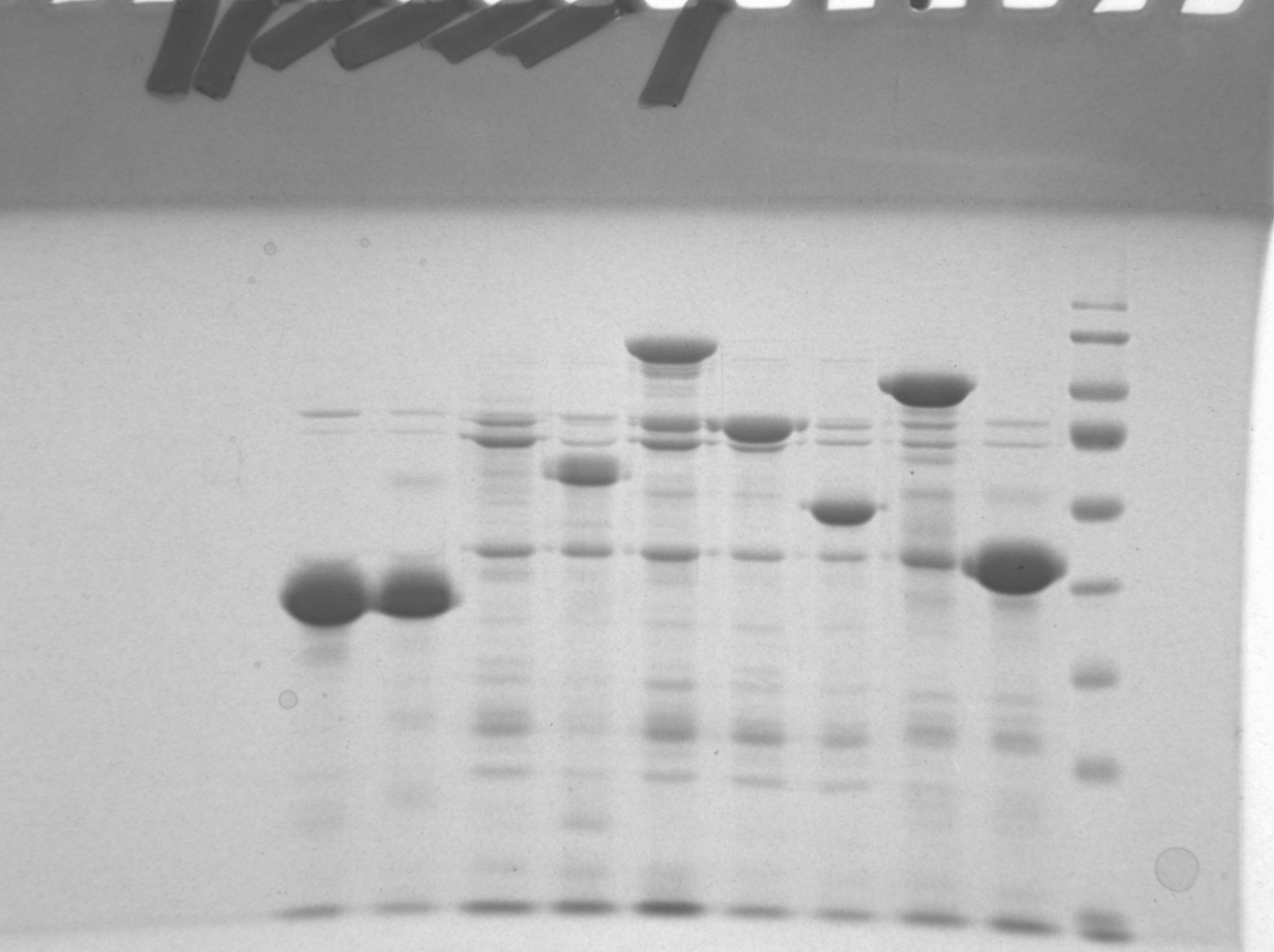

Supplement: Figure 3—source data 2. [file elife-75010-fig3-data2.zip › Figure 3 - source data 2/Fig. 3B.tif]

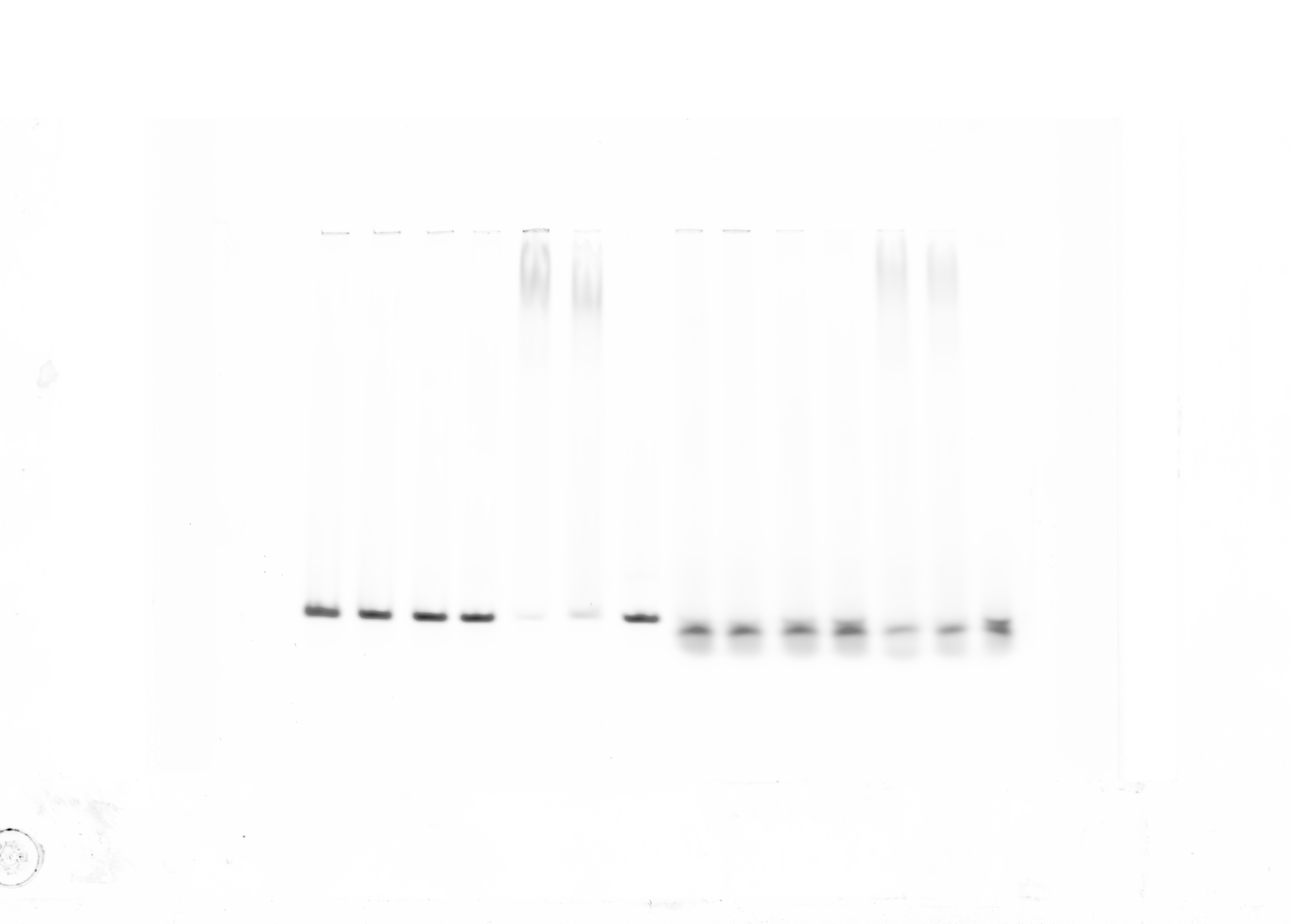

Supplement: Figure 3—source data 2. [file elife-75010-fig3-data2.zip › Figure 3 - source data 2/Fig. 3C.tif]

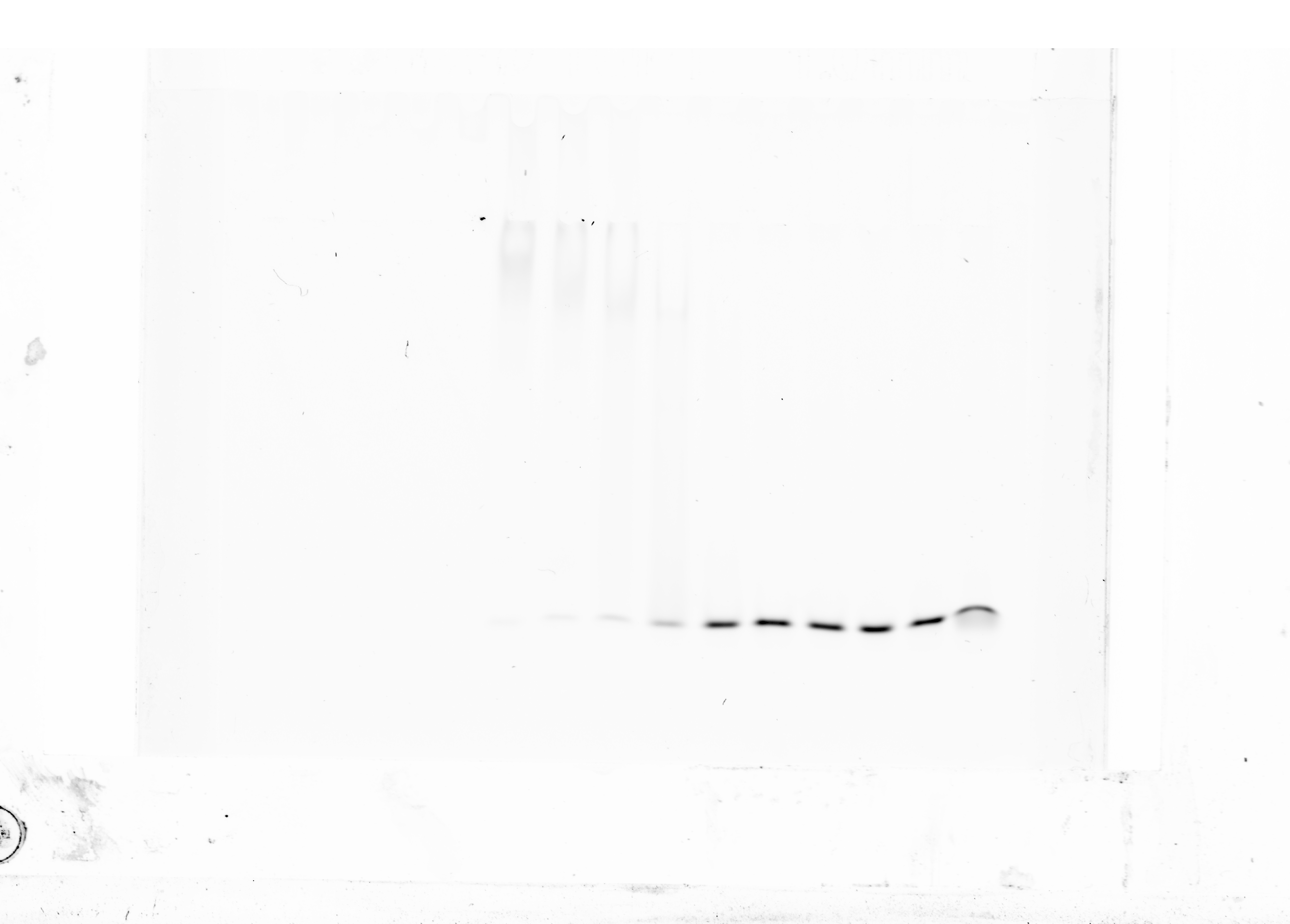

Supplement: Figure 3—figure supplement 1—source data 1. [file elife-75010-fig3-figsupp1-data1.zip › Figure 3 - figure supplement 1 - source data 1/Fig. 3 - suppl. 1A Tel4(G).tif]

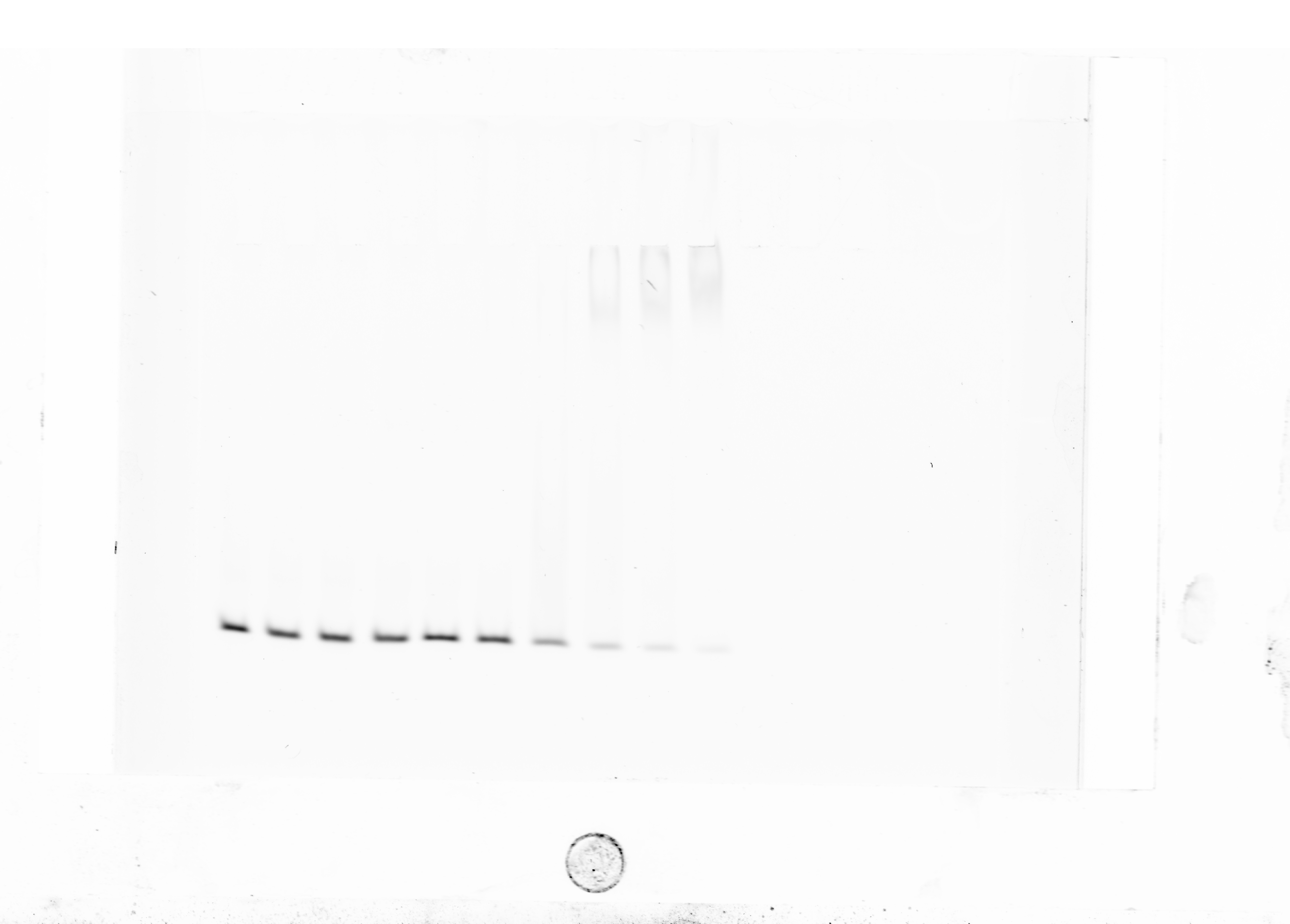

Supplement: Figure 3—figure supplement 1—source data 1. [file elife-75010-fig3-figsupp1-data1.zip › Figure 3 - figure supplement 1 - source data 1/Fig. 3 - suppl. 1A Tel4(GC).tif]

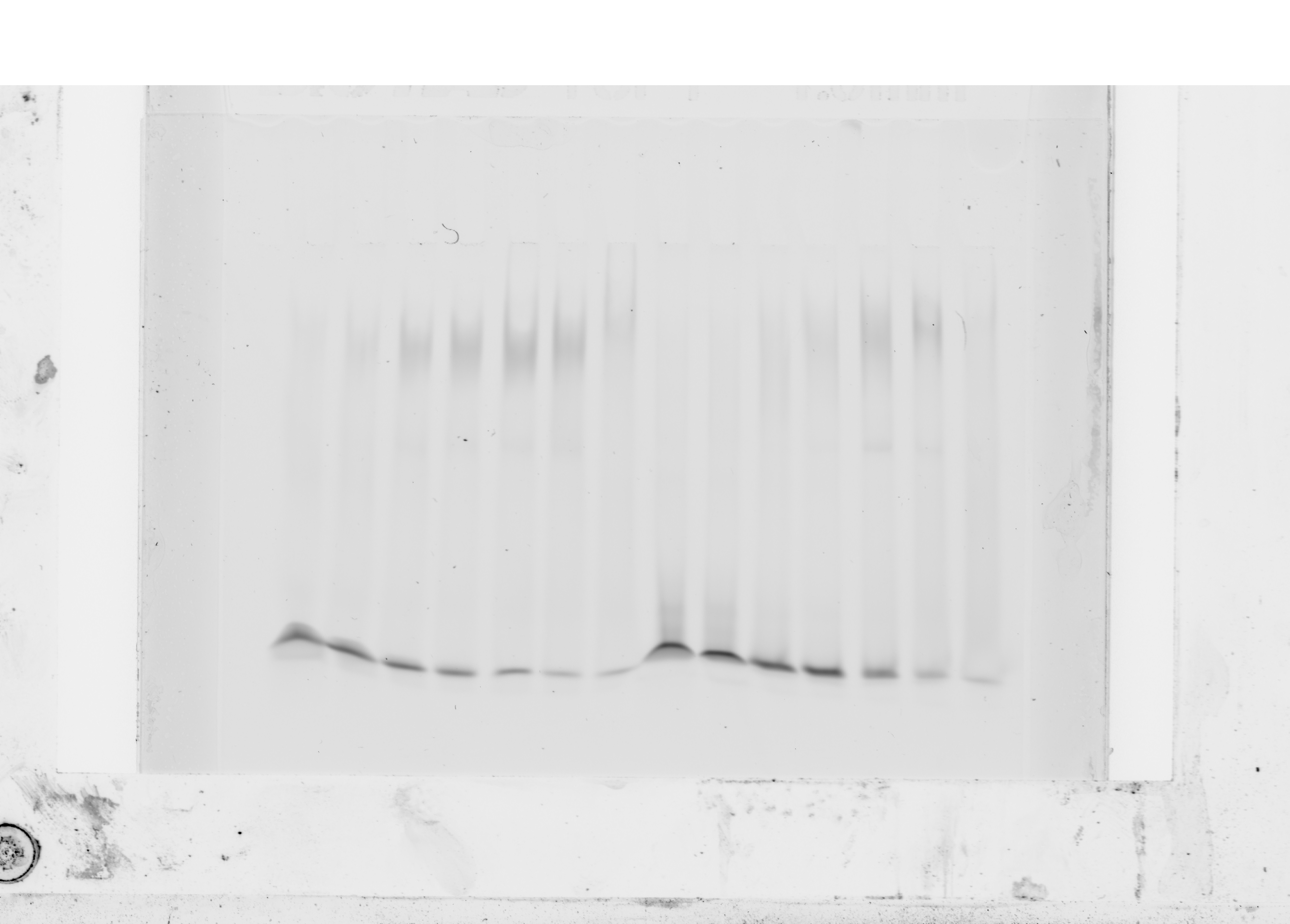

Supplement: Figure 3—figure supplement 1—source data 1. [file elife-75010-fig3-figsupp1-data1.zip › Figure 3 - figure supplement 1 - source data 1/Fig. 3 - suppl. 1B dsrnd1.tif]

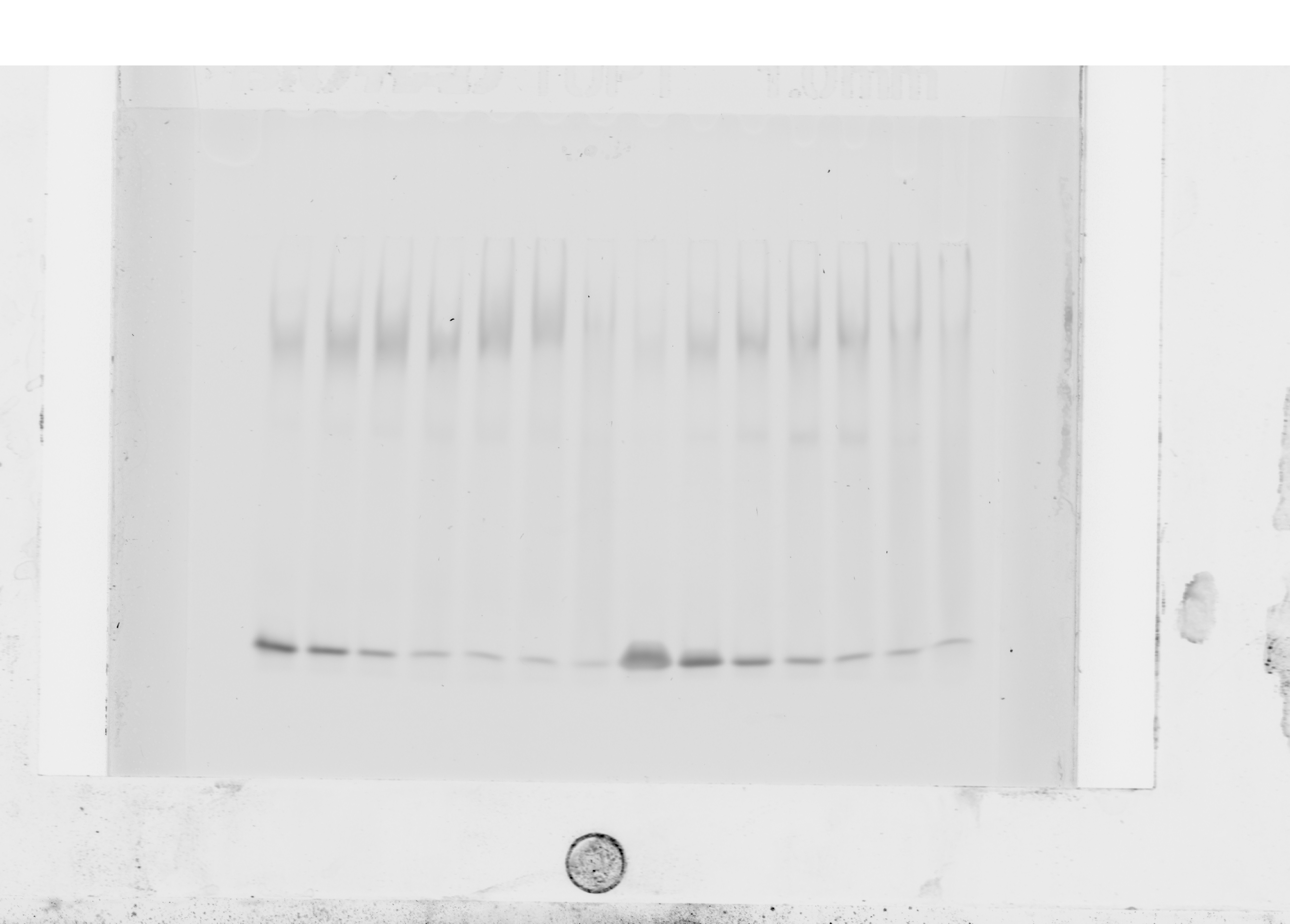

Supplement: Figure 3—figure supplement 1—source data 1. [file elife-75010-fig3-figsupp1-data1.zip › Figure 3 - figure supplement 1 - source data 1/Fig. 3 - suppl. 1B ssrnd2 ssrnd3.tif]

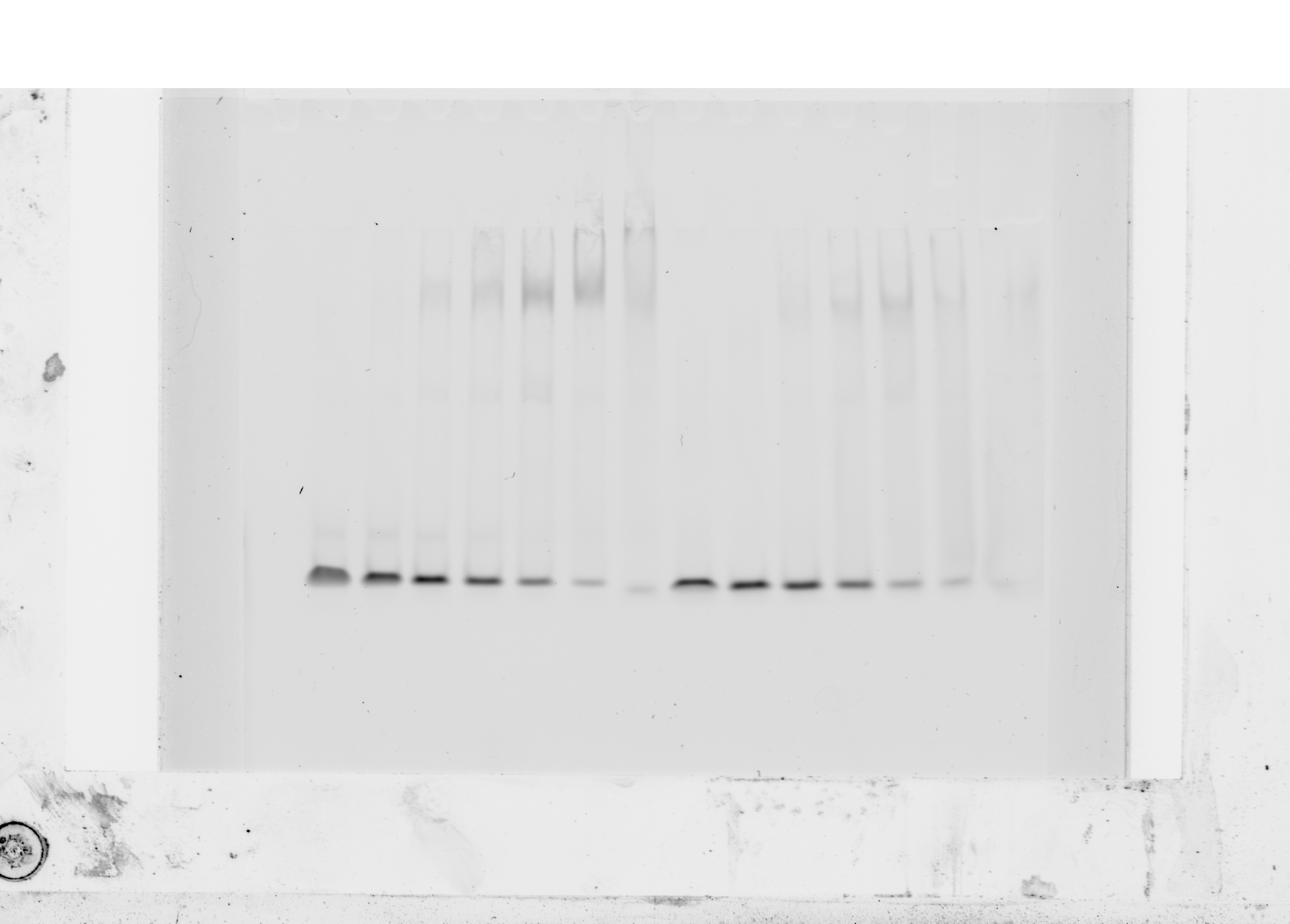

Supplement: Figure 3—figure supplement 1—source data 1. [file elife-75010-fig3-figsupp1-data1.zip › Figure 3 - figure supplement 1 - source data 1/Fig. 3 - suppl. 1B Tel4(C).tif]

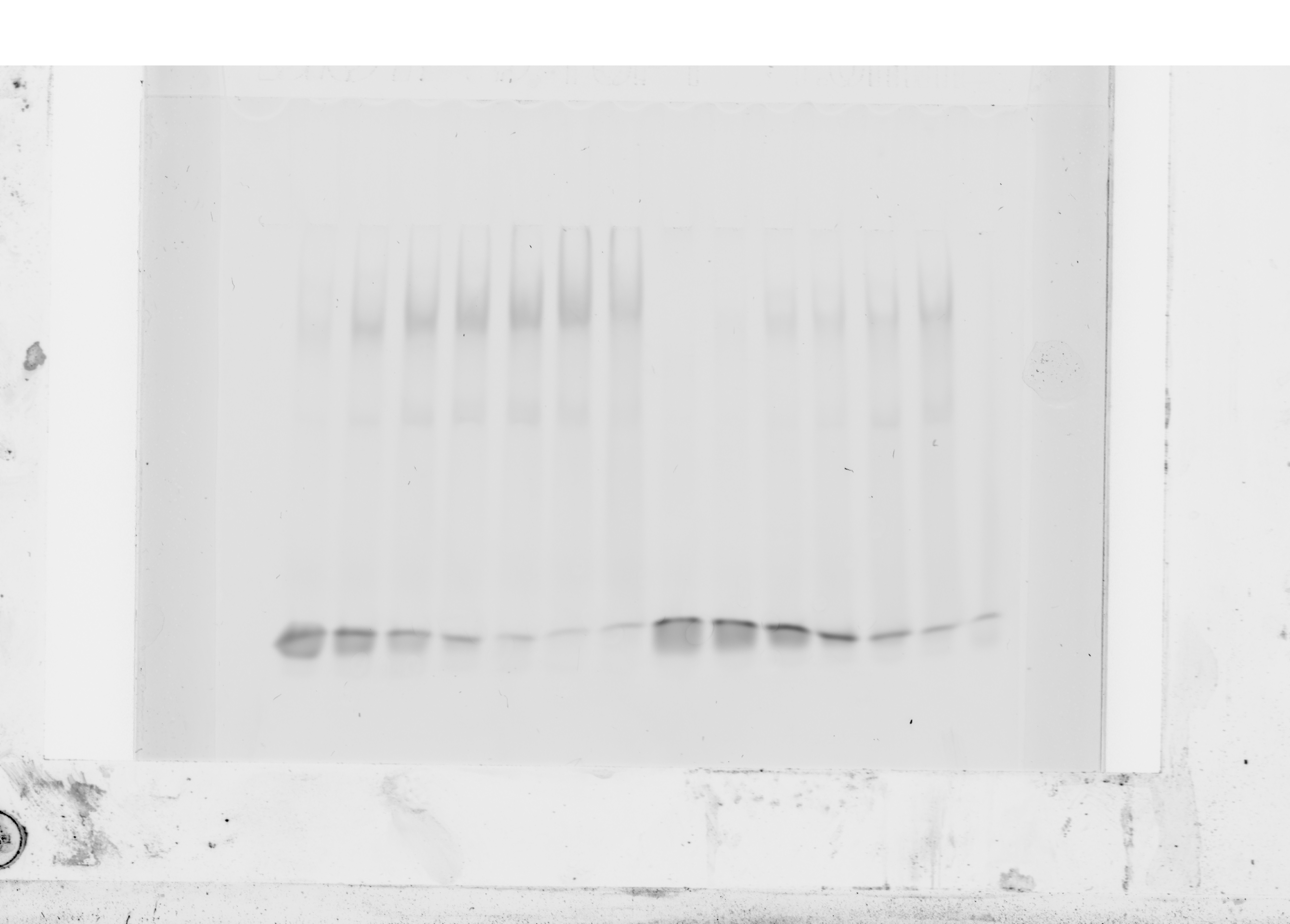

Supplement: Figure 3—figure supplement 1—source data 1. [file elife-75010-fig3-figsupp1-data1.zip › Figure 3 - figure supplement 1 - source data 1/Fig. 3 - suppl. 1B Tel4(G) ssrnd1.tif]

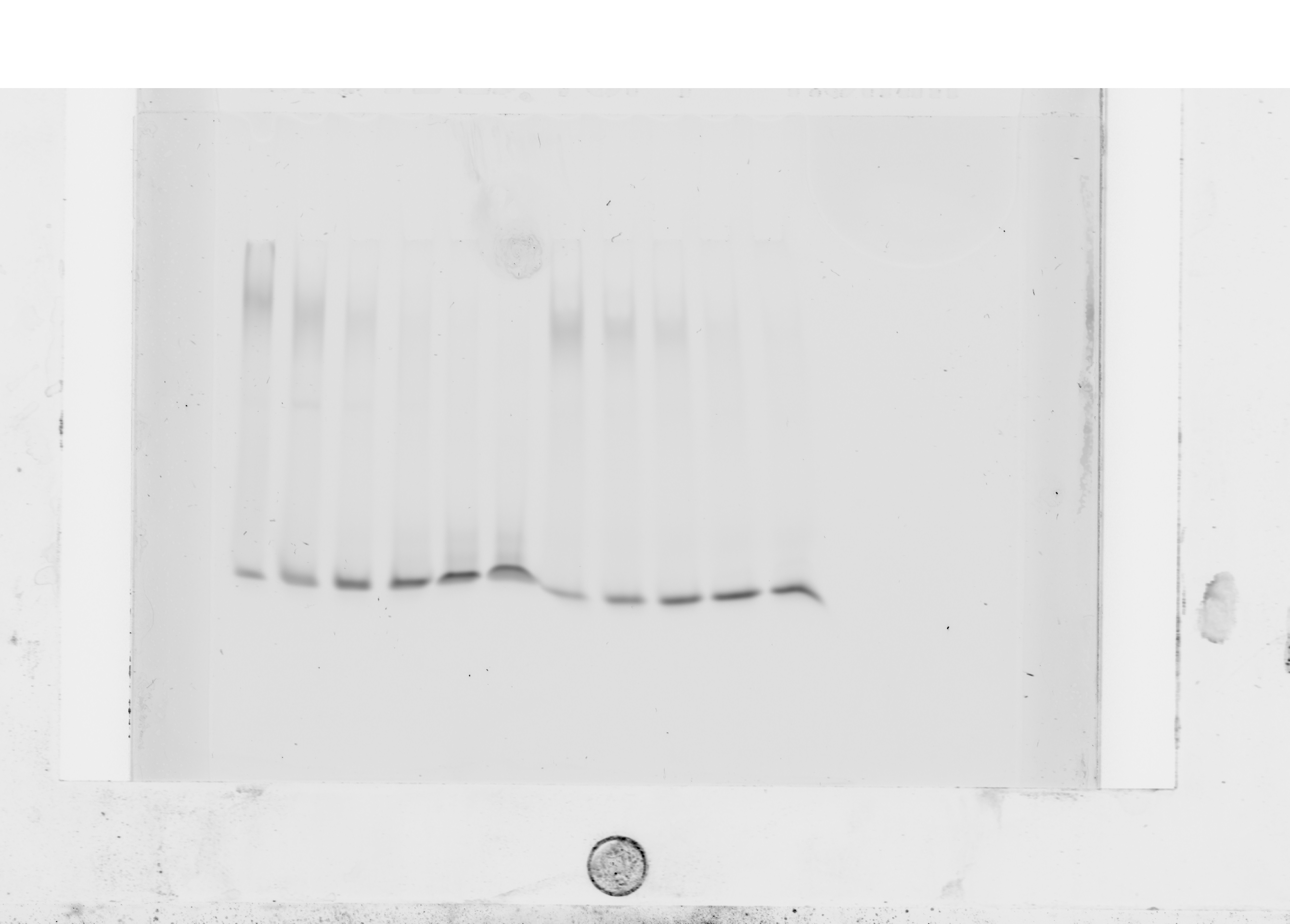

Supplement: Figure 3—figure supplement 1—source data 1. [file elife-75010-fig3-figsupp1-data1.zip › Figure 3 - figure supplement 1 - source data 1/Fig. 3 - suppl. 1B Tel4(GC) dsrnd2.tif]

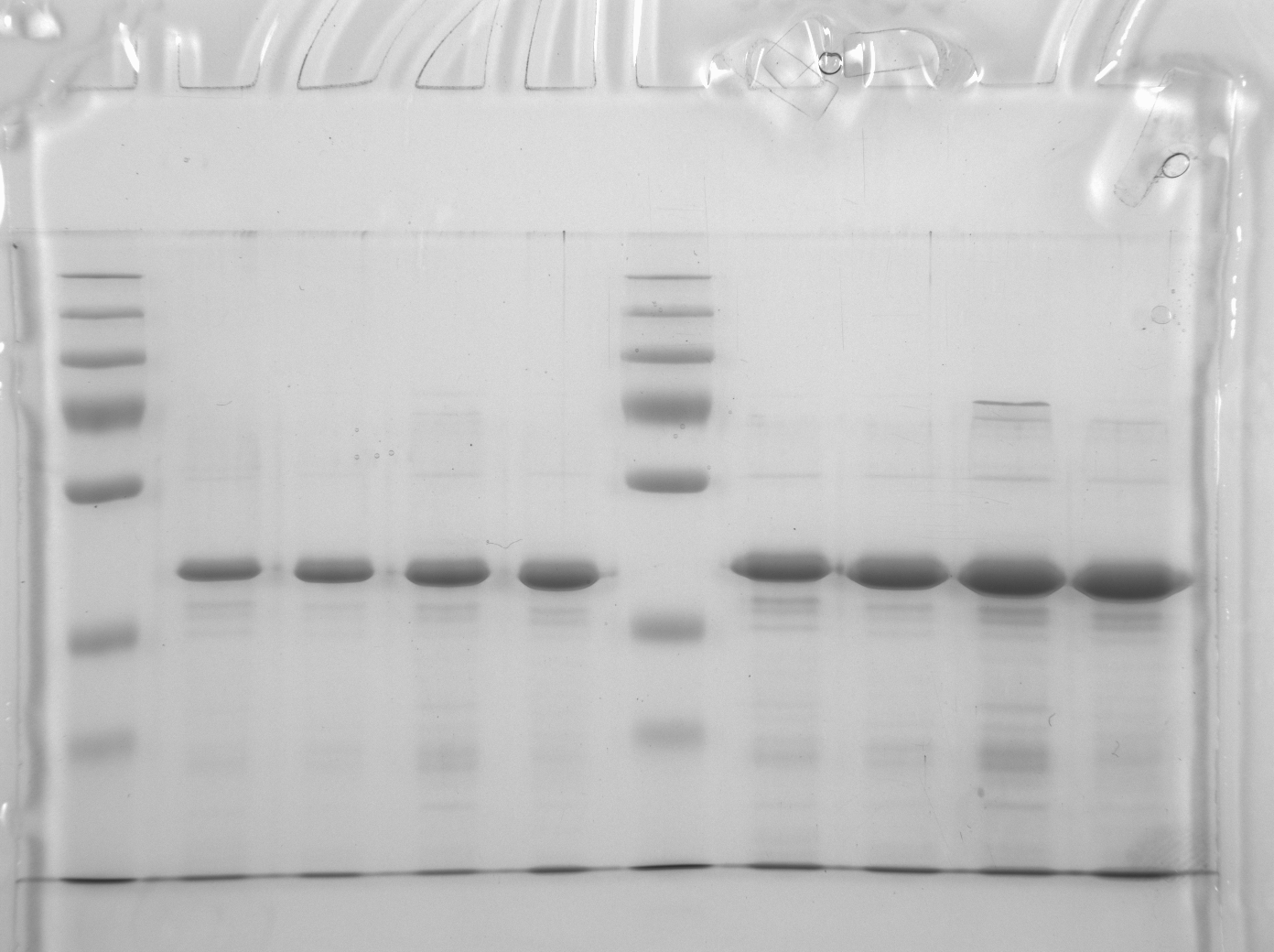

Supplement: Figure 4—source data 2. [file elife-75010-fig4-data2.zip › Figure 4 - source data 2/Fig. 4B.tif]

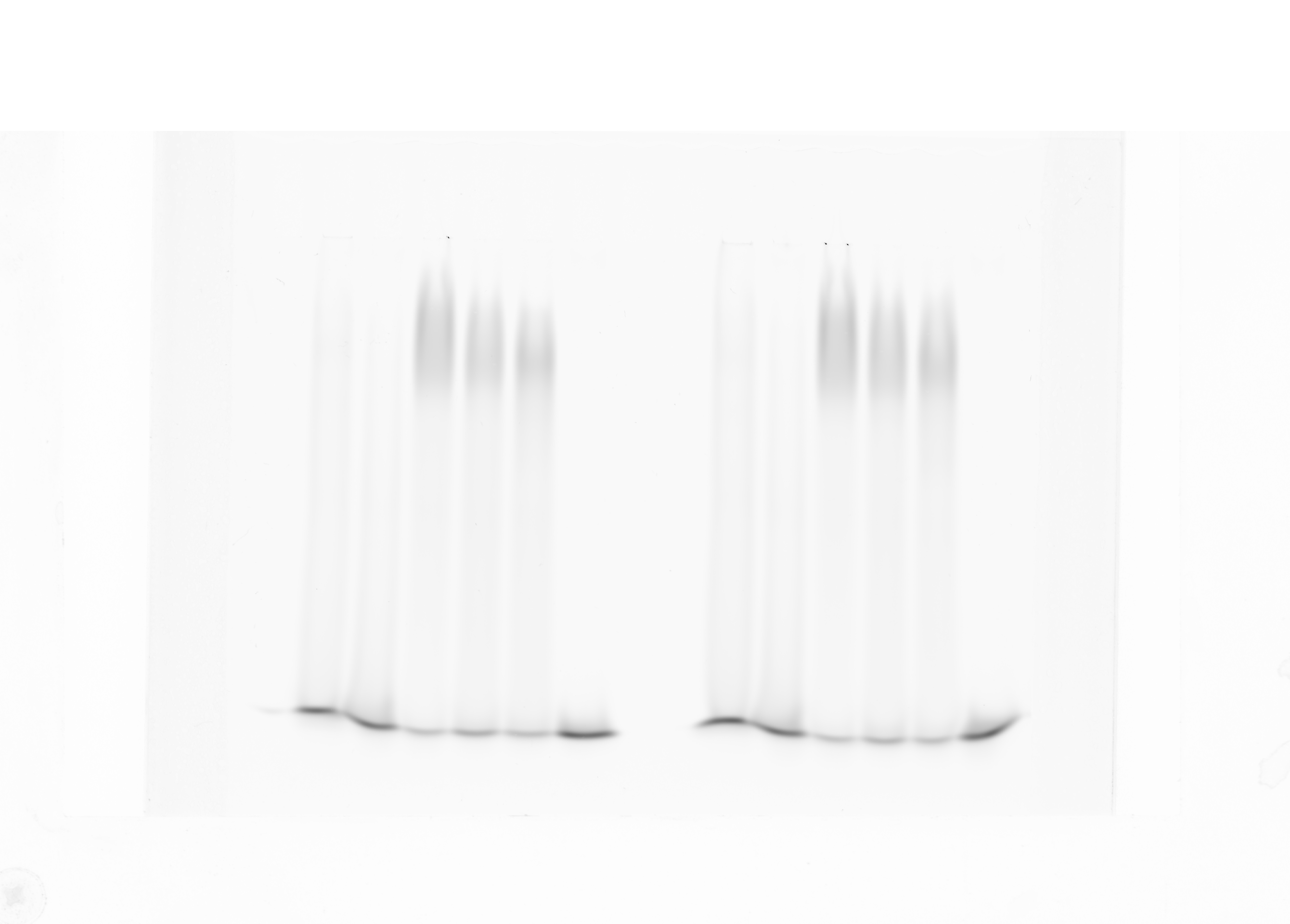

Supplement: Figure 4—source data 2. [file elife-75010-fig4-data2.zip › Figure 4 - source data 2/Fig. 4C.tif]

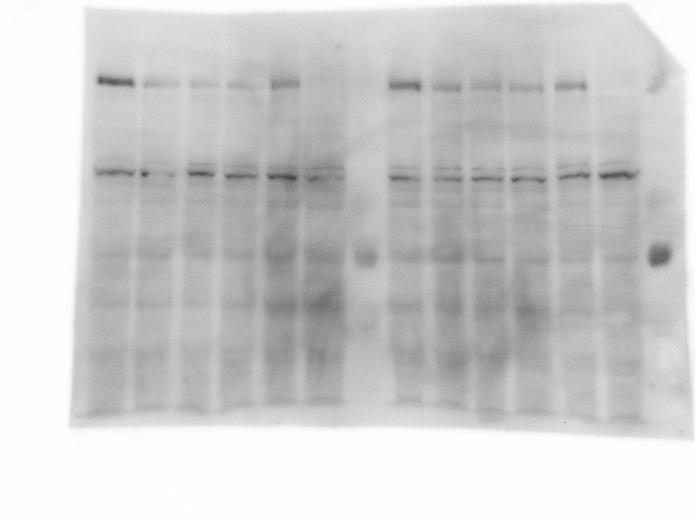

Supplement: Figure 4—source data 2. [file elife-75010-fig4-data2.zip › Figure 4 - source data 2/Fig. 4E blot.tif]

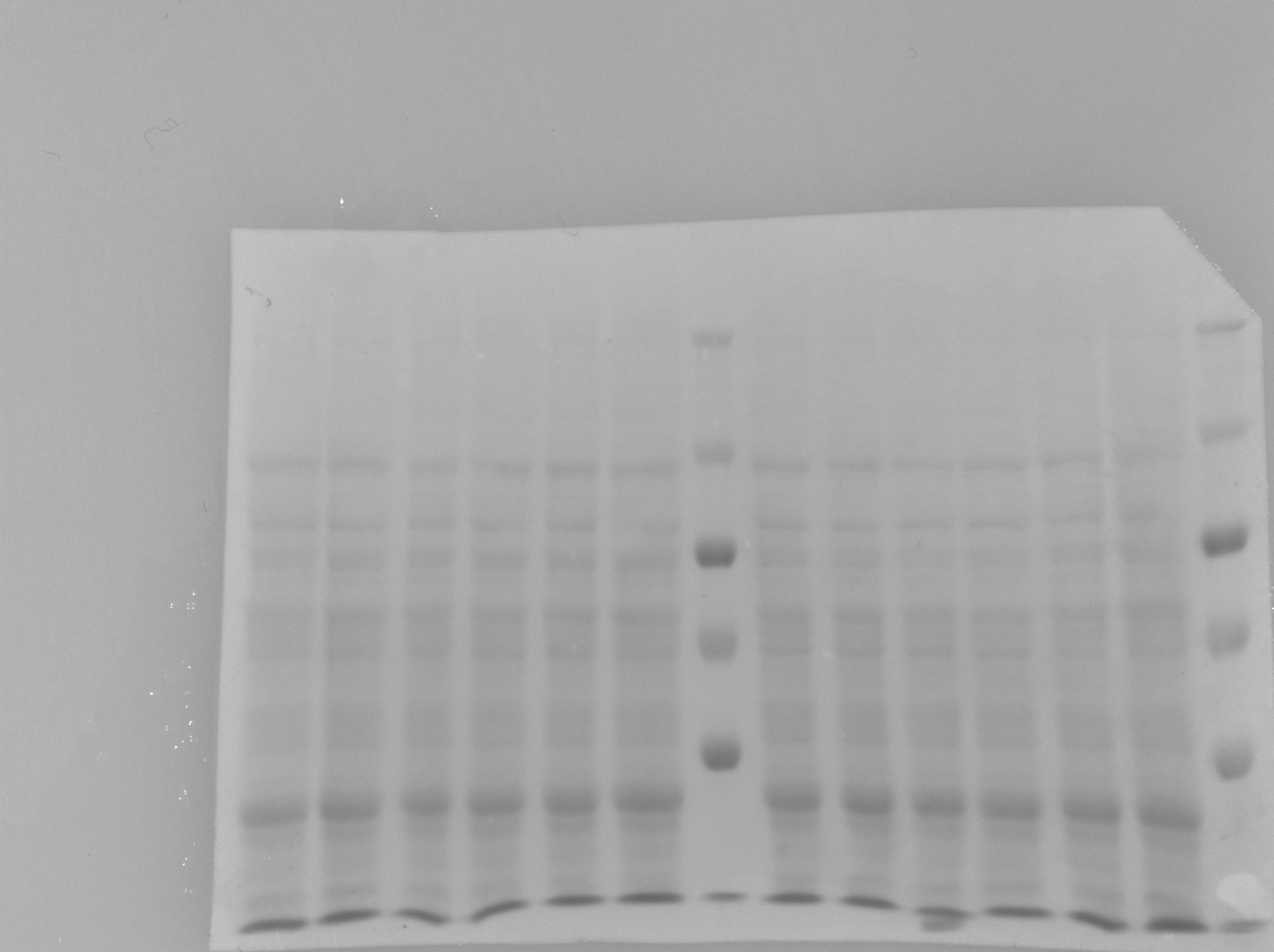

Supplement: Figure 4—source data 2. [file elife-75010-fig4-data2.zip › Figure 4 - source data 2/Fig. 4E ponceau.tif]

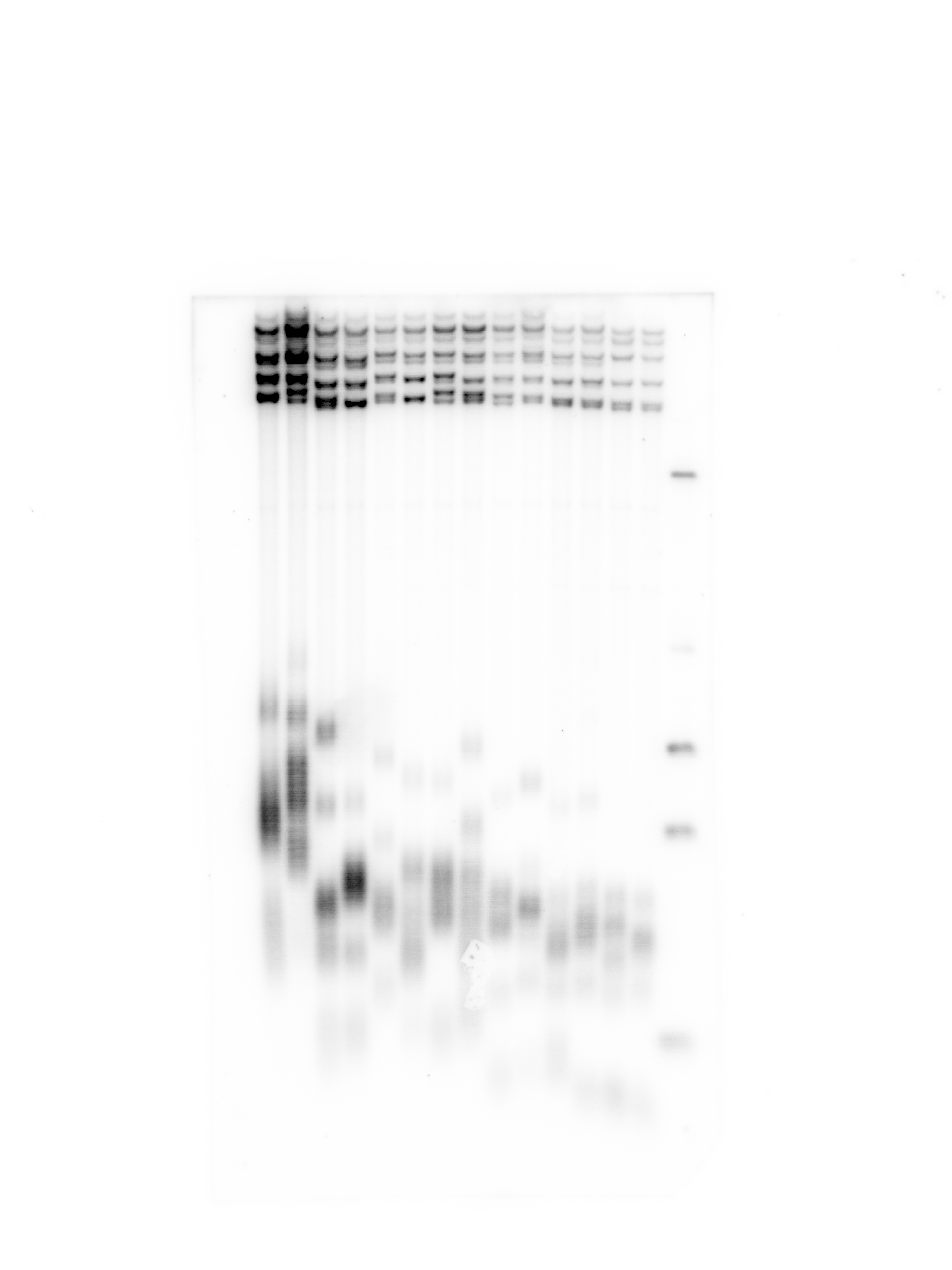

Supplement: Figure 4—source data 2. [file elife-75010-fig4-data2.zip › Figure 4 - source data 2/Fig. 4F.tif]

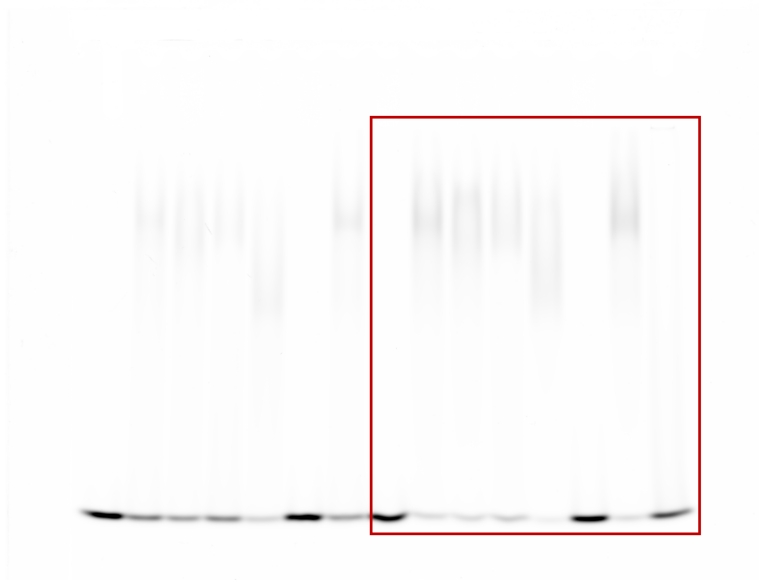

Red square marks the area shown in Figure 4 - figure supplement 1.

Supplement: Figure 4—figure supplement 1—source data 1. [file elife-75010-fig4-figsupp1-data1.zip › Figure 4 - figure supplement 1 - source data 1/Fig. 4 - suppl. 1 labels.pdf]

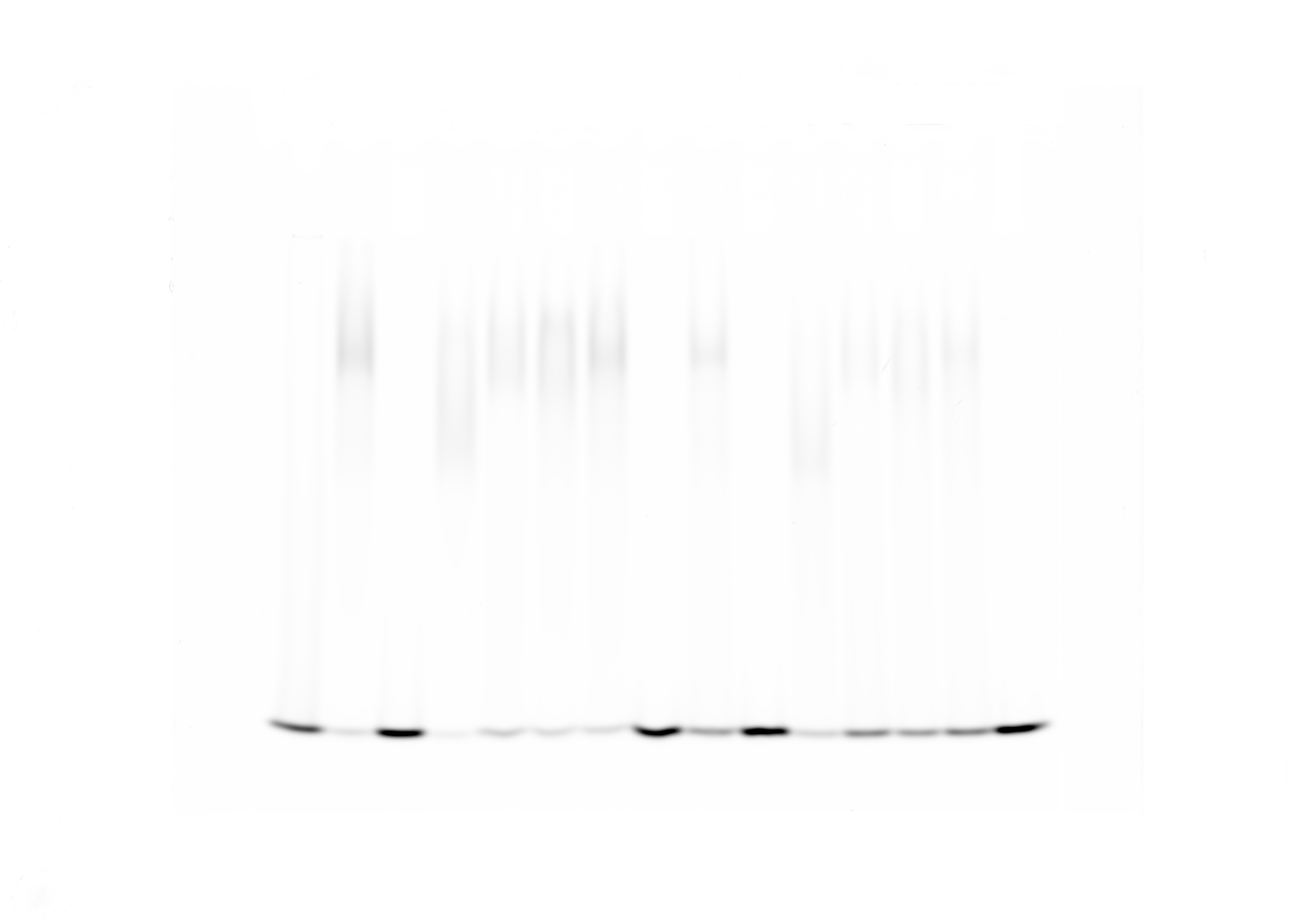

Supplement: Figure 4—figure supplement 1—source data 1. [file elife-75010-fig4-figsupp1-data1.zip › Figure 4 - figure supplement 1 - source data 1/Fig. 4 - suppl. 1.tif]

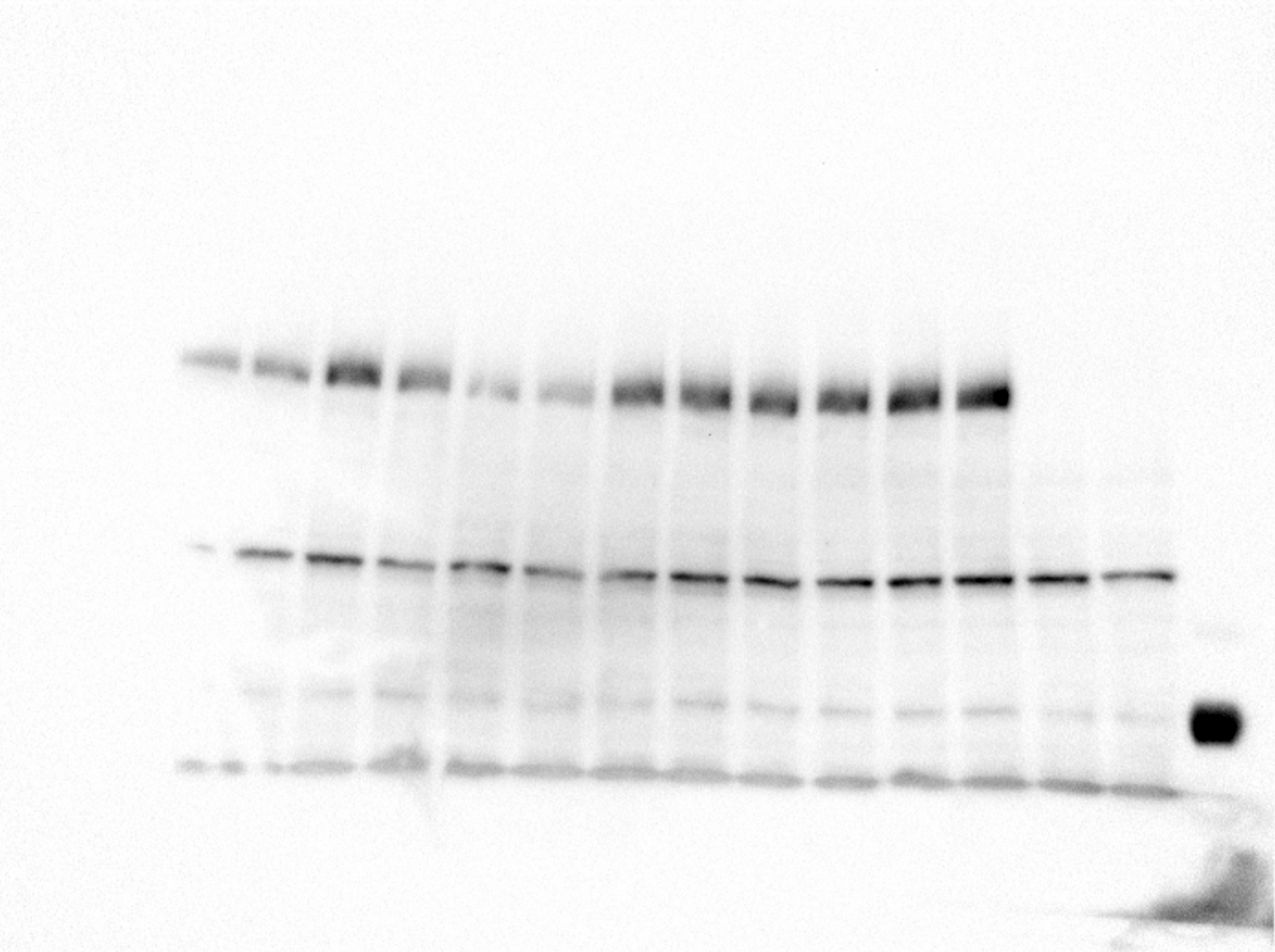

Supplement: Figure 4—figure supplement 2—source data 2. [file elife-75010-fig4-figsupp2-data2.zip › Figure 4 - figure supplement 2 - source data 2/Fig. 4 - suppl. 2B (HA blot left top).tif]

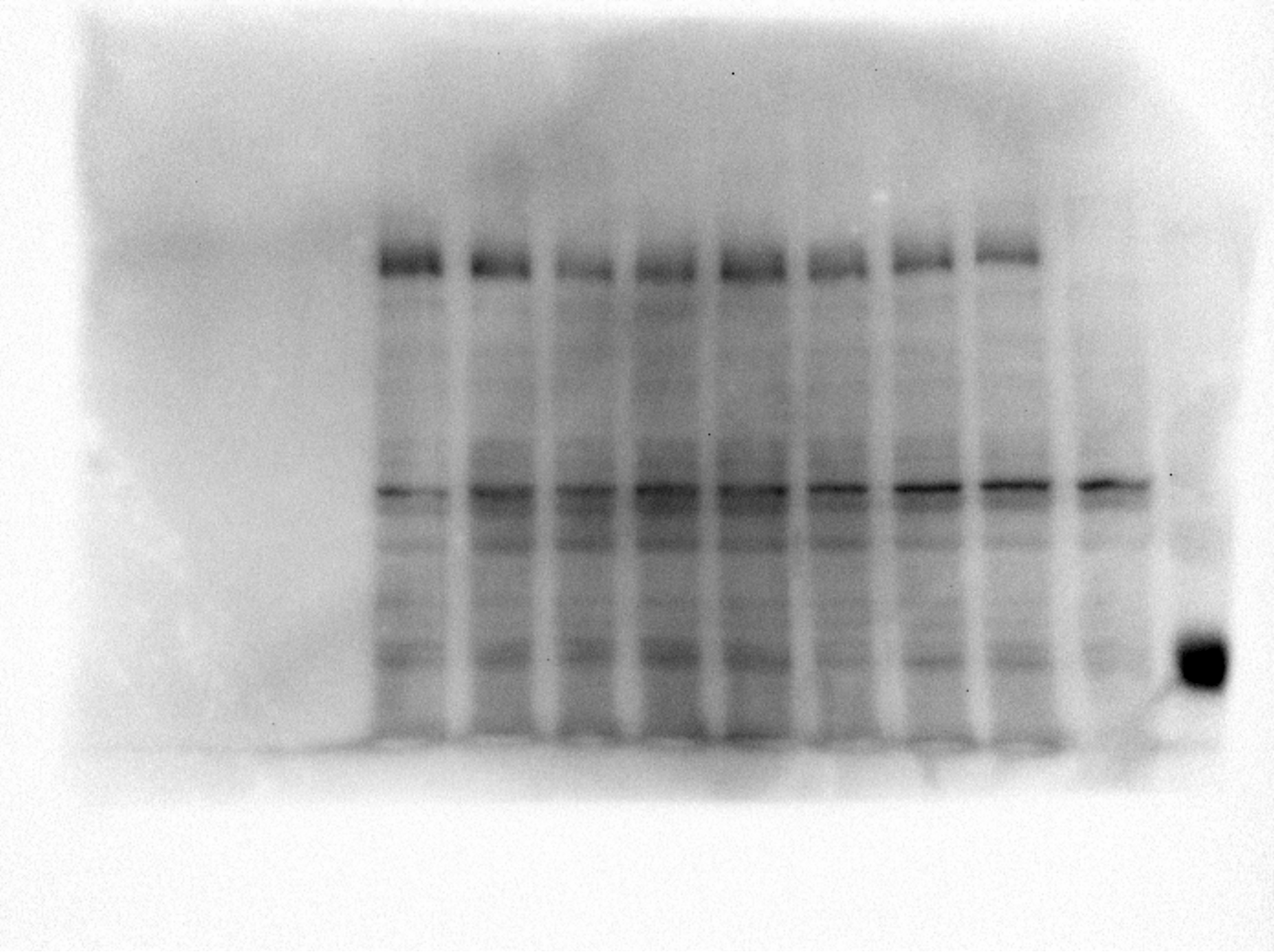

Supplement: Figure 4—figure supplement 2—source data 2. [file elife-75010-fig4-figsupp2-data2.zip › Figure 4 - figure supplement 2 - source data 2/Fig. 4 - suppl. 2B (HA blot right top).tif]

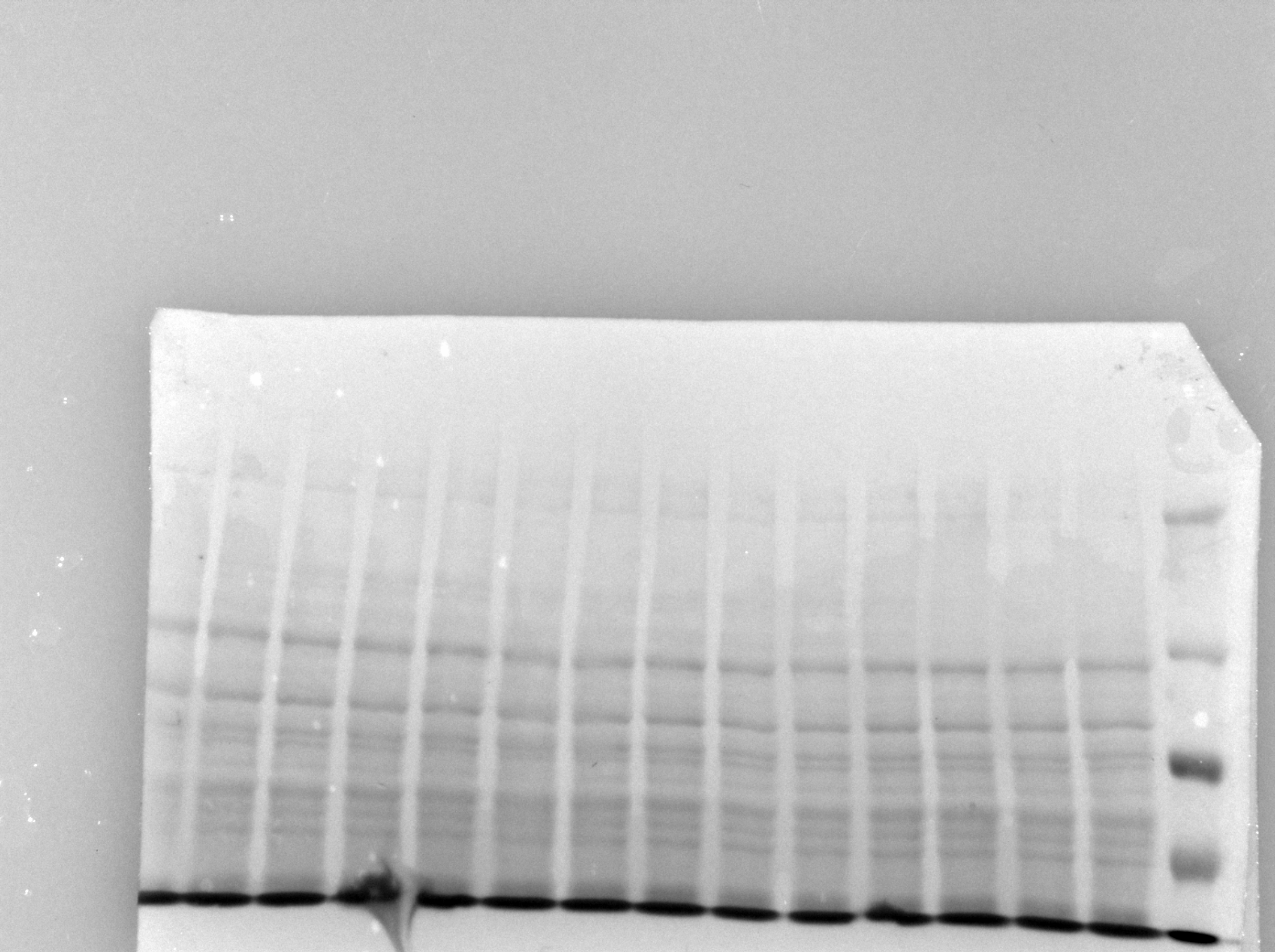

Supplement: Figure 4—figure supplement 2—source data 2. [file elife-75010-fig4-figsupp2-data2.zip › Figure 4 - figure supplement 2 - source data 2/Fig. 4 - suppl. 2B (ponceau left bottom).tif]

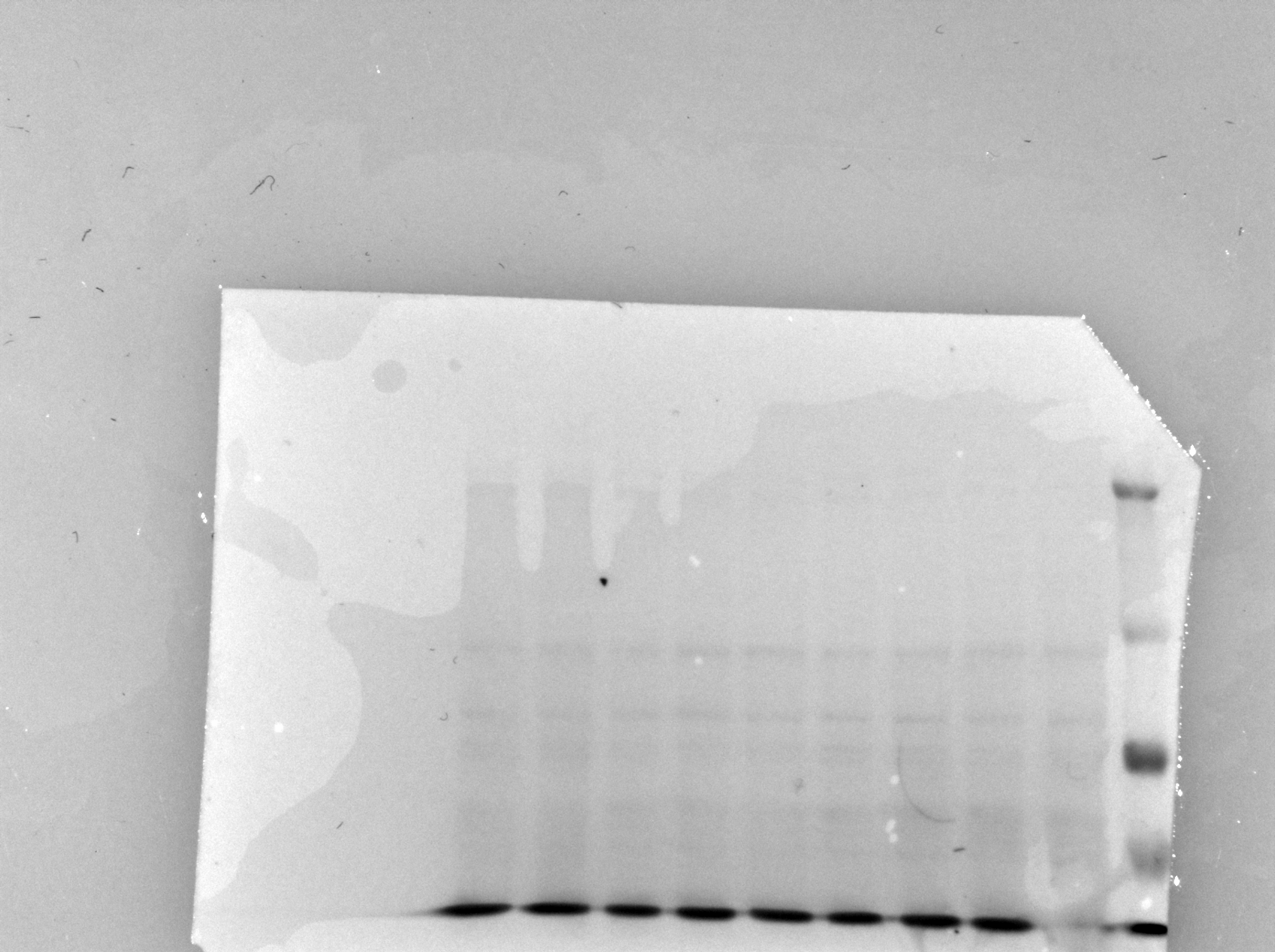

Supplement: Figure 4—figure supplement 2—source data 2. [file elife-75010-fig4-figsupp2-data2.zip › Figure 4 - figure supplement 2 - source data 2/Fig. 4 - suppl. 2B (ponceau right bottom).tif]

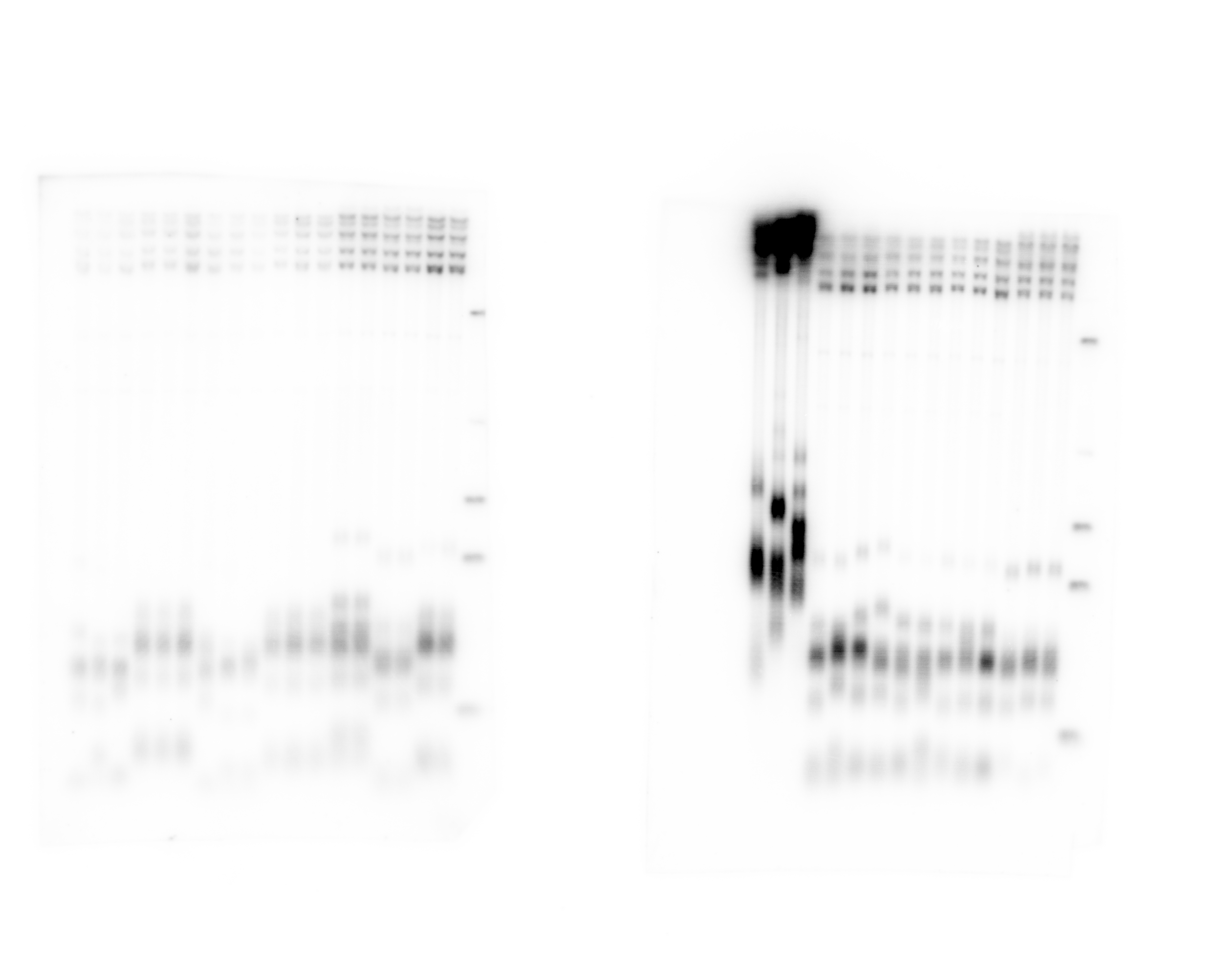

Supplement: Figure 4—figure supplement 2—source data 2. [file elife-75010-fig4-figsupp2-data2.zip › Figure 4 - figure supplement 2 - source data 2/Fig. 4 - suppl. 2D.tif]

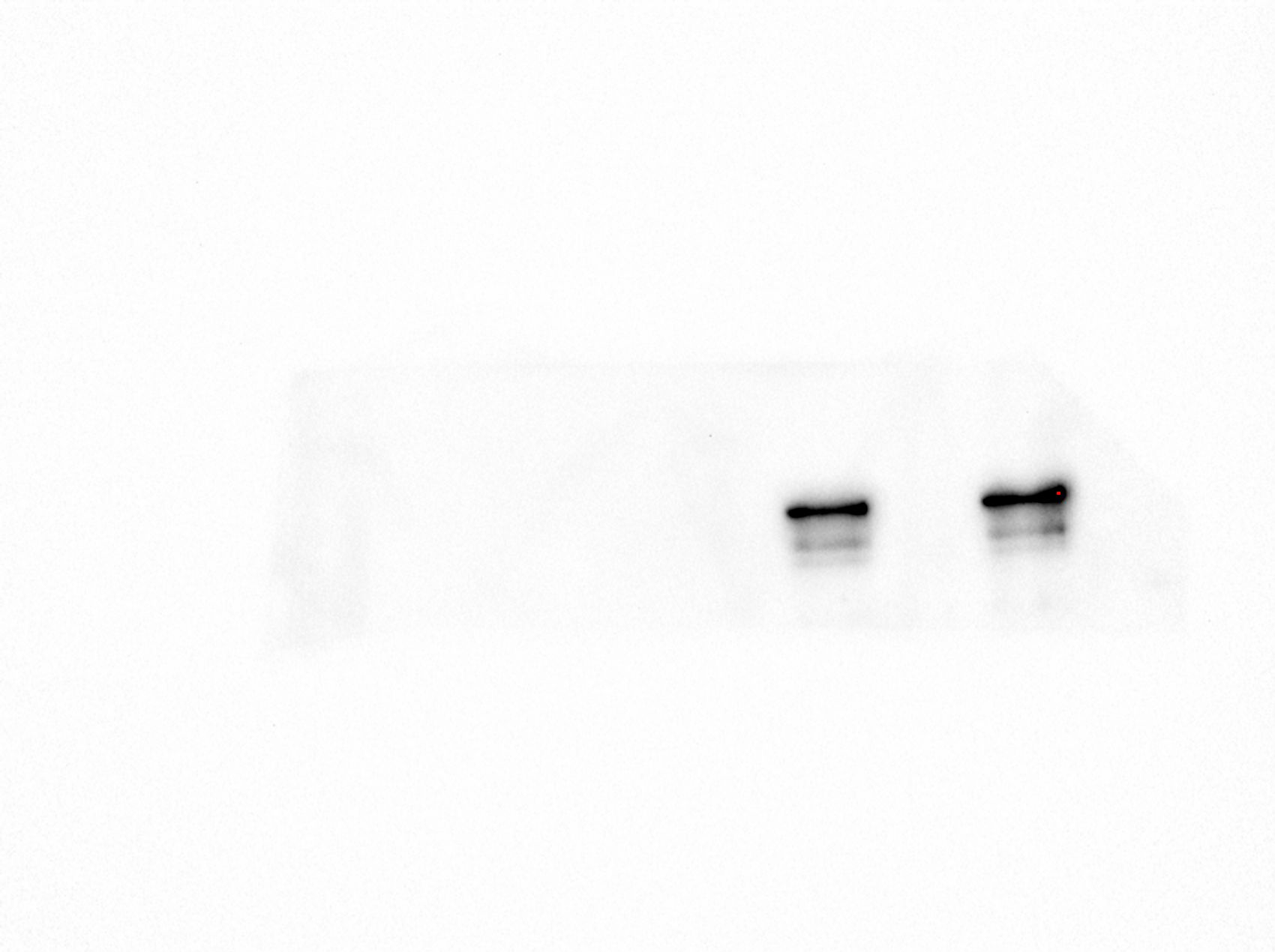

Supplement: Figure 5—source data 2. [file elife-75010-fig5-data2.zip › Figure 5 - source data 2/Fig. 5D (Flag).tif]

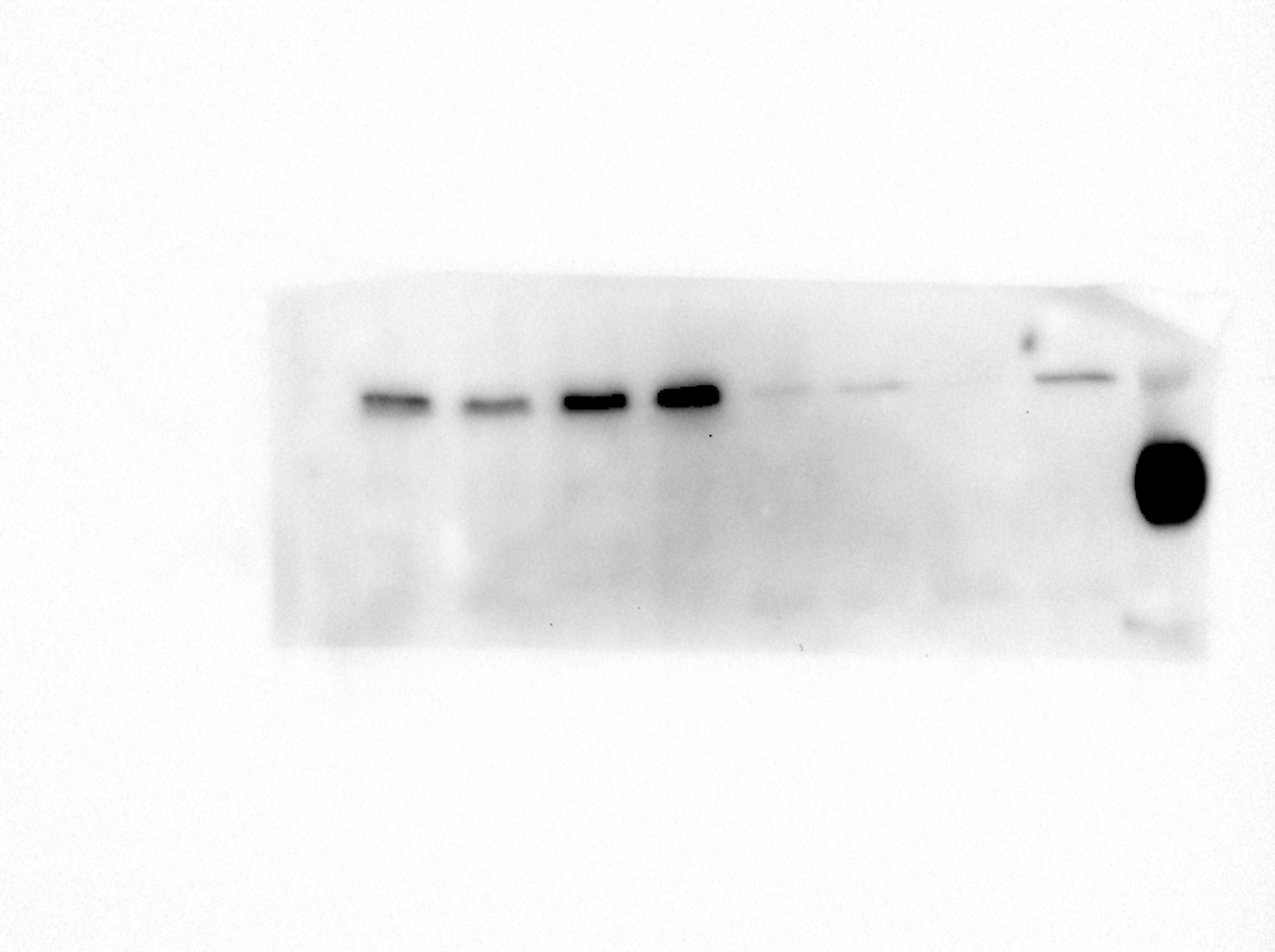

Supplement: Figure 5—source data 2. [file elife-75010-fig5-data2.zip › Figure 5 - source data 2/Fig. 5D (HA).tif]

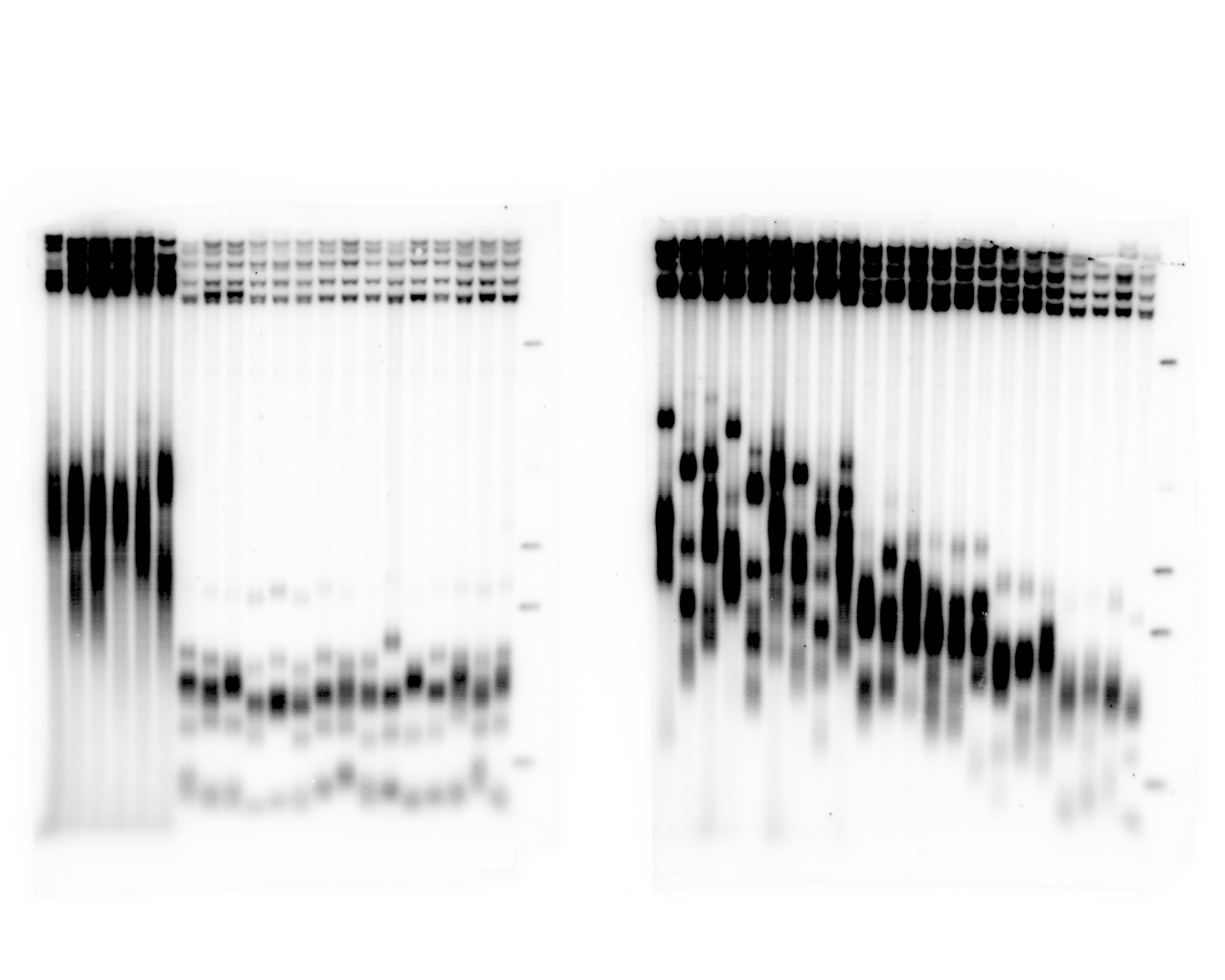

Supplement: Figure 5—source data 2. [file elife-75010-fig5-data2.zip › Figure 5 - source data 2/Fig. 5I.tif]
