# Supplementary material for: Telomere length regulation by Rif1 protein from Hansenula polymorpha
Source: eLife. 2022 Feb 7;11:e75010. doi: 10.7554/eLife.75010 (PMC8820739; doi:10.7554/eLife.75010)
Supplement: Figure 3—figure supplement 1—source data 1. [file elife-75010-fig3-figsupp1-data1.zip › Figure 3 - figure supplement 1 - source data 1/Fig. 3 - suppl. 1 labels.pdf]

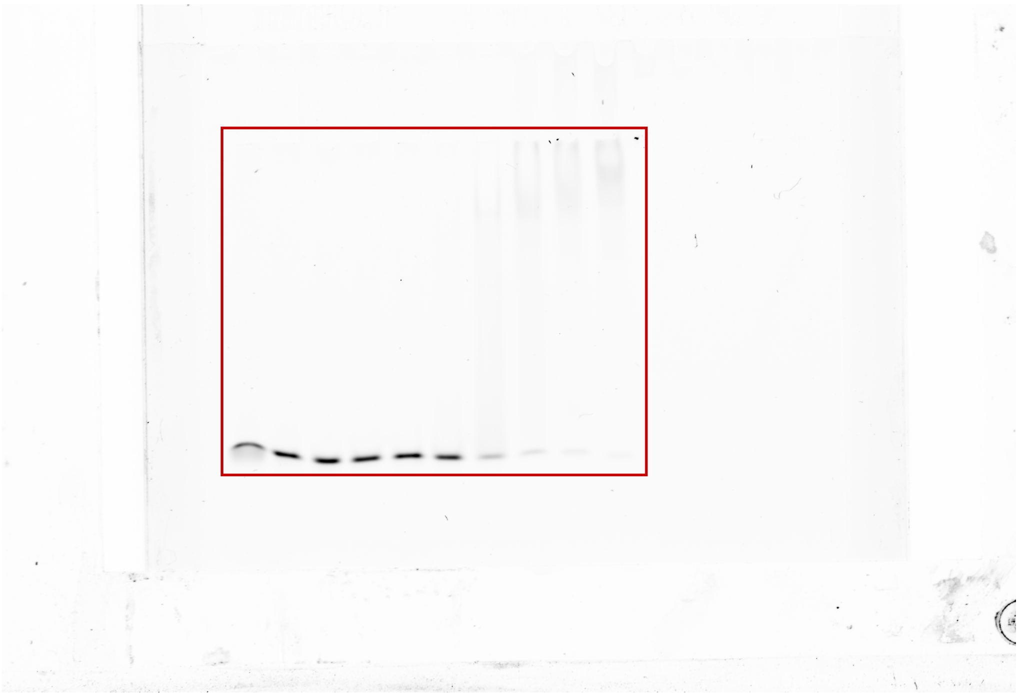

Red square marks the area shown in Figure 3 - figure supplement 1A (Tel4(G)).

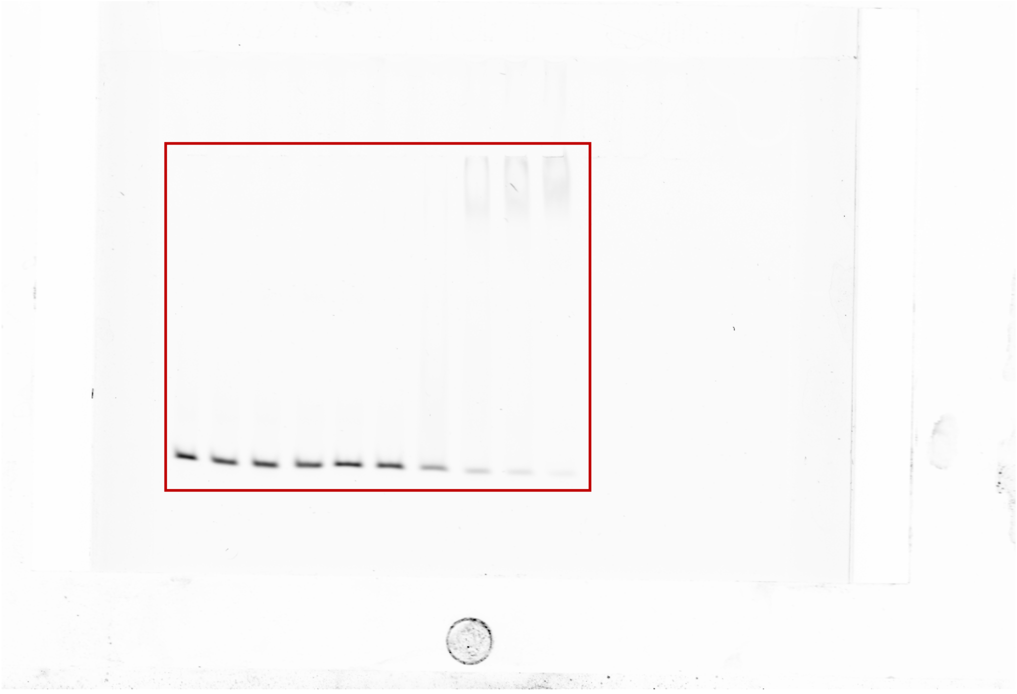

Red square marks the area shown in Figure 3 - figure supplement 1A (Tel4(GC)).

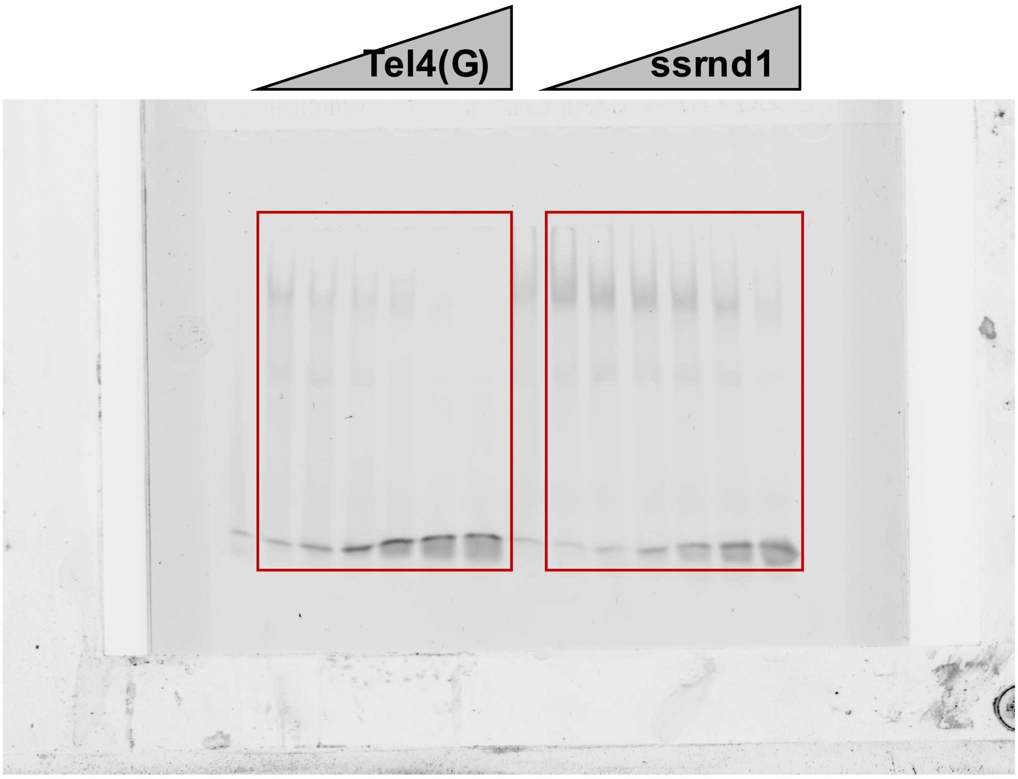

Red square marks the area shown in Figure 3 - figure supplement 1B (Tel4(G) and ssrnd1).

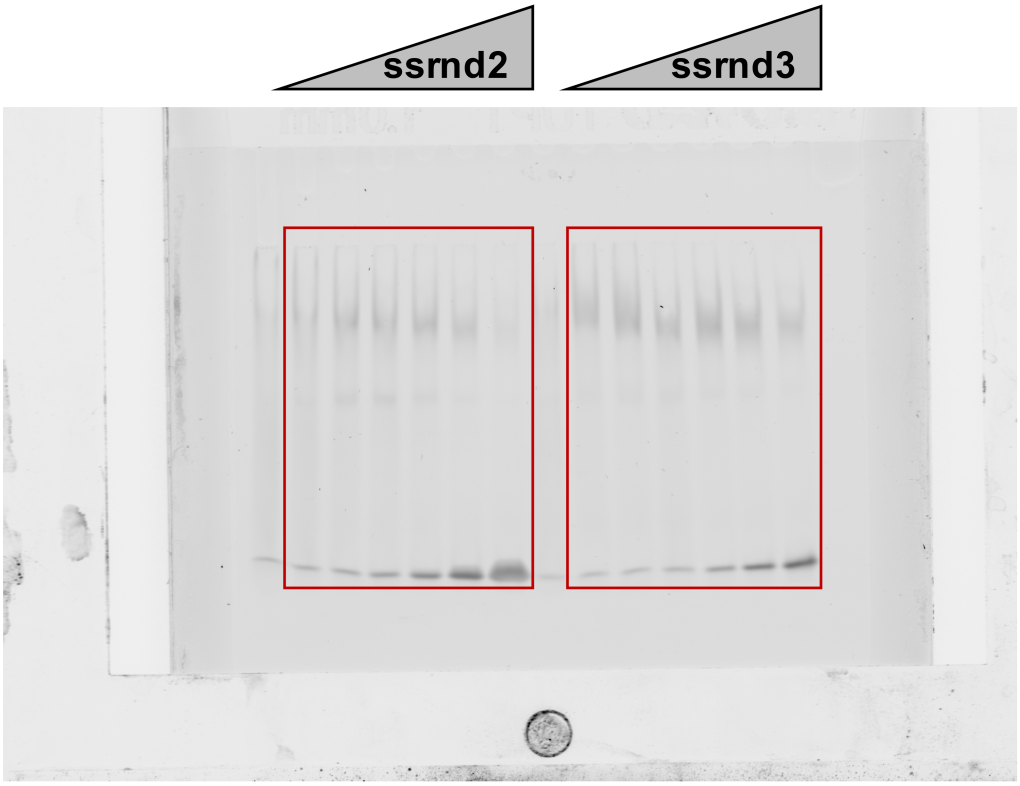

Red square marks the area shown in Figure 3 - figure supplement 1B (ssrnd2 and ssrnd3).

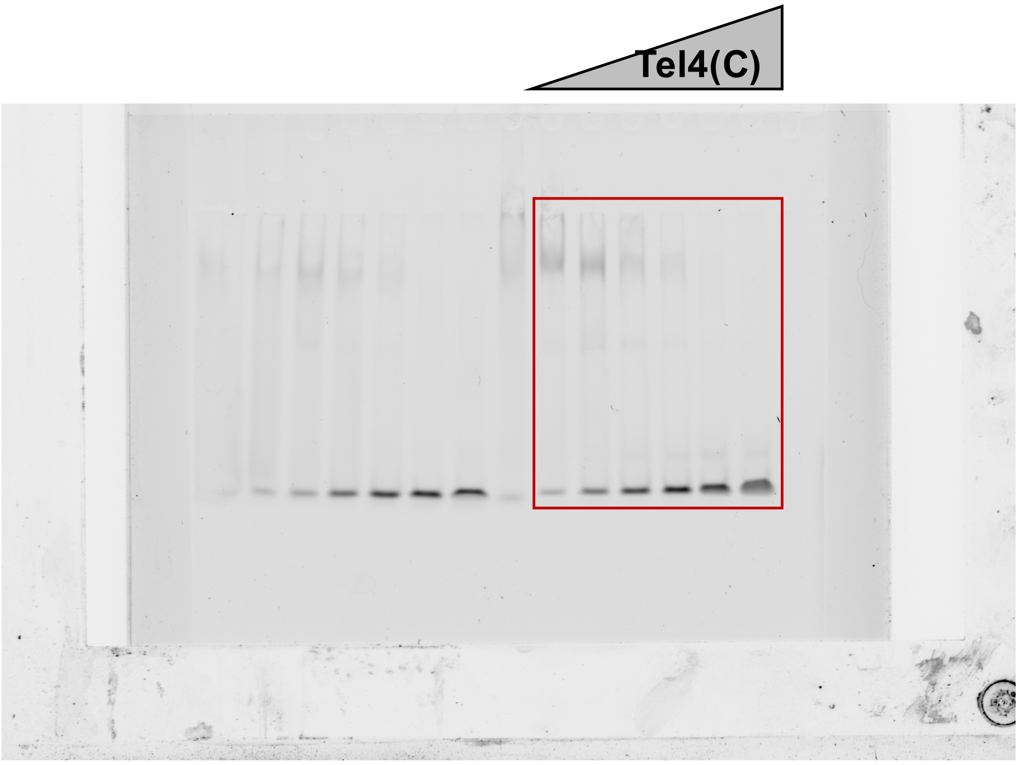

Red square marks the area shown in Figure 3 - figure supplement 1B (Tel4(C)).

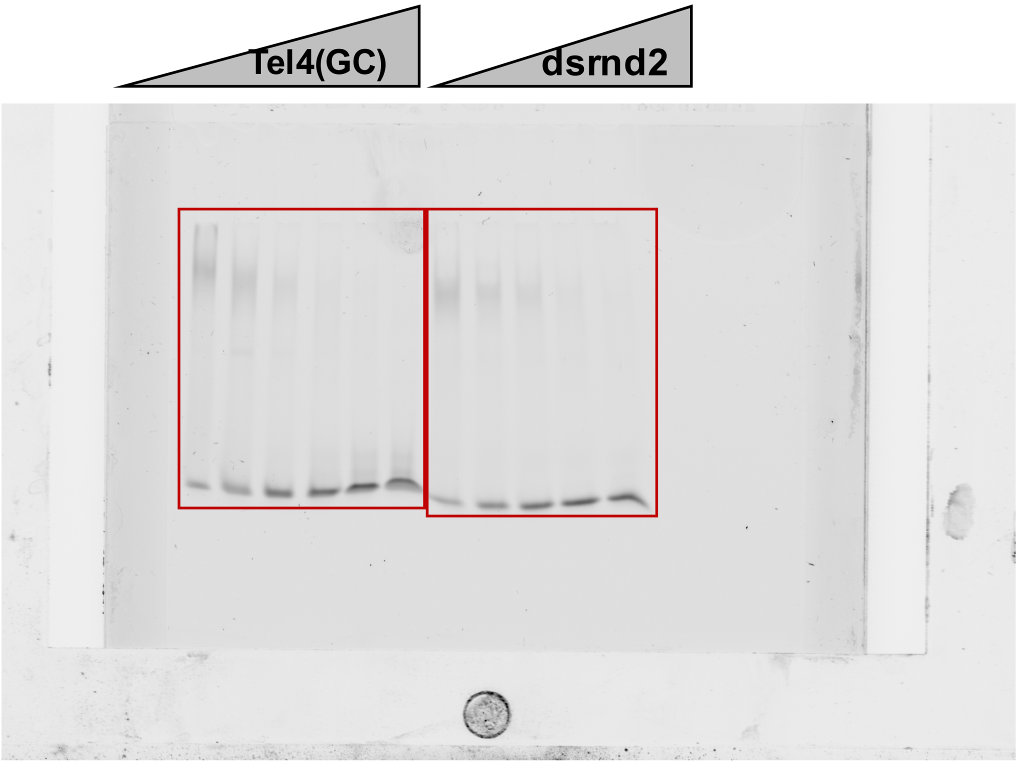

Red square marks the area shown in Figure 3 - figure supplement 1B (Tel4(GC) and dsrnd2).

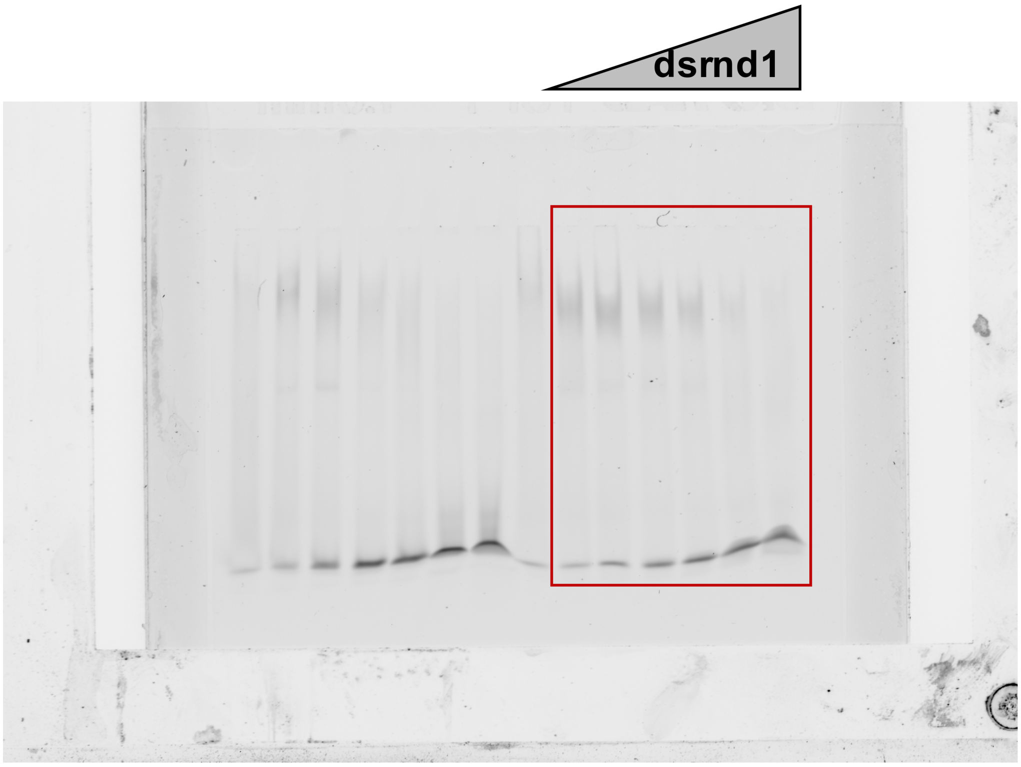

Red square marks the area shown in Figure 3 - figure supplement 1B (dsrnd1).
