# Supplementary material for: Telomere length regulation by Rif1 protein from Hansenula polymorpha
Source: eLife. 2022 Feb 7;11:e75010. doi: 10.7554/eLife.75010 (PMC8820739; doi:10.7554/eLife.75010)
Supplement: Figure 4—source data 2. [file elife-75010-fig4-data2.zip › Figure 4 - source data 2/Fig. 4 labels.pdf]

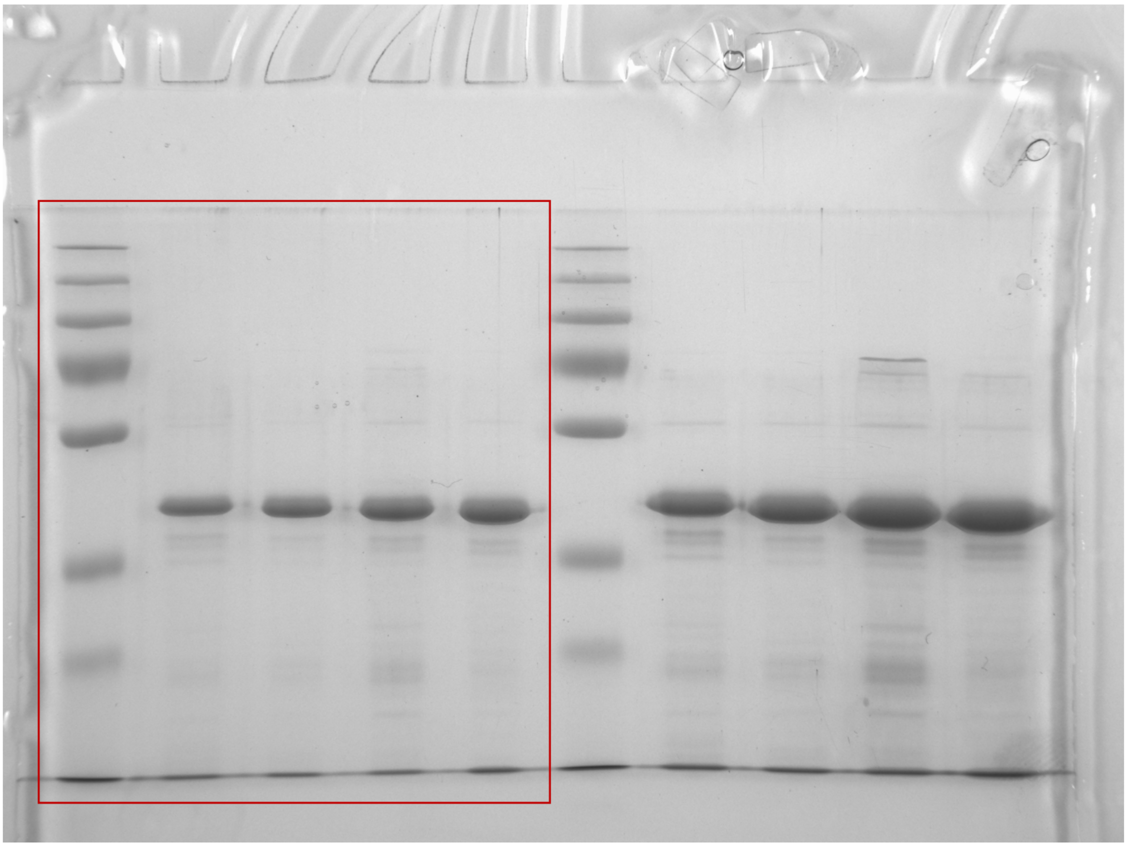

Red square marks the area shown in Figure 4B.

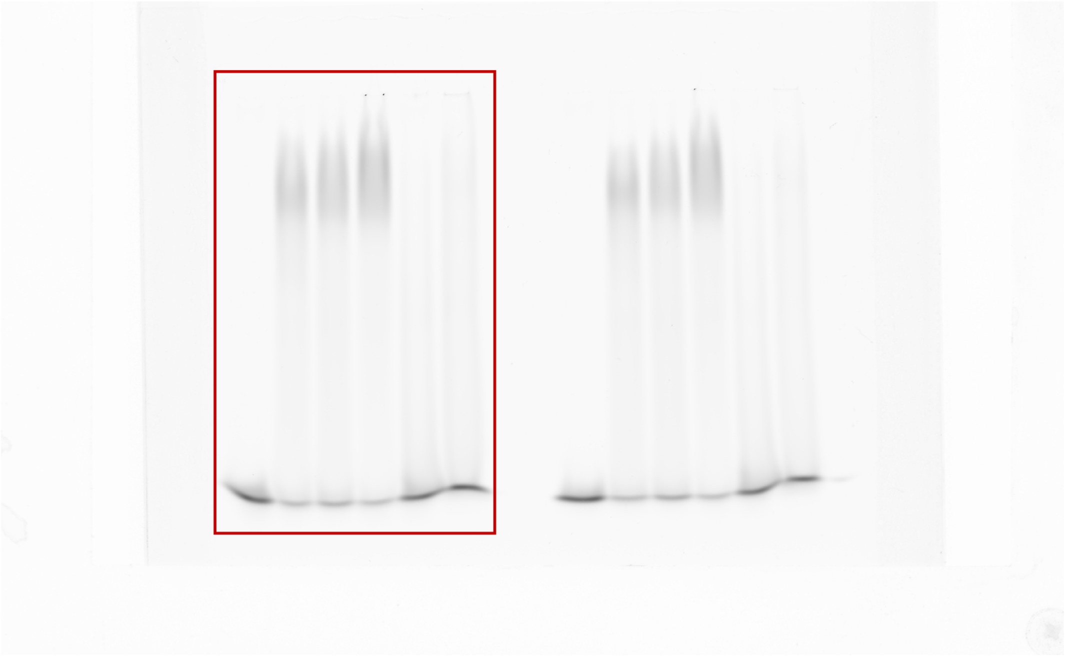

Red square marks the area shown in Figure 4C.

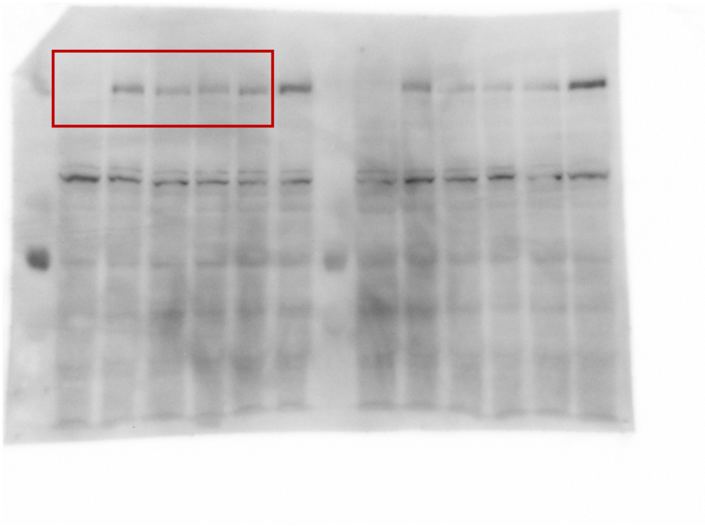

Red square marks the area shown in Figure 4E (**α**-HA blot)

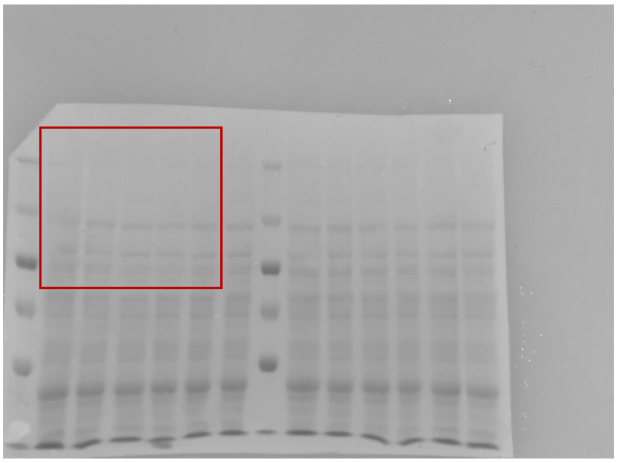

Red square marks the area shown in Figure 4E (Ponceau S)

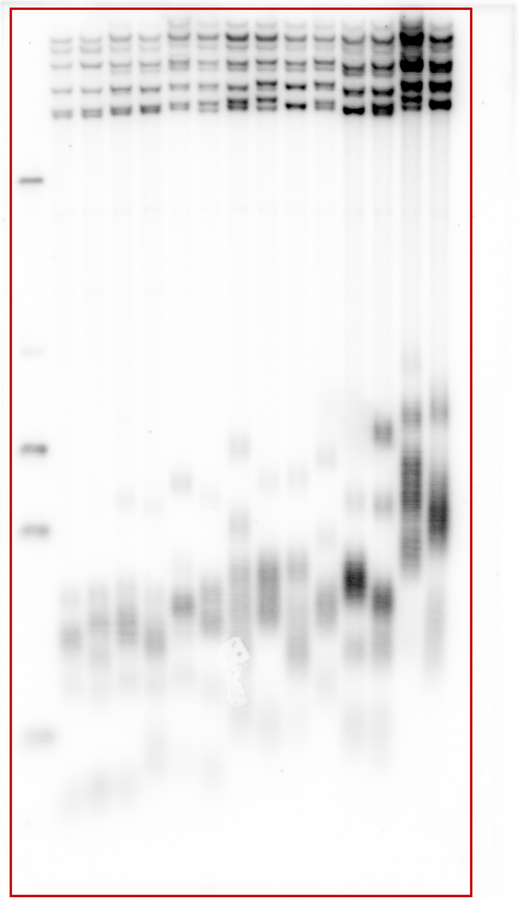

Red square marks the area shown in Figure 4F
