# Supplementary material for: Telomere length regulation by Rif1 protein from Hansenula polymorpha
Source: eLife. 2022 Feb 7;11:e75010. doi: 10.7554/eLife.75010 (PMC8820739; doi:10.7554/eLife.75010)
Supplement: Figure 4—figure supplement 2—source data 2. [file elife-75010-fig4-figsupp2-data2.zip › Figure 4 - figure supplement 2 - source data 2/Fig. 4 - suppl. 2 labels.pdf]

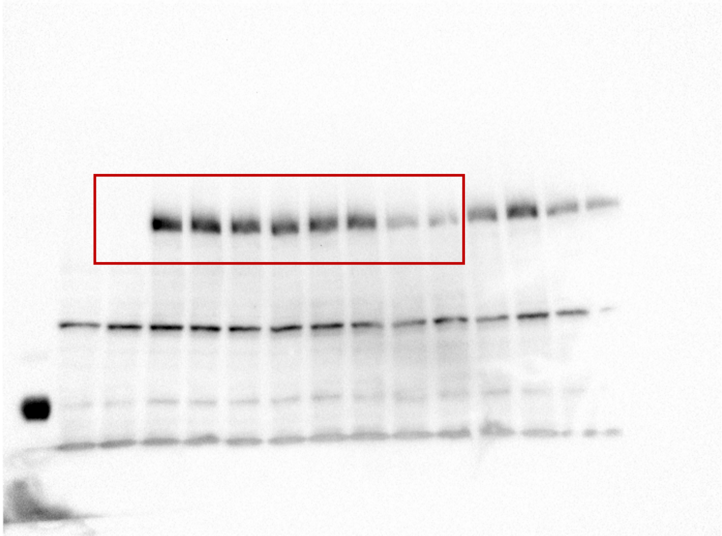

Red square marks the area shown in Figure 4 - figure supplement 2B (α-HA blot top left).

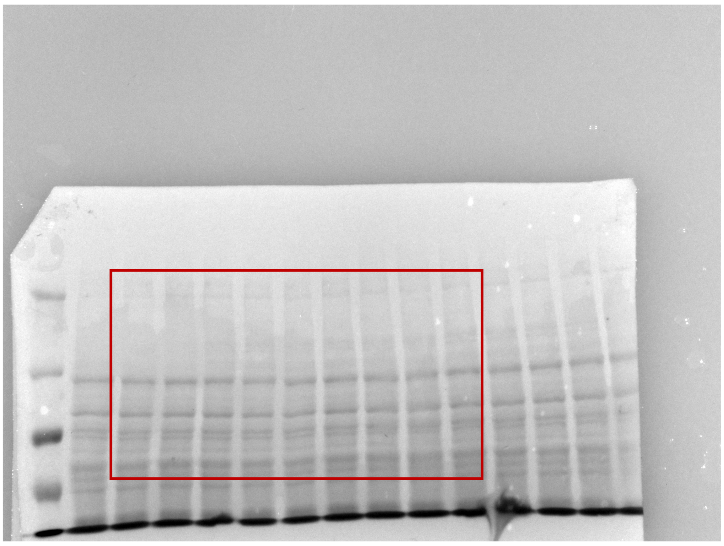

Red square marks the area shown in Figure 4 - figure supplement 2B (ponceau bottom left).

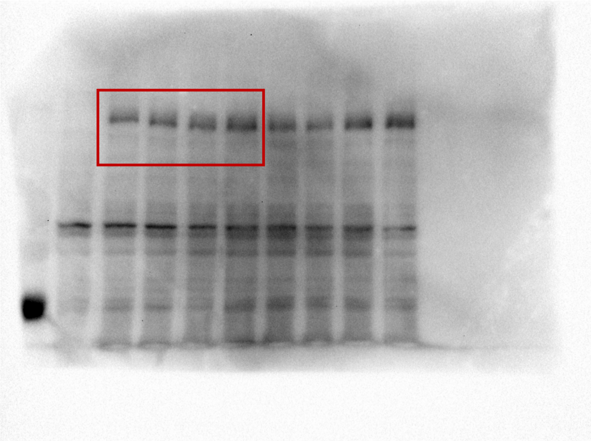

Red square marks the area shown in Figure 4 - figure supplement 2B (**α**-HA blot top right).

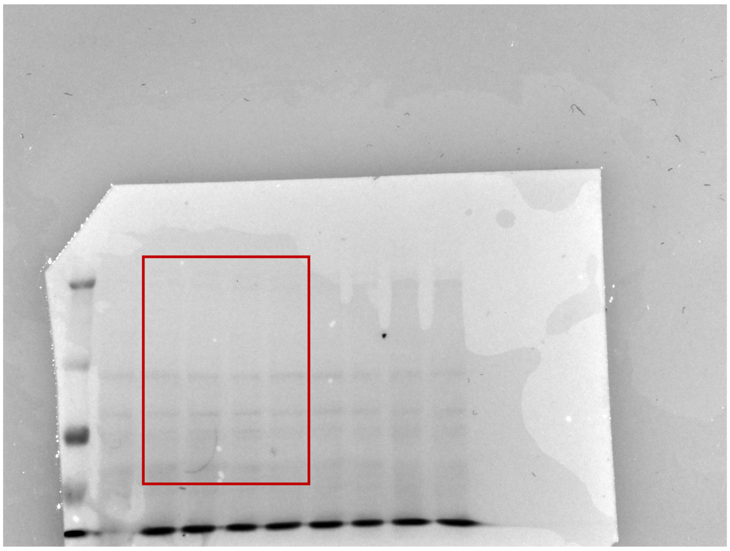

Red square marks the area shown in Figure 4 - figure supplement 2B (ponceau bottom right).

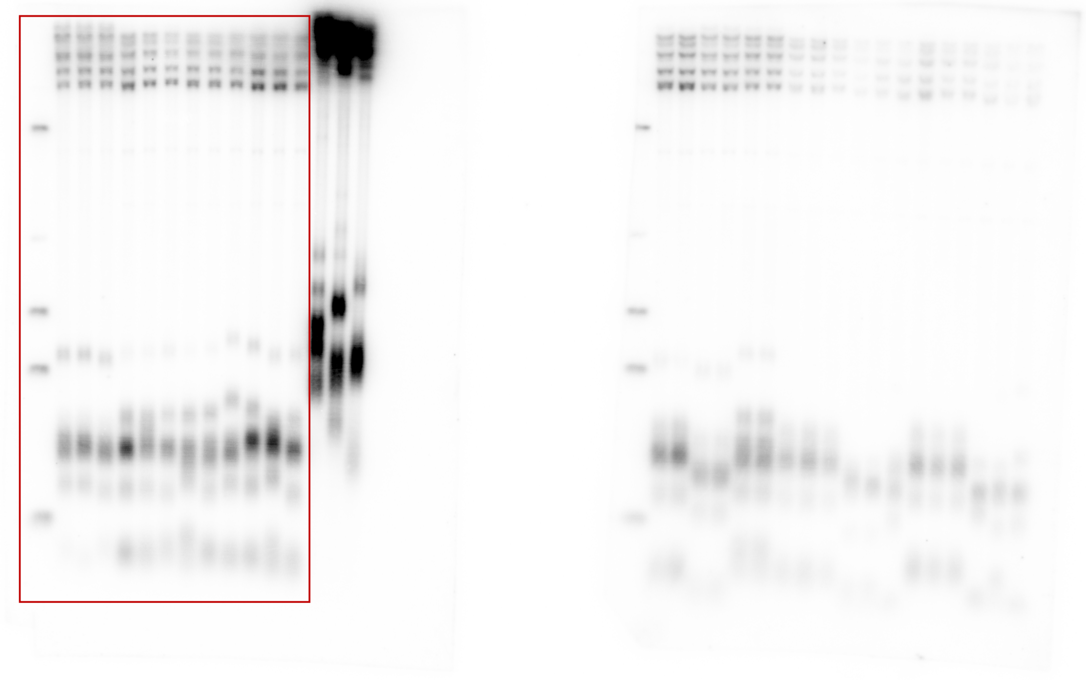

Red square marks the area shown in Figure 4 - figure supplement 2D.
