# Supplementary material for: Telomere length regulation by Rif1 protein from Hansenula polymorpha
Source: eLife. 2022 Feb 7;11:e75010. doi: 10.7554/eLife.75010 (PMC8820739; doi:10.7554/eLife.75010)
Supplement: Figure 5—source data 2. [file elife-75010-fig5-data2.zip › Figure 5 - source data 2/Fig. 5 labels.pdf]

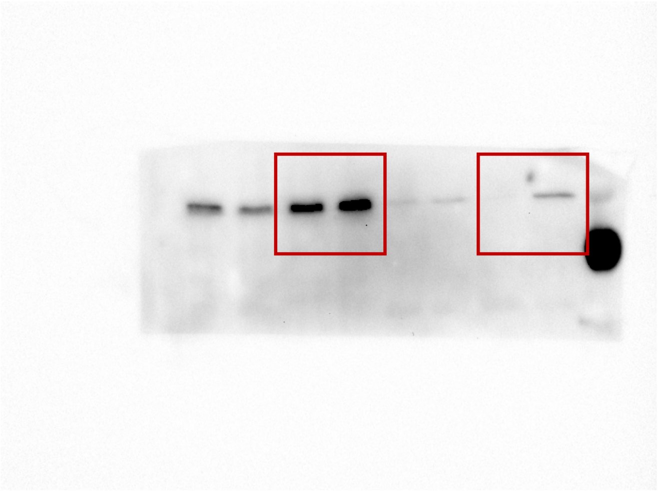

Red square marks the area shown in Figure 5D (**α**-HA blot)

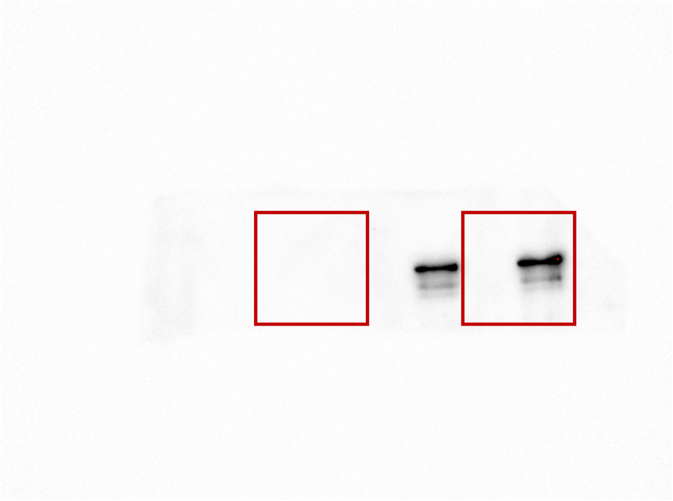

Red square marks the area shown in Figure 5D (**α**-Flag blot)

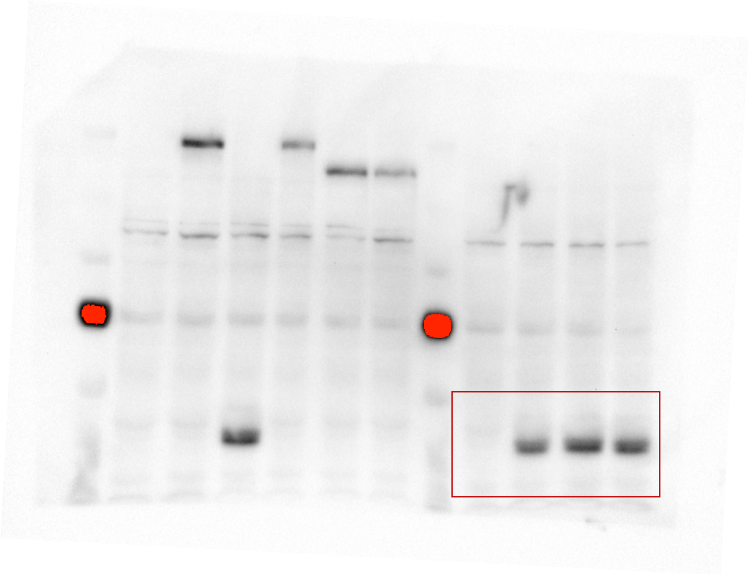

Red square marks the area shown in Figure 5G (**α**-HA blot)

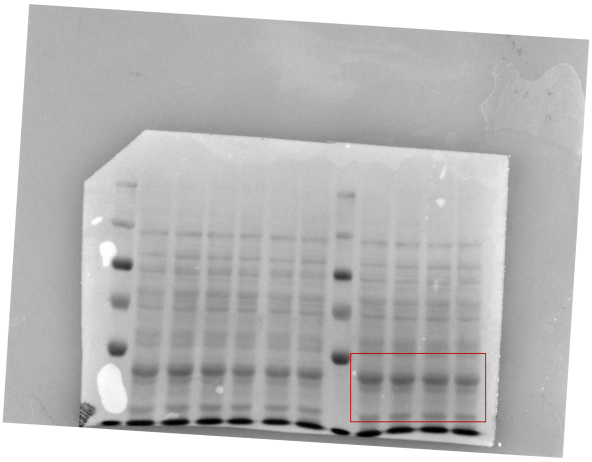

Red square marks the area shown in Figure 5G (Ponceau)

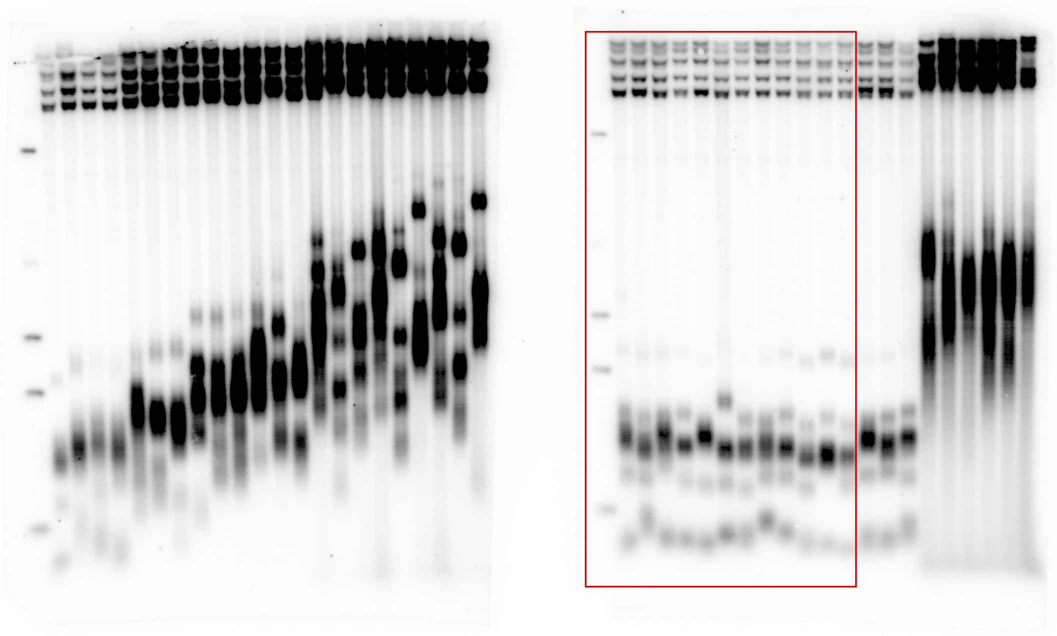

Red square marks the area shown in Figure 5I

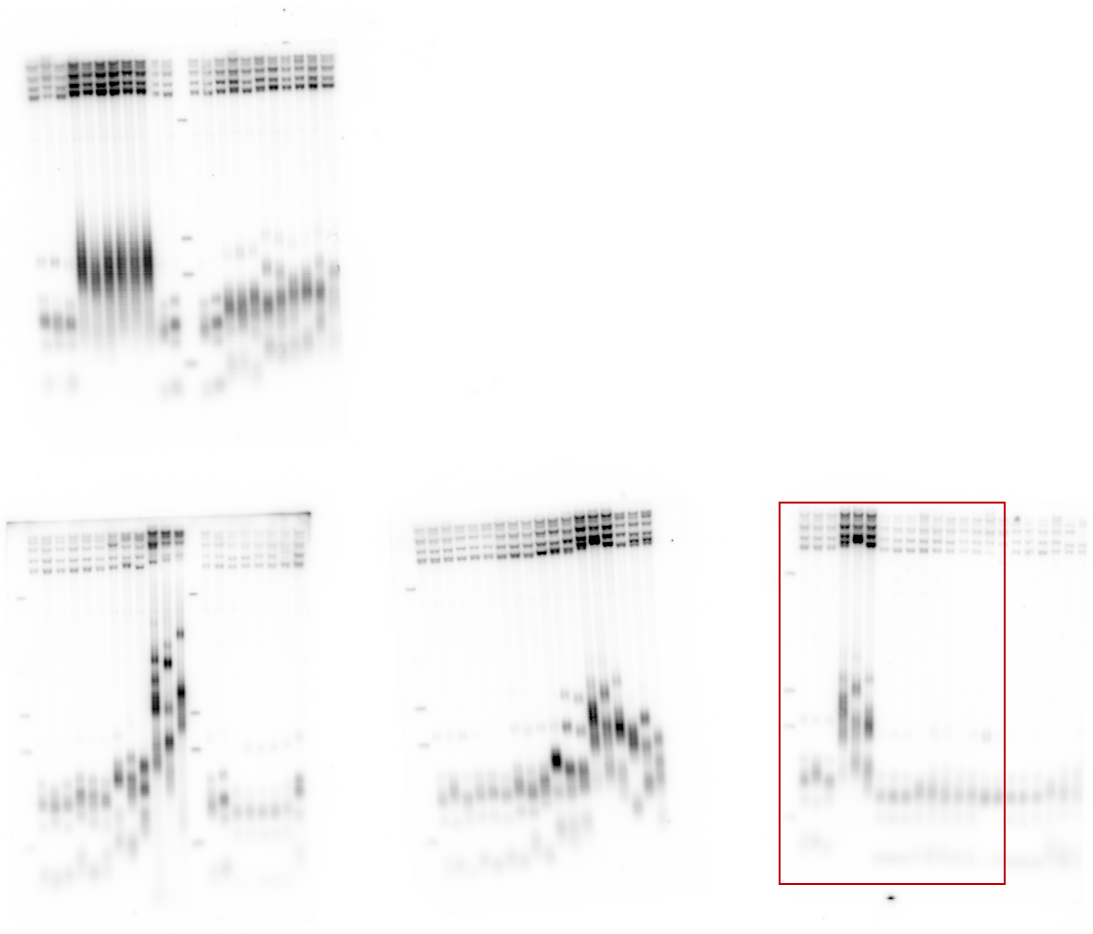

Red square marks the area shown in Figure 5J (the raw unedited blot is in the Figure 5 - source data 3 folder)
