# Supplementary material for: Telomere length regulation by Rif1 protein from Hansenula polymorpha
Source: eLife. 2022 Feb 7;11:e75010. doi: 10.7554/eLife.75010 (PMC8820739; doi:10.7554/eLife.75010)
Supplement: Figure 5—figure supplement 2—source data 2. [file elife-75010-fig5-figsupp2-data2.zip › Figure 5 - figure supplement 2 - source data 2/Fig. 5 - suppl. 2 labels.pdf]

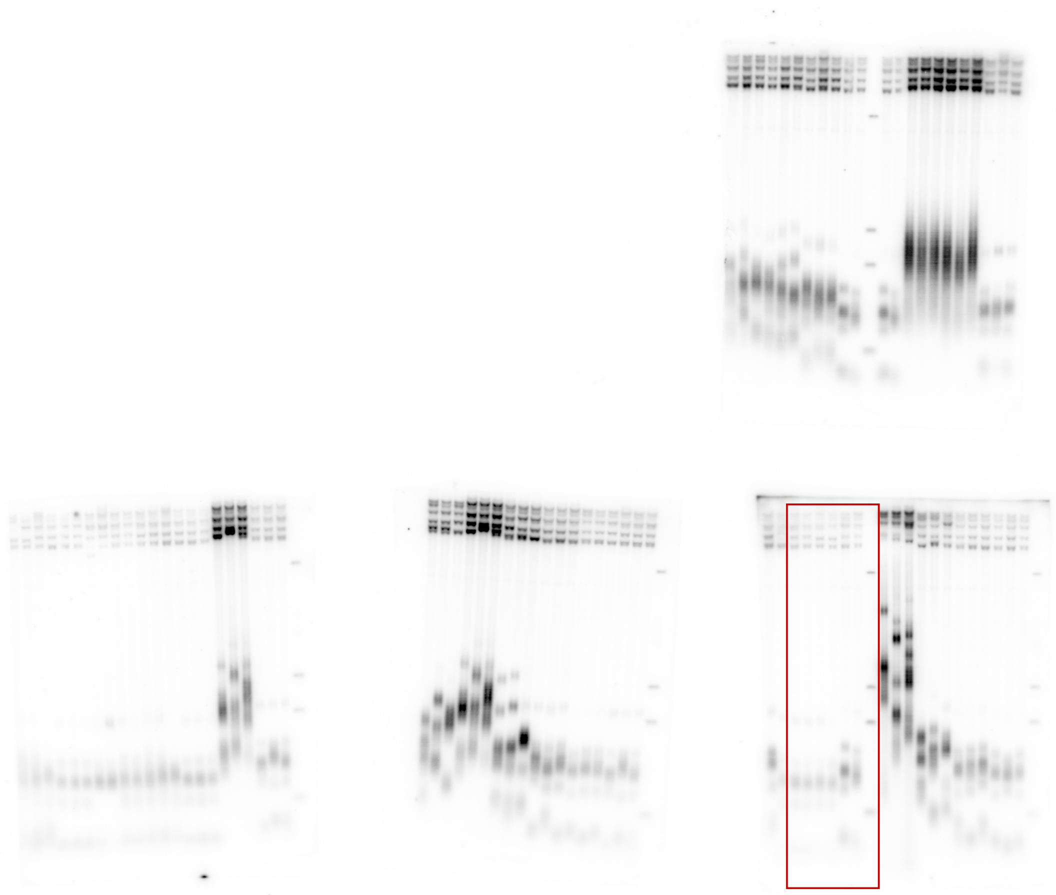

Red square marks the area shown in Figure 5 - figure supplement 2B.

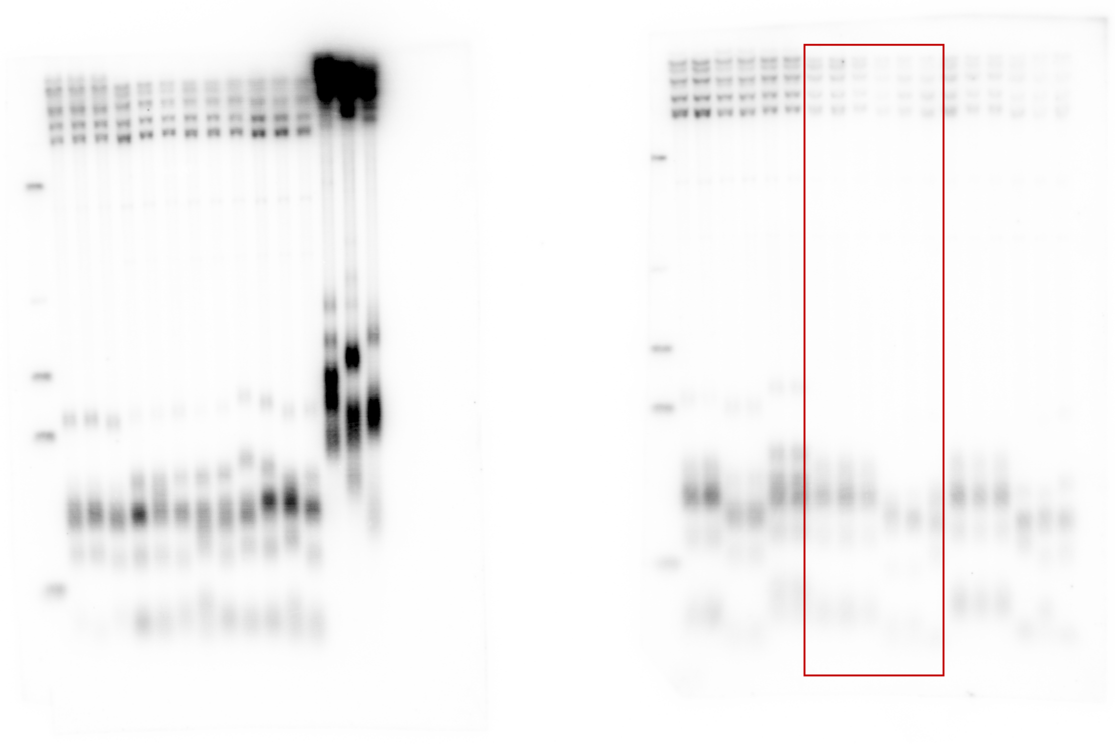

Red square marks the area shown in Figure 5 - figure supplement 2E (the raw unedited blot is in the Figure 5 - figure supplement 2 - source data 3 folder).
