# Supplementary material for: Telomere length regulation by Rif1 protein from Hansenula polymorpha
Source: eLife. 2022 Feb 7;11:e75010. doi: 10.7554/eLife.75010 (PMC8820739; doi:10.7554/eLife.75010)
Supplement: Supplementary file 1. [file elife-75010-supp1.docx]

HANPO 1 MSANDNDTG--RTSPTVIVAPTT--------------------------------------------------------S
SACCE 1 MSKDFSDKK-----------------------------------------------------------------------
CANGL 1 MSDGSADRK-----------------------------------------------------------------------
KLULA 1 MMRDPSRRT--RS------------------------------------------------------------------E
PICPA 1 MSETETLRD--G---------------------------------------------HFHTSGPTY--------------
CANBO 1 MSNMDSVALAQKSSSEKI-VSEL--------------------------------------------------------S
CANAL 1 MQNICRKQY--LVVESVYMLPNARRASFEFFLFAQKKNYSPISKPFLLGTRSSRIFTHGSKTTTTVFE------------
DEBHA 1 MWVDSDTQI--NG---------------------------------------------SSSSGSPIIV------------
CLALU 1 MASLVQKAM--PTRKRKLTGPRT---------RSAL-------------------KMSSSRETSPVIDLTQDGEKDDCDT


HANPO 23 GRDKA-DRLG------------KKFADAKRNNRSRFAPSR----------SAESPY----------------NVLTSPSK
SACCE 10 --KHTIDRID------------QHIL-----RRS--------QHDN--YSNG-----------------------SSPWM
CANGL 10 GKMRALDILS------------KHLR-----GKN--------SDKTTPTRYGHKSVE----------------L-ESILR
KLULA 13 GKTKALDALD------------KHMT-----QRS--------TLKKSLAANGPNVLA----------------L-SESLV
PICPA 20 --------------------------------------------------------------------------------
CANBO 24 GKDKA-DILT------------RRVSAAKKHKLS--SPNRETPVKKSYTANGQTSSQTNTSNLGKTHLNNAPTVATEPLP
CANAL 67 ------EKKSTYRYQQLIFLFLLGFLDFVKNCNSLFFLY--ANEELNYL------------------------------R
DEBHA 22 ------EKPH----------------------NHLGIVD--STSESSFAKSGTHPID----------------TSHGIQK
CLALU 51 GTHKK-DHRK------------RKGAGGKANEKA-K----GK--------KGSPRK----------------DLANESDK


HANPO 64 --NDP-----------------------------VKLSRLYSIMNS----------KRST-NN-----------------
SACCE 38 --KTN-----------------------------LPPPSPQA--------------------------------------
CANGL 48 --KDN-----------------------------IVSSPLES-----------------------RDHQES---------
KLULA 51 --LNH-----------------------------LPS-------------------------------------------
PICPA 20 --------------------------------------------------------------------------------
CANBO 89 TTSNPSSSTANNNNSSTTGASNSLSPMQTPRRTTAKLSRLYFHLDS----------SQPT-PPPSILHQQQTRKNVHQIP
CANAL 109 --SLN-----------------------------MPPRRKKLSKETKLASLSKKQSPIVT--------------------
DEBHA 56 --RDS-----------------------------FTPRTRSTHTNS----------EV----------------------
CLALU 89 --AGP-----------------------------ANETANESDKSS----------PAIETTEE----------------


HANPO 85 ---------------------------------KSKRQR--------------EEPALQRSAS-----------------
SACCE 49 ------------------------------------------------------------HMHIQSDLSPTPKRRKLASS
CANGL 65 ---------------------------------------------------------QRINNPHSNTASPTPKRRKLNTQ
KLULA 57 ------------------------------------------------------------NSENVYQRSPTPRRSRESDV
PICPA 20 ---------------------------------------------------------------NMERFSPLPIAS-----
CANBO 158 DVKSPKLISNTQLQTPHKGGEKIQDQISDNNAPILRRTRN---------SPNRLTQSQQTQNQ-----------------
CANAL 138 ----------------------------------TRRRRSARKLSHENKN--SPSRTQKIEDDIGVDTQMSEE-------
DEBHA 73 ----------------------------------------ARGLNENDKD--NETTSEMTSDSSIIDMNISEDKLESGDL
CLALU 112 ----------------------------------SNETR--------------EPVTQKTSRESVKENSKSPERQQSLSV


HANPO 101 ----DISQAQVSTDENASI------------PQDALV-----------------------------------TRSQP-AI
SACCE 69 S-DCENKQFDL-----SAI------------NKNLYP-------------------------------------------
CANGL 88 AISSDNKTKQV-----LSI------------GELSHPDMQST-------------------------------PARS-RI
KLULA 77 V-MLEDKSSGL--------------------------------------------------------------------I
PICPA 32 --------------------------------------------------------------------------------
CANBO 212 ----KLLSKQNSSEQKSSVTSNTLNNRRLVTDEFSGS-PKPPSTKIISMASSSPSKISKSSPTKTSTGFVLTKSNRK-RI
CANAL 175 --------------------------------------------------------------------------------
DEBHA 111 ----DVSDGNL-----AKI------------PKGELS-----------------------------------TREEKNYI
CLALU 144 ----DHTQNNG-----IDI------------PQYSHTTEHPHSTEKTDLN------------------------------


HANPO 129 ATPNIVDDPST-TD--------------------------------------PRAGEQSGPSPNKSMESK-SQSQSKALQ
SACCE 88 --EDTGSR---------------------------------------------------------------------L--
CANGL 119 MKKDIGDQ---------------------------------------------------------------------N--
KLULA 88 FEEDAGEK---------------------------------------------------------------------A--
PICPA 32 --------------------------------------------------------------------------------
CANBO 286 VISNLFDQRQVQSSPIESKTKVTIPQNEPKDRVSNVVTRLQKQDETKSCGCNAKKENTKSPSPSRKLTISTKKISTQSEA
CANAL 175 --------------------------------------------------------------------------------
DEBHA 135 NIPPLADE------------------------------------------------------------------------
CLALU 173 CSKPIDAVLAS-ISKAQ------------------------------------SPKHVHSPNKNNSFEAN-NDKHDGN--


HANPO 169 TAPNGNSQSTPTKGSQPSPSQFGSNNTHLA-------------------------PGQRHLRIS----------------
SACCE 95 --------------MQSLPELSA---------------------------------------------------------
CANGL 128 ----------------------M---------------------------------------------------------
KLULA 97 ----------------------L---------------------------------------------------------
PICPA 32 --------------------------------------------------------------------------------
CANBO 366 KSQKTPPPESP---------LSGKNESKLVEISKQKKIMISDSLQSNDNQDPFVQPRTEKIRIASSQSNIQKSPKTSTLS
CANAL 175 --------------------------------------------------------------------------------
DEBHA 143 --------------------------------------------------------------------------------
CLALU 213 LLANGETEK-----------------TNAK-------------------------PHT----------------------

HANPO 208 ----KQAF---EKNIAQ------------ISDNISTFQAVVRNPARTSHTDQ----------------------------
SACCE 104 --------------------------------------------------------------------------------
CANGL 129 --------------------------------------------------------------------------------
KLULA 98 --------------------------------------------------------------------------------
PICPA 32 --------------------------------------------------------------------------------
CANBO 437 SKKKLLTFVKDEKQLENITNNTLEISGSTTESKIHTAELEEPSTMDITHNDQIDNDIFVDAQEFTPNQDLVNVSKIIKEL
CANAL 175 --------------------------------------------------------------------------------
DEBHA 143 -------------------------------------------------------------------------------F
CLALU 229 ------------------------------------------------HSEKTH----------------------HSHH

HANPO 241 --------------QS----SA----EKTPK---------------------------------EPAVPTPQTPTRQA--
SACCE 104 --------------SNSDNVSP----------------------------------------------------------
CANGL 129 --------------SSSEIYSP----------------------------------------------------------
KLULA 98 --------------LS----SP----------------------------------------------------------
PICPA 32 ------------GLDQ----SP----------------------------------------------------------
CANBO 517 RNNKESVNDENIILDS----SPIRLEAPKRKLEAPKINTLFSASKSTESPESKIVNHKTALLSDLNSSPIRDTPFRETLK
CANAL 175 ------I---AGFLNS----SPIKQIDRRSN----------------------------------NSTPT----------
DEBHA 144 MNDQAYM---DMELNS----SPIKPTSKKVR----------------------------------Y--------------
CLALU 239 RSHKPYS---HEFLDS----SPIKRPHGSPK---------------------------------LLLSPTKPFPKRRP--

**RVxF/SILK**

HANPO 264 ---GSRDA-AGNQTGSDKKVLFSGNVDKS--P--IASSP----------IK----EPYEPRSILKVSPPKAD--------
SACCE 112 ---------------VTKSVAFSDRIESSPIYRIPGSSP-----------KPS-PSSKPGKSILRNRL--PSVRTVSDLS
CANGL 137 ------------S--VNKSVSFLDESNKNSESTSSNSSP-----------RHS-PTSKPKKSILRNSS--SPEHNKQR--
KLULA 102 ---------------IRKGVSFSDRVESSPTMQTMGSSP----------IRPSSMTKPPARSILKHPS--LH--------
PICPA 38 ---------------NRKGVKFSEDVQLSPPK--IMSSP----------RR----RYTATKPILKSSS--PA--------
CANBO 593 KNNNTKDILKNINTPVRKQVIFSSDVESP--S--ILSSP----------LR----SSFEPKSILKQRQ--HD--------
CANAL 198 -----KNTLSSSPIKTKKSVAFSDDLISD-----IPSTP----------DR----NHSSGRSILKSCN--SE--------
DEBHA 169 -----REELSSSPIKKKKSVAFSDDLVSE-----LPSSPAAVHCGSRLPVL----EHTPKKSILKINS--LN--------
CLALU 277 ---G---SPKKSPTSPRKRVAFSDNIASD-----DFPDP----------FSDNFSDGTPQKSILKRPV--QA--------


HANPO 314 -----LP------------------------RSVGGDIDEEFPAGSLMQREPND--PTSIELIRKCYRLLQKED----NP
SACCE 163 YNKLQYTQHK--LHNGNIF----TS--PYKETRVNPRALEYWVSGEIHGLVDNESVSEFKEIIEGGLGILRQESED-YVA
CANGL 187 MND-KYGKPI--AANVNLS----RSE-NHSKNQYSPDNLQFWGNGEIHSLSSNNSIAEFEKLFLGALKILMQSKNPEYGA
KLULA 147 -----YSKHD-RPSSRN-S----PS--KASHLSIDPSSTNFWVEGEIKSMINTNNVAEFKKILKGGLHVLNVTK-----T
PICPA 77 -----SSA------NSSPL----RK---PKGSGYLPTDLSFWSSGSIVATAPGT--ERAIAVLIGSCKVLRDPN----FK
CANBO 645 -----LSS--------SPSKETDSY---NKQLYINLSQNESWSSGQIIQLSPKS--VQTDSVLTQCIQALLTKG----FV
CANAL 244 -----FQNRLVDPSNTSLW----RKA--NENVSYGPKNPDFWLQGTIVQLPPNS--PDLYHLIEGCITVLQDAS----FD
DEBHA 225 -----QTSSPCNPNDTSLW----VKSSNNINIKNSPSNPEFWLSGTIIQLAPNS--PDLPQLIDGCINVLRDKK----FS
CLALU 326 -----LVH----------------C---FSAARSSPGDRAFWHAGTIVSLAPRS--PDLPELVAGCAQVLADPD----FD


HANPO 359 RRFEILATLHNTLKINSVEFNTRELNKAD---VSS------------------------------IA-KVAC--------
SACCE 234 RRFEVYATFNNIIPILTTKNVNEV-DQKF---NIL-------------------IVN----IESIIEICIPH--------
CANGL 259 KKFEVYATINNIMPSFSSTTSNQFIEKAA---HIV-------------------KDN----LKIIANISIEH--------
KLULA 209 RDFEIYATFNNVIPSMNGVILNDIVHQKI---DVL-------------------IEC----LDELLDTSIYA--------
PICPA 133 HKFEAYATMNGILRENKINFIKTPLSEYS---E------------------------------YLIQETLED--------
CANBO 703 KRFECYATIHYILKNNSVQFLSPILFEYS---KKL------------------------------VNIIIVD--------
CANAL 307 KRFEVYATLNGVYKTNSGSGAVKLFSSTLEDSVLASPRKNRSTPSPLKNNSPTKKQRESSYVALLAGQIIKDIEATEDLL
DEBHA 290 KKFEVYATLNHICRCNTSDSLLKLLTIPS---KSNSPQKINSK-SSMKTNST--SFMDNTYIHTLSAFIQRDVKQIELKL
CLALU 376 RKFEVYATLSHVCKSNDASTLTDLLVRPA---SRMDIPNDNDK-SMA-AHVA--YGGSDSLASRIVDFARRD--------


HANPO 397 --AEIARAYV--SPDMDFDAVESRTTILGVKLITTVMSMFEVS-----------QTAEIVEMLCTLVLRKRISKAMASAF
SACCE 279 --LQIAQDTLL-SSSEKKNPFVIRLYVQIVRFFSAIMSNFKIVKWLTKRPDLVNKLKVIYRWTTGALRNENSNKIIITAQ
CANGL 305 --LRDEQNSLL-QDKEKKNPFSIRLYIQIVRFFTNLFGSFIINKFLISSKALCERMKQVYELSLEALKNENSNKMMLICQ
KLULA 255 --LSHLQESLL--LQQKKDPFKSRCFIQVIRFLTLLFSNFKIIKFLDGNLSLQLKFIEVLKACEESLTHSNTNKVMVIYP
PICPA 172 --ICHYEAYLTNTTNSELDPFLLRTTIQSIKVSSFILSNPDLSFKLSN-----TLCSKIMVKCCELLTNQSLGKSLSAAL
CANBO 742 --NSITKTG---QLVDLSDAFQSRLTTVSIKVLSLLSSHPKEYNIE-D-----TEIHRALRYISFLLQNESLPKSVCSAV
CANAL 387 FDDNK-DKEN--RSPTKNDPFRIRIVNQATKLMSYFILDQELNKLISL-----HNIDWLYHHACVMLTHPKASKAIISAY
DEBHA 364 FCENEEKENN--YSPSKNDPFSIRIISQALKVINFIMLDQELNNFVSI-----EDAKWFYVHCCKTIVKPTISKTLIIPY
CLALU 441 --VLSGEARL--FRAHKNDPFQARILGQALKTVAFFFAVPAVNNALAS-----ESLSWFYGHACDMLAHQAISKSLVLPY


HANPO 462 VQFCKEQSCI--------L----PGGSSEMAINALMTMPFYPSGTIVHEKILALKKIGSNAPASMLKTCNKWFPFILASI
SACCE 356 VSFLRDEKFGTF-----FL----SNEEIKPIISTFTEIMEINSHNLIYEKLLLIRGFLSKYPKLMIETVTSWLPGEVLPR
CANGL 382 MTFLRDEKLSYI-----SF----TEDEGKHMIQIINQIRDIPSINAICEKLMLIKQFLIKFPGPMISTIPEWLPTQVISK
KLULA 331 LTLLKEEKFGQF-----YL----PDSQIRQLVNSVFTMKYIDSTNVQCERLMVLKQFLQKYSKVMLETLRTWFPSEVAAR
PICPA 245 LQLIKEESVDHKV---ISL----NPYFAETLLSSVLNMKYFHSASLLMEKLFIIKCFLIKFKRTMIKNFNSWFPFVFACL
CANBO 811 LQVIKDHKSIDT-----PL----PNPIIESILQSLLVVQNFSSFTITVEIVSILADFINKYKGIMTKHSTRWMSFLLSTF
CANAL 459 LVIMKECKLSAKKKKALFE----TGDLAEKMLFALINMKRFPSSSLVSEQFMCFKNFVNLFPGIMAKNISHWFGMLLLNI
DEBHA 437 LSILKDCKFNNKKKKLIFNNQNENANISELILQSLLNMRKFVSSSLVVEEFVTLKNLVSNFPVMMANNFKHWFEFFLMNL
CLALU 512 LCLLKECHYSARKRP-QIF----SEDLPDRMLSALLAVRSFPSSSLVNEKFSCLKTLVLNFSEQMAARFDDWFAPLLADL

HANPO 530 MSIDL-GSHQRILQAVVALLNEWTSNCN--PKIREDVLTLMSEDAETFLSLHAVE--------------ASQNLLEPGLS
SACCE 427 IIIGDEIYSMKILITSIVVLLELLKKCLDFVDEHERIYQCIM--L--SPVCETIPEKFLSKLPLN---SYDSANL-DKVT
CANGL 453 IVFSEDVYSTKTGFTAISVLLELLKRCIDHSIGHEYVFQCLH--I--DSTALYFGDSKDKKLQWSNNTLEQTYDI-NETH
KLULA 402 YLMEDRVTSAKTRSCCNLIILDLLRKCISNDKVRTAIIEIESLSL--KSVVENCP--FGTEVD-----VEYTEFI-KDQT
PICPA 318 TDTSH-PTFFKIIQSATNTLRECSAQFQYNRSMNELTNIFLTSPM--TSSMQSIQ--------------SDT-NR-SKAK
CANBO 882 LEYGSIPSAQRIVRAISFAIQ-QTVNCV----------AITGKSKISFHNIMKLA--------------GSDNDN-SEVS
CANAL 535 CEVGS-PFYLKCFSVGVHCLLEVAKAFLDNRKTRAYVKLFLSSPF--SFNIKSMS--SSESIVID----SDSNDS-QSQK
DEBHA 517 CDLSS-PLYSKVIGMGISVLLEVARNYLDNKNVLFSVRRLLASPL--PSTVKSIT--SDSTISVSPE--APTTKS-DENL
CLALU 587 CNSSD-SLHVKIAGSGITTLLEAARTYLDTVKVGHSVRAFLGRPF--VPTGSFVS--------------ESANVV-SEMN


HANPO 593 VCDLLVKTLNITL--RRGAYVEAMKIWALVTYSISAFTWNKGIEDWPHLEAWVSVFTECFNAD---GLPPKIAALEAWKG
SACCE 499 IGHLLTQQIKNYIV-VKNDNKIAMDLWLSMTGLLYDSGKRVYDLTSESNKVWFDLNNLCFINN---HPKTRLMSIKVWRI
CANGL 528 LAAILRDHIDYLVN-SKREYKLAMDMWLSFMGLLFNSKGRLAFLASKEGEKWVDINLNCLSVN---DPKCRILSLKVWRI
KLULA 472 LGTALCNKLISLMN-DKEEYKLSMDFWLGMTGLLFNSTKNLSLLLEPVGSRWLAVNDVCFNSK---KSNLRGIAIKNRRI
PICPA 379 GIDVLVQNLIKLSSMSFMECSTAINIWTQFTLLFNVFTATNSFDLWSFNQLWLSPLA-YFEENGPQDPKSLLEVFKCWKI
CANBO 936 LIDAVLEVLTSYL--K-LDFYTGITIWLNLTYLQNH-----QF-PLDHVQRWISVILPYFDND---DIMIKKSCLFAFKS
CANAL 605 VVDIVLSKLSELI--HDGQFKEAMDIWVALTVLVGY--SGESFERWEHLSKWLQITKLCFNSQ---VPQARVLALSCWKA
DEBHA 589 VIHFIISTLETLI--GNGQYKSAMDIWVGVTLLSSD--SGSGYENWPFLSDWLRVHKSCFNVN---NNSAKMIALNSWKA
CLALU 649 SIDYVCSSLKTMI--DSGHYKFAMDIWVGLSLLISH--AGLAWEQWEYLPQWLSVHRHCFNVA---SVQAKTTALSSWKV

HANPO 668 IIFAYQSSMV----YRASLSPKT-------KSEEELIDVKFHVL-AHPFFGLAL--------KLNDTLFVEYQKLFVRIY
SACCE 575 ITYCICTKISQK-N-------------------QEGNKSLLSLL-RTPFQMTLP-------YVNDPSAREGIIYHLLGVV
CANGL 604 LIYCVTNNIDFL-G-------------------EKEAKKISQVL-LNPVNVTVN-------ELEDNSTFMGVKYLLNDFF
KLULA 548 LNYMIITTITHDID-------------------NHVLDALIQIV-MRPFEFP-----------DNEGLSEYIIFAFNSIM
PICPA 458 IT-QYCFFSEKLNSFPSNSSPE---------KLNK-----MTLLIGQPL-AIFHRKKIEIGSKLSKEIASYQDWVFFTIT
CANBO 1004 IIYELHSDIF----FN--------------SKDIELRDEIFKIL-IIPFKKTCN--------------IKELEQIYVQLL
CANAL 678 IIFNLCRNDLDEIRKTLDPVMGHSNIKDKQQQITTVMKPKVKLL-TYLFGSFNA-------AEMEDEVIDTLHNLFVAIL
DEBHA 662 VIFNVCHNDLDSIRCLIDSSPNSKSSKSKPQAINNILKPKIKLL-IHPLLNISA-------VENQKEIIDSSHNLFLSII
CLALU 722 VIYKVCCVDLQNPPFASLSAPSL---ASSRSRLIDEIKPKSKLL-IHLFVNIIS-------AEYRPEVTDALHHSFLSIL


HANPO 728 H------------------------SLYKFLTRNPQDEGA-SMNGAIKW---ICTILGQ---FF-LAESRSAEQLEFAIN
SACCE 627 Y------------------------TAF-----TSNKNLS-TDMFELFWDHLITPIYED---YVFKYDS--IHLQNVLFT
CANGL 656 F------------------------STC-----GITNKCK-PTTNIVLWGNFIFPMLATLLNYSANEDVK-FELDTYIGK
KLULA 597 Y------------------------LTC-----CDYKDMS-SKRFALLFEVILKPLFQR---INQPTLL--PILGPQAHQ
PICPA 522 KASKSTTDPTCKQQLFQNSFVAVLN-------------------------------------------------------
CANBO 1051 T------------------------NFYEFL-------------------------------------------------
CANAL 750 Y------------------------STI-----NPLVIKSRTKYLHILWDKVFQFVFIN---FYFKKDASNARMSQLGFA
DEBHA 734 Y------------------------TLL-----NPSVLNS-SRYVHVYWDKIIQSVFIN---FYFKKELSNTYMNELGLK
CLALU 791 ------------------------YNLL-----GPLSAPS-LKMLSAYWERIFVPVFAH---FYFNKESANPQMHRLGAM


HANPO 776 FFEQMLT----SNPSKPDL----AQTKLQKDCFAD-D---I-AIDSL-------------------------------SV
SACCE 672 VLHLLIG----GKNADVALERKYKKHIHPMSVIASEG---V-KLK---------------------------------DI
CANGL 705 LLTKAIQ------NQKN---VDKTKSLHPIKVIASEG---V-SVD---------------------------------DI
KLULA 642 ILPRIIRLFSDEENSSP----RRSIGFQPLKALSTIG---I-DLP---------------------------------DF
PICPA 547 ------------------------QVFMGTRSKLDEAGSEL-AVN---------------------------------YI
CANBO 1058 -------------------------NIKNLKCCDNKE---LEKINEDNDNMDSDRSDHSDQNVDTEKEDKDKLYLNLPIF
CANAL 798 VLSKLIK------SATPVN----ERNFNEVRCLSNEP---V-SIN---------------------------------EV
DEBHA 781 ILTRLIK------PSVSTN----EKPFNEMRCLSNES---I-SLN---------------------------------DI
CLALU 838 VLSKLLK------PTNPVN----EKNFAWTRCLSNEA---I-SLS---------------------------------EL


HANPO 812 PPINT-LWVATSPDQFVELLKIACKS-KI---------------------------------P---VAKKLK-LITLF-V
SACCE 711 SSLPP-QIIKREYDKIMKVVFQTV---EV---------------------------------A---ISNVNL-AHDL-IL
CANGL 739 SALQL-SQLPYLLETLGSVILLYV---LN---------------------------------S---KSQNHSQTRML-IL
KLULA 681 EPMSN-TILENNWNSIVELLKGMIAWPGF---------------------------------N---SQNSLM-MLQHCIR
PICPA 569 SSFTDCSW--------APLLLSLSVSAML---------------------------------E---IL------------
CANBO 1110 EPLKY-NIKEKCDDIILPFIENIIKG-EVSKGSHFNIFEPSELIEYIHPQPHYPKNGLINLLPLTKLENVFE-IFNYF-V
CANAL 831 NSLPP-RWIYSKFDKIMQNMILIFLSENL---------------------------------H---VEDKIG-FFIAF-L
DEBHA 814 SPVSP-KWIFSKFDRILQNINIVFKLEGL---------------------------------T---IDSKLE-FFNNF-L
CLALU 871 NSFSP-RWVYSQFENIMPLLATICKSEHL---------------------------------E---TEQKLT-AINGF-L


HANPO 852 RGLRT----GLSPSSV------------AFTRLT-----------------------EPVIAIF-LDTFSE--VEQD---
SACCE 749 TSLKHLPEDRKDQTHLESFSSL----------------------ILKVTQNNKDTPIFRDFFGAVTSSFVYTFLDLFLRK
CANGL 778 EYLEQIPNNQITLSTLCRCISMFYEYYNSIDF-IANSPRSLISDMSRVCIKFKDI-IFDDNYNV-FRNFILSFLMLFQEE
KLULA 723 RSPSVIPSNQACAQSLEYL-KL----------------------ILEKGEIDRNEEVFKTIIRSLAEKY-----ELHLFS
PICPA 593 -------------------------------------------------QRN-ITLEEGNV-KL-LESYLTSALTEM---
CANBO 1186 RTT-------TNEETI-----------------------------------------KDMFTVL-IETFNNPGIEKD---
CANAL 872 ASIKPIVKNEASVSPT------------TFDI-IDNIPIVL-SHLFKTNTLS-HDLAIKLIVNL-HDTFTSSLLVRRANK
DEBHA 855 NSLKSITKKEIKPSDA------------TQDI-IDNLPNVL-SVLFKHNKLS-YSSIQKLMLNL-NDTFDASNLIEKNGT
CLALU 912 SSIKYITKKEIQPSNT------------TLDL-VDNLPYIL-QPLFESSEPS-YDEIFKLIVTL-NDTFGAPHLVAEA--

HANPO 887 -----CAELGNLEEIERVLSDF-----------FQAIDILNSLYLW-G-E-TKSVFTVV--------------SQWLRK-
SACCE 807 N-------------DSSLVNFNIQISKVGISQGNMTLDLLKDVIRKAR-NETSEFL-II--------------EKFL-EL
CANGL 855 E--------------------------------HHVDNLLQELIQNALQANISELK-LI--------------EGIL-SL
KLULA 775 N-------------SSILLTKIFPMDQVT---SAVQFEMFKNIFQLVK-PFVKPLL-LF--------------GYFD-RF
PICPA 618 ----DPSTRIDFNVFYSCWFKL------TGGP-ERMIRSFPLVEVIHY-H-RTLKL-PL--------------KELL-RS
CANBO 1214 ---------------------------------PRFRKCFQLLCDFAQ-E-KKLLLDFIQDRFVEISGEFELNELFENDK
CANAL 936 QDEIDTSNNIYLPVLANCSKSL------SK---EEYLEIFQLITQSLS-H-KKILV-FI--------------ADYL-QS
DEBHA 919 R---SNATNIYILIINNCLSDM------EE---LQAFDFLESIHLAIR-D-SKNLI-FL--------------LELV-KL
CLALU 974 ----KDGKGTYQVILSFCASHL------TS---HQMNAIMSMLHGTVG-E-RKSLL-FL--------------SNLT-QL

HANPO 933 ---LD---DKNAQHGSN-LVAGYLDKIF--SLDVNRYVQACNELLTHCQG-FEKL---------------------VTDS
SACCE 857 DDKKT-EV----Y-AQNWVGST-LL--PP-NISFREFQSLANIVNKVP-----------NENS-----------------
CANGL 887 KKTDC-DL----F-AINWVGKK-LL--SP-SVQEGDFILYSRIVAKIS-----------EKQT-----------------
KLULA 822 SNDLI-SN----Y-IANIVGSM-LI--PT-NMSQQEYKSLLQIVNKMP-----------VPEV-----------------
PICPA 669 WY---SIERMQVI-FL-----QRLLDYSTRENDLEIISDVVVPILSD----GEGI-------------------------
CANBO 1259 DNKND---NSIVCNMSN-IV----------------YGPLSSEIRTNFKNDFFKLVKKRRITLYFELIKTGSKTTLL---
CANAL 989 --GLK-MEDVKAV-ISDILAKR-T-VELS-SEELGLYGEICQYFDTG----FETF-------------------------
DEBHA 969 QEYNT-HAGLKNF-IVSALNSR-KINAAS-NLELELAGNLFQIISKD----YESI-------------------------
CLALU 1023 SKSLDSSGEILTF-VEDCLNNK-KYSRFS-HQDMILLSSIFESLDKN----FATV-------------------------


HANPO 982 LVAHVCSAYFNASPVVWTSIFEK---------LGE---SIYIDILSRL-VLNEVG-HDS-AIDAQKHDKFWQATKLKTVS
SACCE 899 -------------------------------------------IENFLDLCLKLS-FPVNLFTLL-HVSMWSNNNFIYF-
CANGL 929 -------------------------------------------IVNFLDLADKLG-FEVNPTEIL-RISFWKPSGLVIF-
KLULA 864 -------------------------------------------IDNLFTWLRKTD-NWGSIAGEL-NLSTWNDHLFANF-
PICPA 711 ----------------------------------------DSGLIEGT-I------------------------------
CANBO 1316 ------------SPVLLHEPFTKDQYKLDRDAIRLGLKTVPISMFIQF-LVLKKD-NIS-LIDEL-EPEDWHIGNIKFL-
CANAL 1033 ----------------------------------------VKKLIQTI-VVVSDSEKMLNCFENL-NITSWNYSIVIFL-
DEBHA 1016 ----------------------------------------AKKLIQSI-VLLSAT-EFERVSGIL-DIQLWTIPIFKYF-
CLALU 1071 ----------------------------------------AKKLIQQI-VLLKAE-EFEKIVNEL-RINHWNISIFKFF-

HANPO 1047 DEQLYVLYDVLLSCPGTELLRYQVSNCLLN---------------------------GKPTLYLPVV-------------
SACCE 933 -------------------------IQSYV---------------------------SKNENKLNVDLITLLKTSLPGNP
CANGL 963 -------------------------LEEYI---------------------------AKHHVDFADIVSSIIDADRLNNV
KLULA 898 -------------------------IQKWI---------------------------QEQKNSWSQETVRMLTESLWNRP
PICPA 720 -----------------------------NIVTRFNEELFFGTDKRIILKLFKEFARENPLPVKNERLGKLLAAEF-VSS
CANBO 1379 --GES----------------------LVT------------------------LKSKVSIMYKDRA-------------
CANAL 1070 -------------------------LLLLK---------------------------NAPNKHIFQFTINLIEKMM---K
DEBHA 1052 -------------------------VELVH---------------------------NAPHPHLKQIPLNLIISRF-END
CLALU 1107 -------------------------IILMH---------------------------DAPFDYLKRVCVKLIRQRL-QLE


HANPO 1087 -------NRVLKRIIDQGQCP-----------------------------------------------------------
SACCE 961 ELFSGLLPFLRRN-------KFMDILEYCIHSNPNLLNSIPDLNSDLLLKL-LPRSRASYFAANIKLFKC---SEQLTLV
CANGL 991 ELFSYVLKHLPEN-------QKSAFIKQHYAKCPLIWNQGVLIDDYDLIDITLDCEICTFIAHHFSFLST---SSKAKLL
KLULA 926 AAFKELTALLINA-------EQTQIIKDTLEKNPDIIDDISPLGDLSLLDI-LPATL---VKSSFSKIQQYDDVIKTKLF
PICPA 770 NTLPQCLFQ-----------------------------------------------------------------------
CANBO 1398 ----DTMRKKIDR-------L-----------------------------------------------------------
CANAL 1095 SNFIATLELLTVQ-------D-----------------------------------------------------------
DEBHA 1079 EEFFEILKFLIDK-------R-----------------------------------------------------------
CLALU 1134 NEFEDLFVLLLED-------R-----------------------------------------------------------


HANPO 1101 ---------------------------QEELQLFLRK---RSS-LIHHNA------------------------------
SACCE 1030 RWLLKGQQLEQLNQN-FSEIENVLQNASDSELEK--SEIIRELLHLAMANPIEPL----FSGLLNFCIKNNMADHLDEFC
CANGL 1061 IIFIEKDMYDLLVQFRYNLIQEFLPTLT--IYKK--DDII------------TTV----QQHLINYCFAKNSFVLLDCIE
KLULA 995 LCISTLDEISIIKSNTELLYQLLLPTDDDVSLNNDRNIIMDMLLKSSIGHQDWDL----LSMLFEVCLLRRDSDKIVDFL
PICPA 779 --------------------------------------------------------------------------------
CANBO 1408 ---------------------------FDELLLKLKT---AAL-KSFNNGNGENINN-----------------------
CANAL 1109 ---------------------------FEAEIFPLMDQIYTNS-LQYSGETLFKICSLLKDYLEYKLKNSENHELVDRLL
DEBHA 1093 ---------------------------FDLELYNLRKNIMKKF-KALEGFRQFEFRSVWNSYLSN-IVESGNFKLLDDFL
CLALU 1148 ---------------------------FDFEIHTLKDTICERL-ASFNNEEKQSIINMWHNYLSS---FSGDMKKLDDLL


HANPO 1120 --------------------------------------------------------------------------------
SACCE 1103 -GNMTSEVLF-KISPELLLKLLTYKEK-PNGKLLAA-VIEK--------------------IENGDDDYILELLEKIIIQ
CANGL 1121 PSTLTSRTL------EDVIKLLEKSYE-DNITFVPAKYIIFIDNLPMDTK-INTTEILRVYLVTGDVTQCLFFLTSLIND
KLULA 1071 ---TAGKIKFENIKPNTIARMINESGK-LNSDLIDF-LRDS--------------------FKNLNPSYVIDLMEQLLKQ
PICPA 779 --------------------------------------------------------------------------------
CANBO 1434 --------------------------------------NNDYKKFIYFSSFGHSK-------------------------
CANAL 1161 ----------------------LGCYSVLGID-VSTLIQDDYSKYPNFKI-ELDK-------------------------
DEBHA 1144 ----------------------VSSYE-VGLD-VRSHIQNKWDRLPLLKK-AWLT-------------------------
CLALU 1197 ----------------------TTSHN-SGLD-IKSIVKNRWETFPNLKK-AWLK-------------------------

HANPO 1120 --------------------------------------------------------------------------------
SACCE 1159 KEIQILEKLKEPLLVFFLNPVSSN--MQKHKKSTNMLRELV-----------------LLYLTKPLSRSAAKKFFSMLIS
CANGL 1193 KKMVPLTRCRNEVFDFFIALDSSNDLSIKAKAVTTFESLIN-----------------VIF---LLRKKVDIGFLSQYIA
KLULA 1126 TKYQVFDIITEELLSFIFD--IKQQLSADD--KERLKGIFPAIVDYFVGSNPKILTDIMKYVVNIMKSAKEKQYGTSLIV
PICPA 779 --------------------------------------------------------------------------------
CANBO 1451 ----------------------YNSG------------------------------------------------------
CANAL 1192 ----------------------RNSC------------------------------------------------------
DEBHA 1174 ----------------------DNSK------------------------------------------------------
CLALU 1227 ----------------------TNDA------------------------------------------------------

HANPO 1120 --------------------------------------------------------------------------------
SACCE 1220 ILPPNPNYQTIDMVNLLIDLIKSHNR-KFKDKRTYNATLKTIGKWIQESGVVHQGDSSKEIE----AIPDTKSMYIPCEG
CANGL 1253 RLPERPSRYLVDLSEKLI-LNKSFNRSKFRNSSSFNDLHTHVRTWCNQ--------------------------------
KLULA 1202 LFLNHPDFKIQGNK------------------------------------------------------------------
PICPA 779 --------------------------------------------------------------------------------
CANBO 1455 --------------------------------------AN-------------------------GNR------------
CANAL 1196 --------------------------------------IVNGKIVVNDLQKSQESDDK-------SSQ------------
DEBHA 1178 --------------------------------------LYFDSELLNQTGPASPGDSK---NSEVSGK------------
CLALU 1231 --------------------------------------LF-------------------------SSH------------


HANPO 1120 --------------------------------------------------------------------------------
SACCE 1295 SENKLSNLQRKVDSQDIQVPATQGM--K-EPPSSIQISSQISAKDSDSISLK-NTAIMNSSQQESHANRSRSIDDETLEE
CANGL 1300 ------------------LDNTSGSPRETTPISAAHINDHIN-KNALSLEKDKDNLTNN-TEQQ-----------KNIPN
KLULA 1216 --------------------------------------------------------------------------------
PICPA 779 --------------------------------------------------------------------------------
CANBO 1460 -------------------------------------------------------------------------------D
CANAL 1219 -------------------------------------------------------------------------------E
DEBHA 1205 -------------------------------------------------------------------------------E
CLALU 1236 -------------------------------------------------------------------------------E


HANPO 1120 -AGCN--M------FLGK-TI----------------------------------------KLVSRLQSMIEGVPETQS-
SACCE 1371 VDNES--------IREIDQQMKSTQLDK--------NVANHS-------------------NICS-TKSD-----EVDV-
CANGL 1349 SKSQEISKLSSNIA-------------------------DAN-------------------DLP----------------
KLULA 1216 --------------------------------------------------------------------------------
PICPA 779 --------------------------------------------------------------------------------
CANBO 1461 SSGNL--I------YLGS-DK----------------------------------------SICS-TESF-----ETDS-
CANAL 1220 QETSV--P------TDSDNSL-DLQVDS--------NIQKEQDVSESNEYDQSNIPELSAD-------------------
DEBHA 1206 DNTQI--I------AAPDCSK-DLQMDDMETIESIENIAIPKSFSNSNKDSKTNRYEKRNMSSGSDISQL-----EIDNS
CLALU 1237 SNTNV--I------SPEK-QI----------------------------------------EQLT-TRQV-----ELES-

HANPO 1149 -----SS-QS-SNA---KSET----EKPHKNGTKETPIPSVQQQLEPE----------PN-------------RPQ----
SACCE 1409 -----TE-LHESID----------------TQ-----SSEVNAYQP-----------IEVLTSELKAVTNRSIKTN----
CANGL 1369 ------------SN----------------TR-----ES-----------------------------------------
KLULA 1216 --------------------------------------------------------------TLL--KKLNVFKA-----
PICPA 779 --------------------------------------------------------------------------------
CANBO 1485 -----SK-SSSSLA---STGSKKKSKKNEKSSAS-----SKNKSLKNE--------NKNG-------------KVK----
CANAL 1264 ----------------------------SSTS-----ISDINTVDT-----------VNK-------------NIA----
DEBHA 1272 TKSKANRKRSKSVAKPKPKKKTNIKVKKENTP-----APEVNPNFD-----------IHS-------------F-T----
CLALU 1261 -----IIASDMLHH---ATTKTP-----------KLVLPSPTVEEEEPVNICDSADTSDQ------------THNATSVA


HANPO 1188 ----------------------------------------------------------KDAVQGAQTKQ-----------
SACCE 1447 ----------------------------------------------------------PDHNVVNSDNP-----------
CANGL 1375 -----------------------------------------------------------NCDGSQSTEP-----------
KLULA 1227 ----------------------------------------------------------------NAKRN-----------
PICPA 779 --------------------------------------------------------------------------------
CANBO 1526 ----------------------------------------------------------IECDSSADTKK-----------
CANAL 1283 ----------------------------------------------------------TEISSKSNLPEVEQVCKVNDII
DEBHA 1318 ----------------------------------------------------------AMLNAK----------------
CLALU 1310 AESEMPFTEQKKTSSEEAASSVDQEKRFVGQEKSSSDDVQLTYESNSLEHATESNNTKASTDTTASTSK-----------


HANPO 1199 ------SG-----------------------------------PEKSEQQESQTQEPER---------------------
SACCE 1458 ------LKRPSKETPTSENKRSKGHETM---------------VDVLVSEEQAVSPSSD-V-I--CTNIKSIANEE----
CANGL 1385 ------AG-----------------GKM---------------IKIDKGQISADMKNNK-I-A--PRQISSIASEP----
KLULA 1232 ------STRQYKFIPPKENKKVSDRETL---------------QHNEQQDNTNIFPLST-L-S--VIETKVTSSEE----
PICPA 779 ---------------------------------------------LLENGEI--DLNQY-YNA--IDNLTPYNPRV----
CANBO 1537 ------IE-----------------------------------NSSKESDESSINLNSD-A-N--ISSVTAHSQEG----
CANAL 1305 VTDTECAKDESKVQPTEEFES------VIQHNEGGLGLTVKLTPVKNRSAIETVSPKQ-EF-IVKIVKKKPEEQVQESSS
DEBHA 1324 LSPATPIKKARRNAKSNLNKS---------------------SPDNGSNSESCISPSGNEE-TIDLDHIKSEVQIP----
CLALU 1379 ------LK-----------------------------------RPI----------------------------------

HANPO 1217 ---------------------------------------------------------------------QDSSAT-----
SACCE 1509 SSLALRNSIKVETNCNENSLNVT--LDLDQ-QTITKEDG-----------------------------------------
CANGL 1419 DTLV------------------N--SNIDN-KSMIEKGK-----------------------------------------
KLULA 1283 -------------------------LEVPL-QTLSE--------------------------------------------
PICPA 805 SGYVY----------------QLAMKNLFLVKQLHLSYT-QFSQVDITETYVVPDL----------SKYATVSLD-----
CANBO 1568 STLVID---------------SI--SPLPSLSSLSSSKSDAFDSLDVKDTHIIDDS----------SLRSQTTSS-----
CANAL 1377 SAKDDD---------------EI--SDIET-SSVSQGNGNG----------IIEQP----------EVSSSRSSNGTLVR
DEBHA 1378 SSFNDD---------------DI--SQLEA-SSMSQNSINEFD--------LVEDEPSSPPETLDRT-------------
CLALU 1384 ----E----------------SASTSRRRSKRIQTRRAG-EITELSSDASIVIEIS-------------SDAKSD-----


HANPO 1223 -------------GTSTELTTFVLRSDDEEEEEQPID-KSGTTSEQPVATSSPQRAADQPASKPVIPQSSQVSSPIVGIS
SACCE 1545 ---------------------------------------------------------------------KGQVEH-VQRQ
CANGL 1437 ---------------------------------------------------------------------N----------
KLULA 1293 --------------------------------------------------------------------------------
PICPA 853 -------------SFPQSSLPDTVKIPQIT---S--KDLVL---------------------------------------
CANBO 1616 -------------GSTTKIINSSLDEGNVEIKDKKLN-NSGSN-------------------------------------
CANAL 1419 KREHPEESLAPSK--RSKVC-----------------------------------------------ESTNSAEDGLKVT
DEBHA 1419 -----RKSISPSSNDYSKVIQNG----------------------------------------FSSRESSDSSDD-IENT
CLALU 1425 -------------SSSGAAVSNSLEKPRTRSNSP--KDTSF---------------------------------------


HANPO 1289 P-TQVPMLPKATPQKASETSERAKNQPS------------------AQPHNVSPVPAT-SGLPSA--------------T
SACCE 1555 ENQESMNKINSKSFTQDNIAQYKSVKKA-RPNNEGENNDYACNVEQASPVRN-EVPGDGIQIPSG--------------T
CANGL 1438 --------------------GDK------TVNNNATTNDFEYRTSEDPIVSV-NIPI-----------------------
KLULA 1293 ------------------------------------TSDE--LIRTGCQKVV-EPIQN-SND-NE--------------P
PICPA 876 --------------------------------------------------------TD----------------------
CANBO 1645 --------------SEPEDSSTHIFPDA------------------QNIVDN-VAPIDYDSVDSSSSSEVKEDTNSLKRK
CANAL 1450 KTQLNAN--EKSLPLDDEIALENKQGELKMSNVDVDFNG-QQGENIQVDSCN-FSSINN-NS-SD--------------P
DEBHA 1453 DDQESTNYKRKTCYDEDEISGRKKQKQNEFPSDEKNDNG-TSAPESGISMTD-ESTGNS-NLDNH--------------I
CLALU 1451 --------------------------------------------------------SNGQSADSG--------------T


HANPO 1335 ------------PLSRAA---VPPALEVS---A-TAPRE-L---------------------------------------
SACCE 1619 ILLNSSKQTEKSKVDDLR---SDED-------------------------------------------------------
CANGL 1468 --------------------------------------------------------------------------------
KLULA 1318 VKMAASVQSEKLEAETTQ---NAVS-------------------------------------------------------
PICPA 878 --------------------------------------------------------------------------------
CANBO 1692 ------------AKFDIE---NDINKKLKLETTTSTPDIIKNCQSSATESSIAGASESEIKEINHEESTKVQKSTEDKEP
CANAL 1510 KTFAESIVST-DGEEKSENGEIPPAKELD---------------------------------------------------
DEBHA 1516 VHVSSSDQFRKDVNDSIENDE-IQCSATS---------------------------------------------------
CLALU 1461 KQVSDSDESSRLKEKDCS---NDVE-------------------------------------------------------


HANPO 1356 --------PSNHG---------EISEKQP--SVSRTQ---TSPPAVEQSTPSQQTPFGQSSIPPF---------------
SACCE 1641 ----------EHG---------TVAQEKH--QVGAINSRNKNNDRMD-STPI-QGTEEESREVVM---------------
CANGL 1468 -----------------------------------FNSRVSGLDKLN-----------------NQKISDEYVISSHDLE
KLULA 1340 ----------QVS---------SG----------------------E-MEKQ-TPSIKDSSTEV----------------
PICPA 878 ------------GEDEEEINNSTVSPKR-----------------------KRIGKHQQSKKIKL---------------
CANBO 1757 EVNAQQLIIKRHD---------QVCDRTPIELPQQ---------------P-----QQPANFPPG---------------
CANAL 1538 ------------------------------------------------------IECTTSPDLKN---------------
DEBHA 1544 -----------------------------------DQ---TAPPTF-DTQDF-RATSTALAEPSN---------------
CLALU 1483 ----------GHI---------EIASSVD--YT-----------------DRKNGTFESSQEPSF---------------

HANPO 1399 -REPAERSFF--------GQDT---------------SQ--FLP-PFPPMPYYGYP-------YYP------MMP-----
SACCE 1683 -TEEGINVRL--------ED-SG-----------------TCELNKNLKGPLKGDK---------DANINDDFVPV----
CANGL 1496 LARNGL---------------QN-----------------QAQL------------------------------------
KLULA 1361 -SECLPGVAN--------ND------------------------------------------------------------
PICPA 908 -DDNSRV-------------------------------------------------------------------------
CANBO 1793 -YLPGFQPQNMFPVNYPYPYTP---------------EQ--FQF-PFP-NPYTNFP-------YPP----YQFAPHPANI
CANAL 1549 -SDNSAILET--------NN-DINKKDTEEEKEKMKEEQY-SMV-QEAP-------------------------------
DEBHA 1569 -TETSSSIQL--------SD-S-----------------YNSMI-PNSPPVHVGDR-------SED------FTP-----
CLALU 1510 -SEQSMIINT--------SN-----------------------------------DVLNTSAESIK------RLS-----


HANPO 1434 --------PA----------------------------------------------------------------------
SACCE 1723 --------EENVRDEGFLKSMEHAVSKETGLEEQPEVADISVLPEIRIPIFNSLKMQGSKSQIKEKLKKRLQRNELMPPD
CANGL 1508 ----------HDMHEGYKKKSANLLTQHN-MRPPFEDVAIPTHKELKIPIFNSLILKSTNQQPMLPNSQRKVQSEV----
KLULA 1372 -----------------------EISKN--LDTVTSVNSPSGL-PLKIPIFNPFFTDVIK--LEED----------LASK
PICPA 914 --------------------------------------------------------------------------------
CANBO 1842 GLGYSVQPNSDKQAP-----------------------------------------------------------------
CANAL 1586 ---------------SYF--------------------------------------------------------------
DEBHA 1603 --------ESDSKSNNFL--------------------------------------------------------------
CLALU 1535 --------VV----------------------------------------------------------------------

HANPO 1436 -------------------TGIA-EKPP---------HM--HHMPMYYPMPYPWPAPLPGPEKAL-----D-AKDP---E
SACCE 1795 SPPRM-----------TENTNINAQNGLDTVPKTIGGKEKHHEIQLGQAHTEADGEPLLGGDGNEDATSREATPSLKVHF
CANGL 1573 ---AIRKRPLGNPSTQGQYTDIENERVRD----------------------------------YDDSQSLEDSADIRLHF
KLULA 1414 G-----------------DTYISGKRSLE----------------------------------LMNSAGDVASANCEGQH
PICPA 914 --------------------------------------------------------------------------------
CANBO 1857 -------------------QQLQ-ANQI---------RN--HFA------------N-MTLDRLI-----H-DKDL---S
CANAL 1589 -------------LPLKEFEEIKSKKE-----------------------IDDDFENSVGYRSN------G----I---S
DEBHA 1613 -------------STIGEDDEIDSQNL-------------------KEKEDSDNGPQDEGSTTN------E-NSEL---Q
CLALU 1537 -------------------EEIP--------------SM--------------------EA-----------ESGL---K

**CTD**

HANPO 1476 NWVKLEELVTRLARENPDGVDDLEETKKRKLEDDLFTLLTCLKKR-------R
SACCE 1864 FSKKSRRLVARLRGFTPGDLNGISVEERRNLRIELLDFMMRLEYYSNRDNDMN
CANGL 1616 PNKRARRIVSKLRGFSEYDMGQLSAEEKRNLRIELLDFLMKIEYHTIT----D
KLULA 1443 HTKKIMRIVSSLNDVDIDDVKSLSNNDKKLLRKVMYNFMLNLED---------
PICPA 914 -----QEIMRNL-Q--RTIDEGLEKSQREELIDQLLSTIVMLRNL-------G
CANBO 1884 IWNTLDKV-CTAAQKNDADI-EMSKTDRTALEEKLIDLVVKLRKT-------N
CANAL 1620 KSYSPMDLVDLLSTKSDAELACITSDEKYEMETKLLNLMVRLRNLK-------
DEBHA 1651 PCEAANNLTSYIENMSDVEISRIPQSEKYQLETKLMTFILRMRNIDSVE---S
CLALU 1550 R-CTLYDVKDWM-NNADSELAQMSPADKYELETEMMRFILRMRQA-------S

**Supplementary file 1.** Multiple alignment of Rif1 homologues from budding yeasts. Sequences were aligned using T-Coffee (Di Tommaso et al., 2011) and shaded with BoxShade. Species abbreviations: HANPO – *Hansenula polymorpha* DL-1, SACCE – *Saccharomyces cerevisiae*, CANGL – *Candida glabrata*, KLULA – *Kluyveromyces lactis*, PICPA – *Pichia pastoris*, CANBO – *Candida boidinii*, CANAL – *Candida albicans*, DEBHA – *Debaryomyces hansenii*, CLALU – *Clavispora lusitaniae*.

Di Tommaso P, Moretti S, Xenarios I, Orobitg M, Montanyola A, Chang J-M, Taly J-F, Notredame C. 2011. T-Coffee: a web server for the multiple sequence alignment of protein and RNA sequences using structural information and homology extension. *Nucleic Acids Research* **39**:W13–W17. doi:10.1093/nar/gkr245
