# Supplementary material for: Telomere length regulation by Rif1 protein from Hansenula polymorpha
Source: eLife. 2022 Feb 7;11:e75010. doi: 10.7554/eLife.75010 (PMC8820739; doi:10.7554/eLife.75010)
Supplement: Supplementary file 2. [file elife-75010-supp2.docx]

HANPO 1 MSANDN--------------DTGRTSPTVIVAPTTSGRDKADRLGKKFADAKRNNRSRFA
OGAPO 1 MSANDN--------------DTGRTSPTVIVAPTTSGRDKADRLGKKFADAKRNNRSRFA
OGAPH 1 MFYSILPLKGSQENSPE-MENTTSTSPTARLSPSMSGRDKANLLDRKFADAKRNSRSKFA
OGAKO 1 MFLNKEPSSIDR-RSPSQWTEEDKQIPTVILAPTLSGRDKADRLGKKFADAKKNSRNKFS
OGAMI 1 MFLDKQPTSIDR-RSMS-PGDKDKPSPMVILAPTSSGRDKADRLGKKFADAKRNSRSRFA
OGANOn 1 MFLDKQTTSIER-RSMS-PSDKDKPSPIVILAPTSSGRDKADRLGKKFADAKRNSRSRFS

HANPO 47 PSRSAESPYNVLT-SPSKNDPVKLSRLYSIMNSKRST-NNKSKRQREEP-AL-QRSA---
OGAPO 47 PSRSAESPYNVLT-SPSRNDPVKLSRLYSIMNSKRTS-NNKSRRQREEP-AL-QRAA---
OGAPH 60 ATKHQESSPQPSTA-PSGNDPVKLQRIYSILNAKRST-PSKAPQPQEQRSSSGENGLAAK
OGAKO 60 AARNGTSPDRALAATQSKNDPVKLNKLYSILNSKRTS-NSKKPTADVSD-GF-IRTRARR
OGAMI 59 LSRSAESPNNVLNTSSSRSDPVKLNKLYSILNSKKRQTRSKKQ--KQSPSGS-RQSPSSE
OGANOn 59 LSRSAESPYNVLNAPSSKSDPVKLNKLYSILNSKKRPAKSRKQ--KQTAATALQQVPPAE

HANPO 100 --SDISQAQVSTDENA--SIPQDA-LVTRSQPA-----IATPNIVDDPSTTDPRAGEQ--
OGAPO 100 --SDISQAKVSPDENV--GSPHNA-LVTRSQPA-----IATPIVLDDPSTTSLLADEQ--
OGAPH 118 ---------STSSQETTLGLRSGVRDASSLGLASLNSAASYRDLMIAKRNAFKQRMIQNE
OGAKO 117 ALSEVDQA--STDATS--AQPQ----------------TYQEIVR---------ARKR--
OGAMI 116 SI-------QQTDPET--SIESDA-PISSLENTNLSPQALYKNVLIAKRNAFKEQLEKSE
OGANOn 117 PV-------RETDPET--TIDSDV-PIPSLNNDNVSPQALYKNVLIAKRNAFKEQIEKSQ

HANPO 148 --------------------------------------SGPSPNKSMESKSQSQSKALQT
OGAPO 148 --------------------------------------SGSSPNKSVVSTPQSQSKAPQT
OGAPH 169 AAMRSAAEDSAETTIEAQPPKPDSQPPVKSLLDHAPVQETPVPDTPRETRQARAAVEKQT
OGAKO 146 --------------------------------------ARATRSLSVELTPAKPQPIPQT
OGAMI 166 --------------------------------------RSASPK--ITERSSSSSAQPET
OGANOn 167 --------------------------------------KEIPPDTNLKSHDEQISPIVEG

HANPO 170 APNGNSQSTPTKGSQP-------SPSQFGSNN------T-HLAPGQRHLRISKQAFEKNI
OGAPO 170 ARNGSS--------QP-------SPTQFASND------T-HLAPGQHHTCISKQAFEKNI
OGAPH 229 VKSARKRSSPSKSPSPEKTPSKVTKTVPASEAHTPSNSRPSSAPSSGRTRRATRGPVPRA
OGAKO 168 PA----------------------------------------------------------
OGAMI 186 VSMSQQASAPSI-----------------------------IADPQRESQWSNNSPKERS
OGANOn 189 SPSVVSQQEPSQEPED---SEAISVSQPTSVS------K-KVAAPARRSRSYNHSPNPKI

HANPO 216 AQ----IS------DNISTFQAVVRNPARTSHTD--------------------------
OGAPO 208 AQ----IS------DTISTFQAVVRNPARTSHAD--------------------------
OGAPH 289 PFQGPAIPAAAATAIKSSSFANVTRASDRTSAA------VISED----------LSPAEK
OGAKO 170 ------KG------VQVSPFQNIVRSPKQNNVSP------RPEE----------EKP---
OGAMI 217 TTCNNTGQ------SLLAALKNVVRNTKLPS-PQCTPPPPEQITEPTDVIEVPVEGQ---
OGANOn 239 PVSNPAGN------SLLAAFKNVVKNATLTRSSECEPATADTTS----------KKP---

HANPO 240 -----------QQSSAEKTPKE------------P-AVPTPQTPTRQAGS---------R
OGAPO 232 -----------QQPSAQNPPKE------------P-TAPTPQTPTRQAVF---------Q
OGAPH 333 SRKTMSDSELHAKRIEEKTKPEFSAVEPQPTIIEKETLQVDSTPQKPASNGPGLFVEDTD
OGAKO 199 --------EHHQESIAETQQQT------------SDLPDLPSTPQTSATR---------A
OGAMI 267 --------EAQETTGIVTTKST----TDQSSESLSQTAVEPSTPLSRPVP---------A
OGANOn 280 --------EVQQQSVVATTKSA----LEQPAQSQNETIIEPSTPQKRPIG---------A

HANPO 267 DAAGNQTGSDKKVLFSGNVDKSPIASSPIKEPYEPRSILK
OGAPO 259 DRNGNQTGSDKKVLFSSNVDKSPIASSPIKEPSEPRSILK
OGAPH 393 -NDDDVPLSAKRVLFSSDIELSPPAQEPAKTIAEPKSILK
OGAKO 230 -LQNPASTSAKRVLFSAEIDRSPVGSSPIKSPEEPRSILK
OGAMI 306 -LQEDIQQSAKRVLFSSDVEDSPTASSPIREAMEPRSILK
OGANOn 319 -LQEDVQQSVKRVLFSSDVEESPTASSPIREPLEPRSILK

**Supplementary File 2.** Multiple alignment of Rif1_NTE_ regions from *H. polymoprha* DL-1 (HANPO) and five of its closest relatives. Sequences were aligned using T-Coffee and shaded with BoxShade. OGAPO – *Ogataea polymorpha*, OGAPH – *Ogataea philodendri*, OGAKO – *Ogataea kodamae*, OGAMI – *Ogataea minuta*, OGANOn – *Ogataea nonfermentans*.
