# Supplementary material for: Telomere length regulation by Rif1 protein from Hansenula polymorpha
Source: eLife. 2022 Feb 7;11:e75010. doi: 10.7554/eLife.75010 (PMC8820739; doi:10.7554/eLife.75010)
Supplement: Supplementary file 3. [file elife-75010-supp3.docx]

**Supplemental Table S1**. *H. polymorpha* strains used in this study.

| **Strain name** | **Genotype** | **Reference** |
| --- | --- | --- |
| DL1-L | DL-1 (ATCC 26012) *leu2* | (Kang et al., 2005) |
| *∆rad52* | DL-1 (ATCC 26012) *leu2 ∆rad52::G418* | This study |
| *∆ter* | DL-1 (ATCC 26012) *leu2 ∆ter::G418* | This study |
| *∆rif1* | DL-1 (ATCC 26012) *leu2 ∆rif1::HpLEU2* | This study |
| *∆rif1∆rad52* | DL-1 (ATCC 26012) *leu2 ∆rif1::HpLEU2 ∆rad52::G418* | This study |
| *∆rif1∆ter* | DL-1 (ATCC 26012) *leu2 ∆rif1::HpLEU2 ∆ter::G418* | This study |
| Rif1-HA | DL-1 (ATCC 26012) *leu2 ∆ade2 ∆ura3::ADE2 Rif1-3HA::HpURA3* | This study |
| Rif1*-HA | DL-1 (ATCC 26012) *leu2 ∆ade2 ∆ura3::ADE2*  *Rif1-3HA::HpURA3::HpLEU2* | This study |
| Rif1_NTE_^1-264^-HA | DL-1 (ATCC 26012) *leu2 ∆ade2 ∆ura3::ADE2*  *rif1∆265-1521-3HA::HpURA3* | This study |
| Rif1*^∆^*^NTE^-HA | DL-1 (ATCC 26012) *leu2 ∆ade2 ∆ura3::ADE2*  *rif1∆1-264-3HA::HpURA3::HpLEU2* | This study |
| 4A^1^Rif1*-HA | DL-1 (ATCC 26012) *leu2 ∆ade2 ∆ura3::ADE2*  *rif1(K38A, R39A, R42A, R44A)-3HA::HpURA3::HpLEU2* | This study |
| 4A^2^Rif1*-HA | DL-1 (ATCC 26012) *leu2 ∆ade2 ∆ura3::ADE2*  *rif1(K79A, R80A, K85A, K87A)-3HA::HpURA3::HpLEU2* | This study |
| 8ARif1*-HA | DL-1 (ATCC 26012) *leu2 ∆ade2 ∆ura3::ADE2*  *rif1(K38A, R39A, R42A, R44A, K79A, R80A, K85A, K87A)-3HA::HpURA3::HpLEU2* | This study |
| Rif1-HA*∆ku70* | DL-1 (ATCC 26012) *leu2 ∆ade2 ∆ura3::ADE2 Rif1-3HA::HpURA3 ∆ku70::HpLEU2* | This study |
| Rif1-HA*∆ku80* | DL-1 (ATCC 26012) *leu2 ∆ade2 ∆ura3::ADE2 Rif1-3HA::HpURA3 ∆ku80::G418* | This study |
| Rif1*^F225E^-HA | DL-1 (ATCC 26012) *leu2 ∆ade2 ∆ura3::ADE2*  *rif1(F225E)-3HA::HpURA3::HpLEU2* | This study |
| Rif1*^R230E^-HA | DL-1 (ATCC 26012) *leu2 ∆ade2 ∆ura3::ADE2*  *rif1(R230E)-3HA::HpURA3::HpLEU2* | This study |
| Rif1*^F225E/R230E^-HA | DL-1 (ATCC 26012) *leu2 ∆ade2 ∆ura3::ADE2*  *rif1(F225E, R230E)-3HA::HpURA3::HpLEU2* | This study |
| 6ARif1*-HA | DL-1 (ATCC 26012) *leu2 ∆ade2 ∆ura3::ADE2*  *rif1(F225A, Q226A, V228A, V229A, R230A)-3HA::HpURA3::HpLEU2* | This study |
| Rif1_NTE_^1-264(F225E)^-HA | DL-1 (ATCC 26012) *leu2 ∆ade2 ∆ura3::ADE2*  *rif1∆265-1521(F225E)-3HA::HpURA3* | This study |
| Rif1_NTE_^1-264(R230E)^-HA | DL-1 (ATCC 26012) *leu2 ∆ade2 ∆ura3::ADE2*  *rif1∆265-1521(R230E)-3HA::HpURA3* | This study |
| Rif1-FlagKu80-HA | DL-1 (ATCC 26012) *leu2 ∆ade2 ∆ura3::ADE2 Rif1-3Flag::HpLEU2 Ku80-3HA::HpURA3* | This study |
| Rif1-FlagKu80-HA*∆ku70* | DL-1 (ATCC 26012) *leu2 ∆ade2 ∆ura3::ADE2 Rif1-3Flag::HpLEU2 Ku80-3HA::HpURA3 ∆ku70::G418* | This study |
| Ku80-HA*∆ku70* | DL-1 (ATCC 26012) *leu2 ∆ade2 ∆ura3::ADE2 Ku80-3HA::HpURA3 ∆ku70::G418* | This study |
| Ku70-HA | DL-1 (ATCC 26012) *leu2 ∆ade2 ∆ura3::ADE2 Ku70-3HA::HpURA3* | This study |
| Ku80-HA | DL-1 (ATCC 26012) *leu2 ∆ade2 ∆ura3::ADE2 Ku80-3HA::HpURA3* | This study |
| *∆ku70* | DL-1 (ATCC 26012) *leu2 ∆ku70::HpLEU2* | This study |
| *∆ku80* | DL-1 (ATCC 26012) *leu2 ∆ku80::G418* | This study |
| *∆rif1∆ku80* | DL-1 (ATCC 26012) *leu2 ∆rif1::HpLEU2 ∆ku80::G418* | This study |
| Rif1*-HA*∆ku80* | DL-1 (ATCC 26012) *leu2 ∆ade2 ∆ura3::ADE2*  *Rif1-3HA::HpURA3::HpLEU2 ∆ku80::G418* | This study |
| Rif1*^∆^*^NTE^-HA*∆ku80* | DL-1 (ATCC 26012) *leu2 ∆ade2 ∆ura3::ADE2*  *rif1∆1-264-3HA::HpURA3::HpLEU2 ∆ku80::G418* | This study |
| Rif1-FlagStn1-HA | DL-1 (ATCC 26012) *leu2 ∆ade2 ∆ura3::ADE2 Rif1-3Flag::HpLEU2 Stn1-3HA::HpURA3* | This study |
| Stn1-HA | DL-1 (ATCC 26012) *leu2 ∆ade2 ∆ura3::ADE2 Stn1-3HA::HpURA3* | This study |
| Stn1-FlagRif1-HA | DL-1 (ATCC 26012) *leu2 ∆ade2 ∆ura3::ADE2 Rif1-3HA::HpURA3 Stn1-3Flag::HpLEU2* | This study |
| Stn1^∆C381^-FlagRif1-HA | DL-1 (ATCC 26012) *leu2 ∆ade2 ∆ura3::ADE2 Rif1-3HA::HpURA3 stn1∆381-599-3Flag::HpLEU2* | This study |
| Stn1^∆C221^-FlagRif1-HA | DL-1 (ATCC 26012) *leu2 ∆ade2 ∆ura3::ADE2 Rif1-3HA::HpURA3 stn1∆221-599-3Flag::HpLEU2* | This study |
| Stn1-Flag | DL-1 (ATCC 26012) *leu2 Stn1-3Flag::HpLEU2* | This study |
| Stn1^∆C381^-Flag | DL-1 (ATCC 26012) *leu2 stn1∆381-599-3Flag::HpLEU2* | This study |
| Stn1^∆C221^-Flag | DL-1 (ATCC 26012) *leu2 stn1∆221-599-3Flag::HpLEU2* | This study |
| Stn1-HA*∆rif1* | DL-1 (ATCC 26012) *leu2 ∆ade2 ∆ura3::ADE2 Stn1-3HA::HpURA3 ∆rif1::HpLEU2* | This study |
| Stn1-HA*∆ku70* | DL-1 (ATCC 26012) *leu2 ∆ade2 ∆ura3::ADE2 Stn1-3HA::HpURA3 ∆ku70::HpLEU2* | This study |
| Rif1-HAintTEL0 | DL-1 (ATCC 26012) *leu2 ∆ade2 ∆ura3::ADE2 leu2::G418^R^ Rif1-3HA::HpURA3* | This study |
| Rif1-HAintTEL18 | DL-1 (ATCC 26012) *leu2 ∆ade2 ∆ura3::ADE2 leu2::G418^R^(HARS36PstI-BclI) Rif1-3HA::HpURA3* | This study |
| Rap1B-HAintTEL0 | DL-1 (ATCC 26012) *leu2 ∆ade2 ∆ura3::ADE2 leu2::G418^R^ Rap1B-3HA::HpURA3* | This study |
| Rap1B-HAintTEL18 | DL-1 (ATCC 26012) *leu2 ∆ade2 ∆ura3::ADE2 leu2::G418^R^(HARS36PstI-BclI) Rap1B-3HA::HpURA3* | This study |
| Rif ^1K504E^-HA | DL-1 (ATCC 26012) *leu2 ∆ade2 ∆ura3::ADE2 rif1(K504E)-3HA::HpURA3* | This study |
| Rif ^1R539E^-HA | DL-1 (ATCC 26012) *leu2 ∆ade2 ∆ura3::ADE2 rif1(R539E)-3HA::HpURA3* | This study |
| Rif ^1K504E/R539E^-HA | DL-1 (ATCC 26012) *leu2 ∆ade2 ∆ura3::ADE2 rif1(K504E, R539E)-3HA::HpURA3* | This study |
| Rif ^1K658E/K666E^-HA | DL-1 (ATCC 26012) *leu2 ∆ade2 ∆ura3::ADE2 rif1(K658E, K666E)-3HA::HpURA3* | This study |
| Rif1-HA*∆ter* | DL-1 (ATCC 26012) *leu2 ∆ade2 ∆ura3::ADE2 Rif1-3HA::HpURA3 ∆ter::HpLEU2* | This study |
| Ku80-HA*∆rif1* | DL-1 (ATCC 26012) *leu2 ∆ade2 ∆ura3::ADE2 Ku80-3HA::HpURA3 ∆rif1::HpLEU2* | This study |
| TERT-HA | DL-1 (ATCC 26012) *leu2 ∆ade2 ∆ura3::ADE2 TERT-3HA::HpURA3* | (Malyavko et al., 2019) |
| CDC13^+/-^ | CBS4732 (ATCC 34438) *leu2/leu2 ade2/ade2 URA3/ura3::ADE2 CDC13/cdc13::HpLEU2* | This study |
| STN1^+/-^ | CBS4732 (ATCC 34438) *leu2/leu2 ade2/ade2 URA3/ura3::ADE2 STN1/stn1::HpLEU2* | This study |
| TEN1^+/-^ | CBS4732 (ATCC 34438) *leu2/leu2 ade2/ade2 URA3/ura3::ADE2 TEN1/ten1::HpLEU2* | This study |
| KU70^+/-^ | CBS4732 (ATCC 34438) *leu2/leu2 ade2/ade2 URA3/ura3::ADE2 KU70/ku70::HpLEU2* | This study |
